# ATLANTIC SPATIAL: A dataset of landscape, topographic, hydrological, and anthropogenic metrics for the Atlantic Forest

**DOI:** 10.1002/ecy.70360

**Published:** 2026-04-10

**Authors:** Maurício Humberto Vancine, Bernardo Brandão Niebuhr, Renata L. Muylaert, Júlia de Oshima, Vinicius Tonetti, Rodrigo Bernardo, Rafael Souza Cruz Alves, Eduardo Miguel Zanette, Victor Casagrande Souza, João Gabriel Ribeiro Giovanelli, John Wesley Ribeiro, Carlos De Angelo, Carlos Henrique Grohmann, Mauro Galetti, Milton Cezar Ribeiro

**Affiliations:** ^1^ Departamento de Biodiversidade, Laboratório de Ecologia Espacial e Conservação Universidade Estadual Paulista (UNESP), Instituto de Biociências Rio Claro São Paulo Brazil; ^2^ Norwegian Institute for Nature Research (NINA) Oslo Norway; ^3^ Sydney School of Veterinary Science, Faculty of Science The University of Sydney Sydney New South Wales Australia; ^4^ Departamento de Ecologia, Laboratório de Ecologia do Movimento Universidade de São Paulo, Instituto de Biociências São Paulo São Paulo Brazil; ^5^ Center for Natural Sciences, Federal University of São Carlos Buri Brazil; ^6^ Departamento de Biodiversidade, Laboratório de Primatologia Universidade Estadual Paulista (Unesp), Instituto de Biociências Rio Claro São Paulo Brazil; ^7^ Departamento de Biodiversidade, Laboratório de Biologia da Conservação Universidade Estadual Paulista (UNESP), Instituto de Biociências Rio Claro São Paulo Brazil; ^8^ Seleção Natural (SN), Rua Cezira Giovanoni Moretti Piracicaba São Paulo Brazil; ^9^ Instituto de Ciencias de la Tierra, Biodiversidad y Ambiente (Universidad Nacional de Río Cuarto ‐ CONICET) Río Cuarto Córdoba Argentina; ^10^ Universidade de São Paulo, Institute of Astronomy, Geophysics and Atmospheric Sciences (IAG), Spatial Analysis And Modelling Lab (SPAMLab) São Paulo Brazil; ^11^ Kimberly Green, Latin American and Caribbean Center, Florida International University (FIU) Miami Florida USA; ^12^ Environmental Studies Center (CEA), Laboratório de Ecologia Espacial e Conservação Universidade Estadual Paulista (Unesp) Rio Claro São Paulo Brazil

**Keywords:** biodiversity hotspot, habitat loss, habitat fragmentation, land cover, land use, rainforest, raster, spatial ecology, tropical ecology

## Abstract

Space is one of the main drivers of biodiversity, as it regulates the underlying processes affecting the distribution and dynamics of species and communities. It is a fundamental factor when considering the rapid climate and land cover changes occurring at local and global scales, which are linked to habitat loss and fragmentation, as well as their impacts on biodiversity. The Atlantic Forest of South America is among the world's biodiversity hotspots because of its exceptionally high species richness and endemism. Most of the threats to the Atlantic Forest's biodiversity stem from the expansion of urbanization and industry, extensive agricultural and livestock production, and mining. Here, we provide integrated and fine‐scale spatial information (30‐m resolution) for the entire extent of the Atlantic Forest for the years 2020 to 2022. The spatial data include different vegetation classes (forest, and forest combined with other non‐forest vegetation), the effects of linear structures (roads and railways), and landscape metrics computed at multiple scales (radius buffers—moving window sizes—ranging from 50 to 2500 m, and up to 10 km for some metrics). The dataset comprises the Atlantic Forest delimitation vector and more than 500 rasters, available through a series of thematically grouped files in multiple Zenodo repositories. This data can also be accessed using the R package *atlanticr*, which we developed to facilitate data retrieval and organization from Zenodo. The dataset includes landscape, topographic, hydrological, and anthropogenic metrics. Landscape metrics were calculated for two vegetation classes—Forest Vegetation (which combined different forest cover classes) and Natural Vegetation (which combined forest and non‐forest cover classes)—as well as for a heterogeneous, multi‐class classification of the landscape (31 land cover classes). The landscape metrics include landscape morphology (classification as matrix, core, edge, corridor, branch, stepping stone, and perforation), fragment area and proportion, patch area and number, edge and core areas and proportions, structural and functional connectivity (for different organisms' gap‐crossing capabilities), distance to and from fragment edges, fragment perimeter and perimeter–area ratio, and landscape diversity (heterogeneity). Topographic metrics include elevation, slope, aspect, curvature, and landform elements (peak, ridge, shoulder, spur, slope, hollow, footslope, valley, pit, and flat). Hydrological metrics comprise potential springs (and their kernel density) and streams (and distance to the nearest feature). Anthropogenic metrics include maps of roads, railways, protected areas, Indigenous territories, and quilombola territories (localities of self‐defined Afro‐Brazilian traditional communities), as well as the distance to each feature. This dataset facilitates the efficient integration of biodiversity and spatially explicit data for the Atlantic Forest, serving as a data source for studies, landscape planning, biodiversity conservation, and forest restoration programs. The data are released under a CC BY‐NC 4.0: Creative Commons Attribution‐Non‐Commercial 4.0 International license and this data paper should be cited when the data are reused.

## CONFLICT OF INTEREST STATEMENT

The authors declare no conflicts of interest.

## Supporting information


Data S1.

## Data Availability

A summary document is provided as Supporting Information in Data S1. Due to size, the complete dataset (vector and rasters) is provided via multiple Zenodo repositories (see Metadata S1: Table 7 in the DataS1.zip file in the Supporting Information). Code used to calculate the metrics is available on Zenodo at https://doi.org/10.5281/zenodo.14814102. All landscape metrics were calculated using custom functions based on LSMetrics and translated to R; this material is available in Zenodo at https://doi.org/10.5281/zenodo.3736443.

