# Supplementary material for: ATLANTIC SPATIAL: A dataset of landscape, topographic, hydrological, and anthropogenic metrics for the Atlantic Forest
Source: Ecology. 2026 Apr 10;107(4):e70360. doi: 10.1002/ecy.70360 (PMC13067800; doi:10.1002/ecy.70360)
Supplement: Supplementary file 1 — Data S1. [file ECY-107-e70360-s001.zip › ecy70360-sup-0001-DATAS1/Metadata_S1.pdf]

Data Paper published in *Ecology*, an Ecological Society of America journal

Metadata S1

ATLANTIC SPATIAL: A dataset of landscape, topographic, hydrological, and anthropogenic metrics for the Atlantic Forest

Maurício Humberto Vancine, Bernardo Brandão Niebuhr, Renata L. Muylaert, Júlia Emi de Faria Oshima, Vinicius Tonetti, Rodrigo Bernardo, Rafael Souza Cruz Alves, Eduardo Miguel Zanette, Victor Casagrande Souza, João Gabriel Ribeiro Giovanelli, John Wesley Ribeiro, Carlos De Angelo, Carlos Henrique Grohmann, Mauro Galetti, Milton Cezar Ribeiro

Introduction

In ecology, space matters. Space affects the main drivers of biodiversity since it regulates the underlying processes influencing the distribution and dynamics of species (Fletcher and Fortin 2018). These ecological processes, such as environmental filtering, biotic interactions, dispersal, and ecological drift, drive the response of organisms to environmental factors such as climate, topography, soil types, land use and land cover (LULC), and habitat connectivity and heterogeneity (Anderle et al. 2022, Messager et al. 2023). Thus, space is a fundamental component when we consider the rapid effects of climate and LULC changes at local, regional, and global scales, as well as their widespread consequences for habitat loss and fragmentation (Jetz et al. 2007, He et al. 2019, Williams and Newbold 2020, Ma et al. 2023, Gonçalves-Souza et al. 2025a, Zou et al. 2025). Having available geospatial environmental data is essential to assess the effects of these changes on biodiversity at different spatial and temporal scales, extents, and grains (e.g., Lima-Ribeiro et al. 2015, Vega et al. 2017, Fick and Hijmans 2017, Souza et al. 2020, Karger et al. 2020, Poggio et al. 2021, Potapov et al. 2022b, 2022a, Hawker et al. 2022, Hansen et al. 2022, Tang and Werner 2023, Gonçalves-Souza et al. 2025b). Comprehensive spatial datasets are therefore important to address conservation and restoration efforts to maintain biodiversity and its ecological processes (Dirzo et al. 2014, He et al. 2015, Young et al. 2016, Johnson et al. 2017). Ultimately, such datasets would help to unravel frequent 'spatial complications' [i.e., the neglected contribution of space in explaining ecological processes] (Kareiva 1994) in ecology and other geospatial disciplines.

Habitat loss and fragmentation are currently major threats to biodiversity and ecological processes worldwide (Fahrig 2003, Haddad et al. 2015, Chase et al. 2020). Landscape composition and configuration are essential factors determining biodiversity, population dynamics, species interactions, dispersal, and the functions that biota performs across space (Fahrig 2003, Driscoll et al. 2013, Duflot et al. 2017). Different landscape metrics can be used as proxies of landscape heterogeneity to predict biodiversity and ecosystem function (Tonetti et al. 2023). This is especially relevant in fragmented landscapes where natural vegetation fragments are surrounded by different land cover types (Fischer and Lindenmayer 2007, Turner and Gardner 2015). Landscape metrics can also be used to identify priority areas for conservation (Tambosi et al. 2014) and to predict species' potential distributions (Fletcher et al. 2016, Riva et al. 2024). Furthermore, these metrics must be computed considering different

scales depending on the phenomenon of interest (Šímová and Gdulová 2012, Jackson and Fahrig 2015, Miguet et al. 2016, Beale et al. 2025, Muylaert et al. 2025), and the specific species' functional responses to landscape structure (Mimet et al. 2013, Riva and Nielsen 2020, Riva and Nielsen 2021, Niebuhr et al. 2023).

The Atlantic Forest of South America is among the global biodiversity hotspots due to its high species richness and endemism associated with severe habitat loss (Myers et al. 2000, Sloan et al. 2014). The Atlantic Forest covers almost the entire coast of Brazil and reaches inland portions of the continent in parts of Paraguay and Argentina, and its vegetation covered over 1.6 million km^2 before the European colonization (Marques et al. 2021). Due to its wide longitudinal, latitudinal, and altitudinal range, the Atlantic Forest has high environmental heterogeneity with different vegetation types generated mainly by the rainfall distribution, from its coast as mangrove and sandy coastal plain vegetation (*restinga*), passing through humid forest (dense ombrophilous, open ombrophilous, mixed ombrophilous; ombrophilous—vegetation domain that tolerates or thrives in wet conditions), dry forest formations (semideciduous and deciduous seasonal), in addition to other non-forest environments, as natural grasslands (Joly et al. 2014). These geographical characteristics, combined with large topographic variability and paleoecology process of formation, favored high species diversification rates and endemism (Carnaval et al. 2014, Peres et al. 2020). Fossil and phylogenetic evidence indicates that the humid-forest system that includes the Atlantic Forest was established by the Paleocene–Eocene (66–34 Ma) (Burnham and Johnson 2004).

The high diversification rate in the Atlantic Forest is evidenced by its high biodiversity: it contains almost 18,000 species of plants (Flora e Funga do Brasil 2023); 2,645 species of Tetrapoda (Figueiredo et al. 2021); around 1,000 species of fish (Reis et al. 2016); 1400 species of social insects (Feitosa et al. 2021); more than 2,000 species of butterflies (Iserhard et al. 2017); and more than 112,000 species of arachnids (Giupponi et al. 2017). The Atlantic Forest directly provides ecosystem services for >150 million people, such as water provisioning and regulation, hydroelectric energy generation, food production, pollination, soil protection, climate regulation, carbon storage, air quality, and cultural services (Joly et al. 2014, Pires et al. 2021). Much of the Atlantic Forest biodiversity is highly threatened, especially birds (Bonfim et al. 2021), small mammals (Palmeirim et al. 2019), medium and large mammals (Rios et al. 2021b), and amphibians (Almeida-Gomes and Rocha 2014). Furthermore, ecological processes are also affected by landscape modifications, such as interaction networks

(Marjakangas et al. 2020, Monteiro et al. 2022), carbon storage (Bello et al. 2015, de Lima et al. 2020, Pyles et al. 2022), and pollination (Varassin et al. 2021). In addition, other threats to the Atlantic Forest landscapes include defaunation (Galetti et al. 2017, 2021), the introduction of non-native species (Vitule et al. 2021), and climate change (Scarano and Ceotto 2015, Vale et al. 2021).

The Atlantic Forest covers three countries (Argentina, Brazil, and Paraguay) with the large deforestation between 1986 and 2005 (Vancine et al. 2024). Thus, landscape modifications within the Atlantic Forest have caused strong impacts; even though the current deforestation rates have decreased compared to earlier decades, a series of studies still shows a warning scenario in terms of habitat loss and fragmentation (de Lima et al. 2020, 2024, Carlucci et al. 2021, Vancine et al. 2024). For example, although the Atlantic Forest has gained around 1 million hectares in the last 20 years (Vancine et al. 2024), many negative impacts have occurred, such as illegal deforestation (Amaral et al. 2025), and the pronounced effect of fires (Adorno et al. 2025). Despite a recent temporal stability of around 28 million hectares of forest, a considerable part of this forest is composed mostly of secondary forest (Rosa et al. 2021). Furthermore, although recent estimates indicate that about 23% of forests and 36% of natural vegetation remains in the Atlantic Forest, much of it is highly fragmented: 97% of fragments are smaller than 50 ha, 60% of forests are affected by edge effects (within 90 m), and vegetation fragments are highly isolated, with an average distance of 250 to 830 m between then) (Vancine et al. 2024). Moreover, the high density of linear infrastructure (roads and railways) affects the vegetation remnants—especially the large ones (>500,000 ha), proving to have a major negative impact on biodiversity (Cassimiro et al. 2023, Vancine et al. 2024).

The Atlantic Forest is one of the most intensely studied biomes in the world. A large initiative coordinated by Brazilian researchers – *ATLANTIC: Data Papers from a biodiversity hotspot* – has compiled hundreds of thousands of records of occurrence and abundance of animal and plant species in the Atlantic Forest (https://esajournals.onlinelibrary.wiley.com/doi/toc/10.1002/(ISSN)1939-9170.AtlanticPapers). Biodiversity data were collected for decades and have been recently synthesized for the Atlantic Forest in numerous ATLANTIC data papers (Bello et al. 2017, Bovendorp et al. 2017, Figueiredo et al. 2017, Lima et al. 2017, Muylaert et al. 2017, Gonçalves et al. 2018a, 2018b, Hasui et al. 2018, Vancine et al. 2018, Santos et al. 2018, Souza et al. 2019,

Culot et al. 2019, Ramos et al. 2019, Rodrigues et al. 2019, Silva et al. 2022, Boscolo et al. 2023, Franceschi et al. 2024).

These biodiversity datasets have provided an unprecedented opportunity to assess how environmental conditions and species interactions affect biodiversity patterns from species to assemblages (e.g., Bovendorp et al. 2019, Palmeirim et al. 2019, Rios et al. 2021a, 2021b, Bonfim et al. 2021). However, at least three elements might limit the comparison between these studies or the increased use of this dataset to answer new questions. First, such studies rely on a highly variable set of spatial background data, which often differ in scale and grain, as well as in the source and quality of the data. Second, computing and preparing spatial data for ecological studies and impact assessments frequently require time and intensive processing, and the resources for doing that are not always available. Third, even though other studies have compiled spatial datasets for the entire world (e.g., Branco et al. 2024), these typically make layers available at coarse scales (30 arc-min or ~1 km), which are generally insufficient to understand fine-scale processes and biodiversity responses to landscape change. Once this Atlantic Forest spatial dataset becomes available, multiple studies would benefit from having a standardized and ready-to-use set of layers representing spatial variation in the landscape and human pressures, as has been demonstrated in several studies (e.g., Bovendorp et al. 2019, Palmeirim et al. 2019, Marjakangas et al. 2020, Santos et al. 2020, Rios et al. 2021b, 2021a, Bonfim et al. 2021, Monteiro et al. 2022, Anunciação et al. 2023).

We describe and provide a set of spatial datasets for the Atlantic Forest that aims to foster knowledge-building in ecology and conservation. This dataset of spatial metrics can facilitate the performance of biodiversity studies in the Atlantic Forest, allowing for more standardization and direct comparison, increasing reproducibility. The spatial datasets presented here are provided with a fine scale of 30 m, which brings high refinement to the spatial layers and the possibility of inferences at the scales where biodiversity data are collected. Thus, our aim was to facilitate the use of these spatial layers in a series of studies, within fields such as landscape ecology (Beca et al. 2017, Regolin et al. 2017, Marjakangas et al. 2020, Monteiro et al. 2022), species distribution modeling (SDMs) (Ferro e Silva et al. 2018, Bertassoni et al. 2019, Santos et al. 2020, 2022, Oshima et al. 2021, Tonetti et al. 2022, Riva et al. 2024), spatial prioritization (Tambosi et al. 2014, Rosa et al. 2021, Iezzi et al. 2022, Tonetti et al. 2024), and habitat restoration (Melo et al. 2013, Pinto et al. 2014, Zwiener et al. 2017,

Lopes et al. 2022, Piffer et al. 2022, Schweizer et al. 2022, Zupo et al. 2022, Bicudo da Silva et al. 2023).

Here, we present the ATLANTIC SPATIAL dataset, where we organize and synthesize spatial data on land cover and land use, landscape, topographic, hydrological, and anthropogenic metrics for the entire Atlantic Forest. Making this dataset available avoids complex and computationally demanding geoprocessing steps from having to be re-run and allows for different biodiversity studies to be more reproducible and potentially comparable, since they would use the same set of spatial data. We provide several metrics derived from spatial analyses using different moving window sizes and edge and gap crossing distances, so that they can be used to evaluate scales of effect on multiple scale analyses (Šímová and Gdulová 2012, Jackson and Fahrig 2015, Miguet et al. 2016, Niebuhr et al. 2023). These metrics consider a functional landscape context, which consists of using specific species' functional responses to landscape structure (Riva and Nielsen 2020, Riva and Nielsen 2021).

Moving window analysis is a widely used approach in multiscale landscape ecology studies (Hagen-Zanker 2016). It links each location (in our case, each *pixel*—picture element) to landscape patterns in a surrounding window (a square or circular neighborhood, e.g., 3x3 pixels window), with the window size potentially being used to represent the scale of effect in ecological studies (Šímová and Gdulová 2012, Jackson and Fahrig 2015, Miguet et al. 2016). Rather than producing a single summary metric, it generates a spatially explicit variable that captures how landscape structure varies across space, providing a gradient-based perspective (continuum values) even when working with categorical data (Hagen-Zanker 2016, Koen et al. 2019). Our metrics using this approach represented total habitat amount (Fahrig 2013, Fahrig 2017), habitat amount considering core and edge habitats (Haddad et al. 2015, Willmer et al. 2022, Sun et al. 2025), but also landscape diversity metrics, represented by landscape heterogeneity indices (Tonetti et al. 2023). Furthermore, considering different core/edge distances, we provide a wide variety of metrics that can be used and tested for different taxonomic groups, since not all species respond equally to fragmentation or perceive the same distance within a forest fragment as edge (Harper et al. 2005, Harper and Macdonald 2011, Harper et al. 2024). Finally, by considering multiple values of gap crossing ability—the ability of an organism to cross a certain distance to another forest fragment across the matrix—we expand the applicability of landscape metrics by associating different dispersal capacities of

organisms with functional connectivity metrics (Bélisle 2005, Awade and Metzger 2008, Baguette et al. 2013, Hatfield et al. 2018, Diniz et al. 2020).

Although we recognize the temporal limitation of the data, which were compiled for the years 2020 to 2022, we used the most recent data available when performing the calculations. In addition, this dataset can be highly relevant, since, as demonstrated by Vancine et al. (2024), the landscape structure of the Atlantic Forest has become relatively stable since 2005, with a small increase in forest vegetation (0.6% or ~1 million hectares). Thus, even future studies or those using data prior to this date can benefit from the extensive data processing we performed for this dataset.

METADATA

Class I. Dataset descriptors

A. Dataset identity

Title: ATLANTIC SPATIAL: A dataset of landscape, topographic, hydrological and anthropogenic metrics for the Atlantic Forest.

B. Dataset identification code

ATLANTIC_SPATIAL.csv (note: this is a guide to the 502 raster files (.tif) and one vector of the Atlantic Forest limit (GeoPackage - .gpkg), datasets that are too large to reproduce as part of this Data Paper; it is not the data themselves).

C. Dataset description

1. Originators

Maurício Humberto Vancine
Universidade Estadual Paulista (Unesp), Instituto de Biociências, Departamento de Biodiversidade, Laboratório de Ecologia Espacial e Conservação, Rio Claro, SP, Brazil.

Bernardo Brandão Niebuhr
Norwegian Institute for Nature Research (NINA), Oslo, Norway.

Milton Cezar Ribeiro
Universidade Estadual Paulista (Unesp), Instituto de Biociências, Departamento de Biodiversidade, Laboratório de Ecologia Espacial e Conservação, Rio Claro, SP, Brazil.

2. Abstract

Space is one of the main drivers of biodiversity, as it regulates the underlying processes affecting the distribution and dynamics of species and communities. It is a fundamental factor when considering the rapid climate and land-cover changes occurring at local and global scales, which are linked to habitat loss and fragmentation, as well as their impacts on biodiversity. The Atlantic Forest of South America is among the world's biodiversity hotspots because of its exceptionally high species richness and endemism. Most of the threats to the Atlantic Forest's

biodiversity stem from the expansion of urbanization and industry, extensive agricultural and livestock production, and mining. Here, we provide integrated and fine-scale spatial information (30 m resolution) for the entire extent of the Atlantic Forest for the year 2020 to 2022. The spatial data include different vegetation classes (forest, and forest combined with other non-forest vegetation), the effects of linear structures (roads and railways), and landscape metrics computed at multiple scales (radius buffers—moving window sizes—ranging from 50 m to 2,500 m, and up to 10 km for some metrics). The dataset comprises the Atlantic Forest delimitation vector and more than 500 rasters, available through a series of thematically grouped files in multiple Zenodo repositories. This data can also be accessed using the R package *atlanticr*, which we developed to facilitate data retrieval and organization from Zenodo. The dataset includes landscape, topographic, hydrological, and anthropogenic metrics. Landscape metrics were calculated for two vegetation classes—Forest Vegetation (which combined different forest cover classes) and Natural Vegetation (which combined forest and non-forest cover classes)—as well as for a heterogeneous, multi-class classification of the landscape (31 land-cover classes). The landscape metrics include landscape morphology (classification as matrix, core, edge, corridor, branch, stepping stone, and perforation), fragment area and proportion, patch area and number, edge and core areas and proportions, structural and functional connectivity (for different organisms' gap-crossing capabilities), distance to and from fragment edges, fragment perimeter and perimeter–area ratio, and landscape diversity (heterogeneity). Topographic metrics include elevation, slope, aspect, curvature, and landform elements (peak, ridge, shoulder, spur, slope, hollow, footslope, valley, pit, and flat). Hydrological metrics comprise potential springs (and their kernel density) and streams (and distance to the nearest feature). Anthropogenic metrics include maps of roads, railways, protected areas, Indigenous territories, and quilombola territories (localities of self-defined Afro-Brazilian traditional communities), as well as the distance to each feature. This dataset facilitates the efficient integration of biodiversity and spatially explicit data for the Atlantic Forest, serving as a data source for studies, landscape planning, biodiversity conservation, and forest restoration programs. The data are released under a CC BY-NC 4.0: Creative Commons Attribution-Non-Commercial 4.0 International license and this data paper should be cited when the data are reused.

D. Keywords

Biodiversity hotspot, habitat loss, habitat fragmentation, land cover, land use, rainforest, raster, spatial ecology, tropical ecology.

Class II. Research origin descriptors

A. Overall project description

1. Identity

A compilation of spatial covariates data of landscape, topographic, hydrological, and anthropogenic metrics for the entire Atlantic Forest at fine spatial resolution (30 m) for the year 2012 to 2023.

2. Originators

The ATLANTIC SPATIAL project was coordinated by Maurício H. Vancine and Bernardo B. Niebuhr at the São Paulo State University (UNESP), and the dataset was assembled with help from all the other authors. This is part of *ATLANTIC: Data Papers from a biodiversity hotspot*, which is led by Mauro Galetti and Milton Cezar Ribeiro at the São Paulo State University (UNESP).

3. Period of study

Dataset was processed for the time interval between 2012 and 2022. All landscape metrics were processed for the year 2020. Topographic, hydrological, and anthropogenic metrics had temporal variations between 2012 and 2022.

4. Objectives

The aim of this data paper was to provide a set of spatial covariates comprising landscape, topographic, hydrological, and anthropogenic metrics for the entire Atlantic Forest at fine spatial resolution (30 m) for the year 2012 to 2022.

5. Abstract

Same as above.

6. Sources of funding

The compilation of this dataset was supported by São Paulo Research Foundation (FAPESP) grants #2022/01899-6 (MVH), #2021/02132-8 (JEFO), #2020/11129-8 (EMZ), Coordenação de Aperfeiçoamento de Pessoal de Nível Superior (CAPES) grants fellowships 88887.513979/2020-00 and 1588183 (MVH) and Conselho Nacional do Desenvolvimento Científico e Tecnológico (CNPq) grants #130909/2020-3 (EMZ). BBN is supported by the NINA basic funding (Research Council of Norway, Grant #160022/F40). RLM is supported by an Australian Research Council Australian Laureate Fellowship (FL240100037) funded by the Australian Government. CHG is a research fellow of the National Council for Scientific and Technological Development - CNPq (grant #311209/2021-1). MCR was supported by FAPESP (processes #2013/50421-2, #2020/01779-5, #2021/08322-3, #2021/08534-0, #2021/10195-0, #2021/10639-5, #2022/10760-1) and CNPq (processes #442147/2020-1, #440145/2022-8, #402765/2021-4, #313016/2021-6, #440145/2022-8) and São Paulo State University - UNESP for their financial support. This study was financed in part by CAPES Brazil – Finance Code 001. This study is also part of the Center for Research on Biodiversity Dynamics and Climate Change, which is funded by FAPESP.

B. Specific subproject description

1. Site description

The Atlantic Forest extends from 3ºS to 33ºS, and from 35ºW to 58ºW with ~163 million hectares, covering coastal and inland portions of Brazil, Argentina, and Paraguay (Marques et al. 2021, Vancine et al. 2024) (Figure 2). Due to this large extent, the Atlantic Forest boundaries create important ecotones with other vegetation domains such as Cerrado, Caatinga, Chaco, and Pampa (Marques et al. 2021, Vancine et al. 2024). The vegetation from the Atlantic Forest is a complex mosaic composed of five main vegetation types—dense ombrophilous, open ombrophilous, mixed ombrophilous, semideciduous seasonal, and deciduous seasonal (Joly et al. 2014). The Atlantic Forest also includes mangroves and coastal scrub vegetation (Marques et al. 2021, Vancine et al. 2024). Furthermore, there are many associated ecosystems such as altitude grasslands (known as *campos rupestres* and *campos de altitude*), oceanic islands, beaches, rocky shores, dunes, marshes, inland swamps, and mountain forest (known as *brejos de altitude*) in the Northeast region (Scarano 2002). The main forest and natural vegetation changes in the Atlantic Forest include the expansion of

urbanization and industry, extensive agricultural and livestock production, and mining (Silva et al. 2016, Lembi et al. 2020, Carlucci et al. 2021, Lira et al. 2021, Viveiros de Castro et al. 2021). The Atlantic Forest is inhabited by >150 million people, most in urban areas, but also in rural areas, including indigenous communities, quilombolas traditional communities (self-defined Afro-Brazilian traditional ancestral communities descended from enslaved Africans who resisted slavery), and settlements from the Agrarian Reform and from rural social movements (Joly et al. 2014, Leite 2015, Pires et al. 2021, Viveiros de Castro et al. 2021, Shennan-Farpón et al. 2022, Benzeev et al. 2023).

2. Experimental or sampling design

Not applicable.

3. Research methods

Summary of methods

We summarized the steps for calculating and making available ATLANTIC SPATIAL metrics through a flow chart (Figure 1).

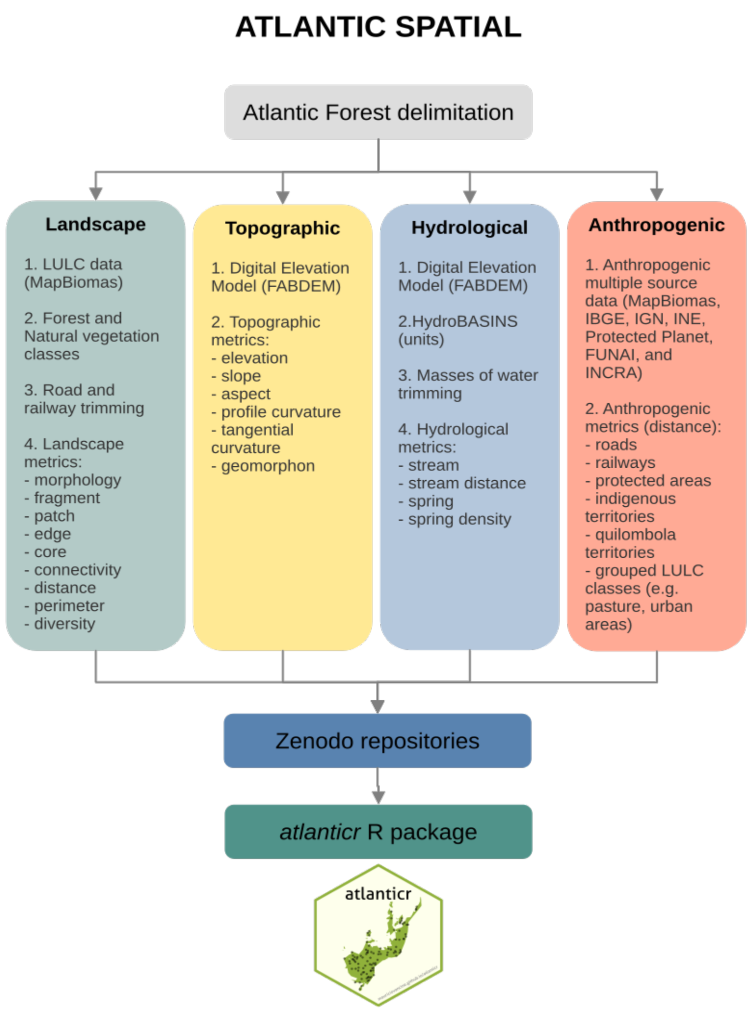

Figure 1. General workflow highlighting the most important steps to develop the ATLANTIC SPATIAL dataset.

Atlantic Forest delimitation

We used the integrative Atlantic Forest delimitation adapted from Muylaert et al. (2018) and published in Vancine et al. (2024), a general delimitation encompassing the main proposed delimitations across several associated ecosystems (Muylaert et al. 2018, Cunha et al. 2019, Marques et al. 2021). We adapted this delimitation by merging the following original maps and the most recent ones: 1. the Atlantic Forest delimitation defined by Brazilian legislation (Federal Decree No. 750/93 and Atlantic Forest Law No. 11,428/2006) named Atlantic Forest Law by IBGE (2018); 2. the Atlantic Forest limit defined by Da Silva and Casteleti (2003); 3. the Atlantic Forest delimitation defined by IBGE (2004); 4. the Atlantic Forest's most recent delimitation defined by IBGE (2019) and; 5. the Atlantic Forest

delimitation defined by Dinerstein et al. (2017) and used in the Ecoregions 2017®
(https://ecoregions.appspot.com).

We adjusted the resulting delimitation for coastal areas using the Brazilian territorial delimitation from IBGE (https://www.ibge.gov.br) for 2021, to align the limit considering the most recent delimitations of mangrove, dunes, and sandy coastal plain vegetation (*restinga*) (Scarano 2002). The final delimitation has an area total of 162,742,129 ha, which covers 3653 municipalities from 18 Brazilian states (151,470,253 ha, 93.07%), and parts of Argentina (2,668,855 ha, 1.64%) and Paraguay (8,603,022 ha, 5.29%) (Figure 2).

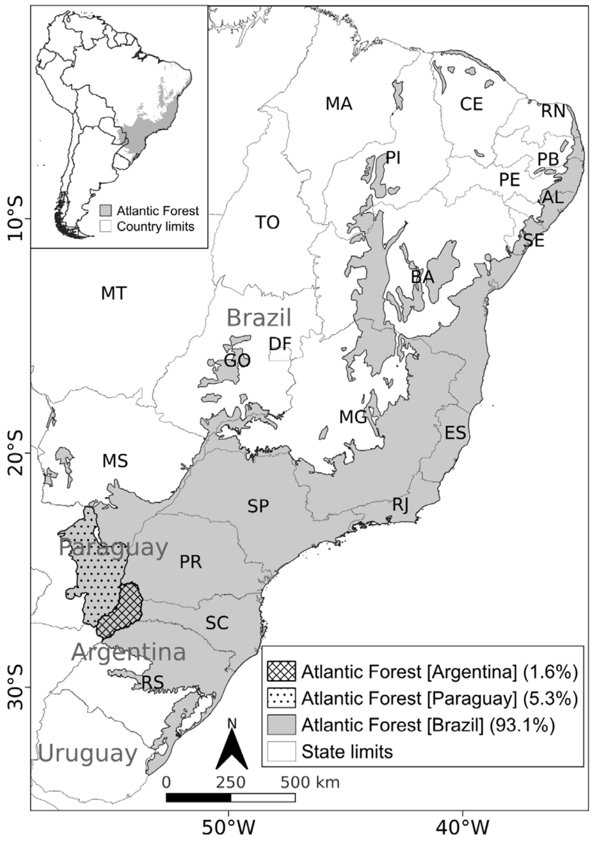

Figure 2. Integrative Atlantic Forest delimitation, adapted from Muylaert et al. (2018) and published in Vancine et al. (2024). Abbreviations are Brazilian states: MA = Maranhão, PI = Piauí, CE = Ceará, RN = Rio Grande do Norte, PB = Paraíba, PE = Pernambuco, AL = Alagoas, SE = Sergipe, BA = Bahia, MG = Minas Gerais, ES = Espírito Santo, RJ = Rio de Janeiro, SP = São Paulo, PR = Paraná, SC = Santa Catarina, RS = Rio Grande do Sul, MS = Mato Grosso do Sul, MT = Mato Grosso, GO = Goiás, DF = Distrito Federal, and TO = Tocantins.

Raster resolution and coordinate system

All geospatial datasets were rasterized with or adjusted to the resolution of 30 m (~1.8 billion cells with values). All rasters were reprojected to Albers Conical Equal Area Brazil (SIRGAS 2000) (https://brazil-data-cube.github.io/specifications/bdc-projection.html) and are therefore presented in meters.

Dataset source description

All the sources and descriptions of spatial information used to integrate spatial variables presented in the ATLANTIC SPATIAL dataset are summarized in Table 1.

Table 1. Source and description of ATLANTIC SPATIAL information.

Type of information	Institution	Description
Land Use and Land Cover (LULC)	MapBiomas	Annual LULC information at the 30-m spatial resolution from 1985 to 2021, based on pixel-based random forest classifier of Landsat satellite images using Google Earth Engine. Only the dataset for 2020 was used in the ATLANTIC SPATIAL dataset.<br><br>Source: Souza et al. (2020)<br>Site: https://mapbiomas.org<br>Date accessed: 01 November 2022

Type of information	Institution	Description
Roads and railways	Instituto Brasileiro de Geografia e Estatística (IBGE)<br><br>Instituto Geográfico Nacional (IGN)<br><br>Instituto Nacional de Estadística (INE)	Continuous Cartographic Base of Brazil, 1:250,000, for the year 2022.<br>Source: Instituto Brasileiro de Geografia e Estatística (IBGE)<br>Site: https://www.ibge.gov.br<br>Date accessed: 01 November 2022<br><br>Catalog of Geographical Objects of the Organism and forms part of the Institutional Geospatial Database, for the year 2020.<br>Source: Instituto Geográfico Nacional (IGN)<br>Site: https://www.ign.gob.ar<br>Date accessed: 01 November 2022<br><br>Digital Cartography 2012, Directorate General of Statistics, Surveys, and Censuses and is merely referential, for the year 2021.<br>Source: Instituto Nacional de Estadística (INE)<br>Site: https://www.ine.gov.py<br>Date accessed: 01 November 2022
Urban areas	MapBiomas<br><br>Instituto Geográfico Nacional (IGN)<br><br>Instituto Nacional de Estadística (INE)	Annual LULC information at the 30-m spatial resolution from 1985 to 2021, based on pixel-based random forest classifier of Landsat satellite images using Google Earth Engine. Only the dataset for 2020 was used in the ATLANTIC SPATIAL dataset.<br>Source: Souza et al. (2020)<br>Site: https://mapbiomas.org<br>Date accessed: 01 November 2022<br><br>Catalog of Geographical Objects of the Organism and forms part of the Institutional Geospatial Database, for the year 2021.<br>Source: Instituto Geográfico Nacional (IGN)

Type of information	Institution	Description
		Site: https://www.ign.gob.ar<br>Date accessed: 01 November 2022<br><br>Digital Cartography 2012, Directorate General of Statistics, Surveys, and Censuses and is merely referential, for the year 2012<br>Source: Instituto Nacional de Estadística (INE)<br>Site: https://www.ine.gov.py<br>Date accessed: 01 November 2022
Protected areas	Protected Planet	Up-to-date and complete source of data on protected areas and other effective area-based conservation measures, updated monthly with submissions from governments, non-governmental organizations, landowners, and communities, for the year 2022.<br><br>Source: Protected Planet<br>Site: www.protectedplanet.net<br>Date accessed: 01 November 2022
Indigenous territories	Fundação Nacional dos Povos Indígenas (FUNAI)<br><br>Tierras Indígenas	Official indigenist body of the Brazilian State, which promotes studies of identification, delimitation, demarcation, land regularization, and registration of lands occupied by indigenous peoples, in addition to monitoring and inspecting indigenous lands, for the year 2022.<br>Source: Fundação Nacional dos Povos Indígenas (FUNAI)<br>Site: https://www.gov.br/funai/pt-br<br>Date accessed: 01 November 2022<br><br>Interactive online platform that provides accurate maps and critical information on the lands and territories of indigenous

Type of information	Institution	Description
		peoples and communities in Paraguay, for the year 2022.<br>Source: Tierras Indígenas<br>Site:<br>https://www.tierrasindigenas.org.py/<br>Date accessed: 01 November 2022
Quilombola territories	Instituto Nacional de Colonização e Reforma Agrária (INCRA)	Brazilian federal agency responsible for implementing agrarian reform, managing public lands, and promoting the settlement and regularization of land ownership in rural areas, for the year 2020.<br><br>Source: Instituto Nacional de Colonização e Reforma Agrária (INCRA)<br>Site:<br>https://certificacao.incra.gov.br/csv_shp/export_shp.py<br>Date accessed: 01 November 2022
Topography	Forest and Buildings Removed Copernicus DEM (FABDEM) v1.2	Elevation raster map that used machine learning to remove building and tree height biases from the Copernicus GLO 30 Digital Elevation Model (DEM), for the year 2022.<br><br>Source: Hawker et al. (2022)<br>Site:<br>https://www.fathom.global/product/fabdem<br>Date accessed: 01 November 2022

Land use and land cover data

We compiled Land Use and Land Cover (LULC) maps from MapBiomas Brazil collection 7 (https://mapbiomas.org) (MapBiomas Project 2022, Souza et al. 2020), and MapBiomas Bosque Atlántico collection 2 (https://bosqueatlantico.mapbiomas.org) (MapBiomas Trinational Atlantic Forest Project, Souza et al. 2020). These datasets reconstruct

annual LULC information at the 30-m spatial resolution from 1985 to 2021, based on a pixel-based random forest classifier of Landsat satellite images processed through Google Earth Engine, and with posterior accuracy of 89.8% for the Atlantic Forest (MapBiomas Project 2022, Souza et al. 2020). We considered the LULC map for 2020 to provide the most recent data that included data validation for the previous year (2019) and subsequent year (2021), guaranteeing better accuracy for the LULC classes, in the period in which we carry out the analyses (between 2022 and 2023).

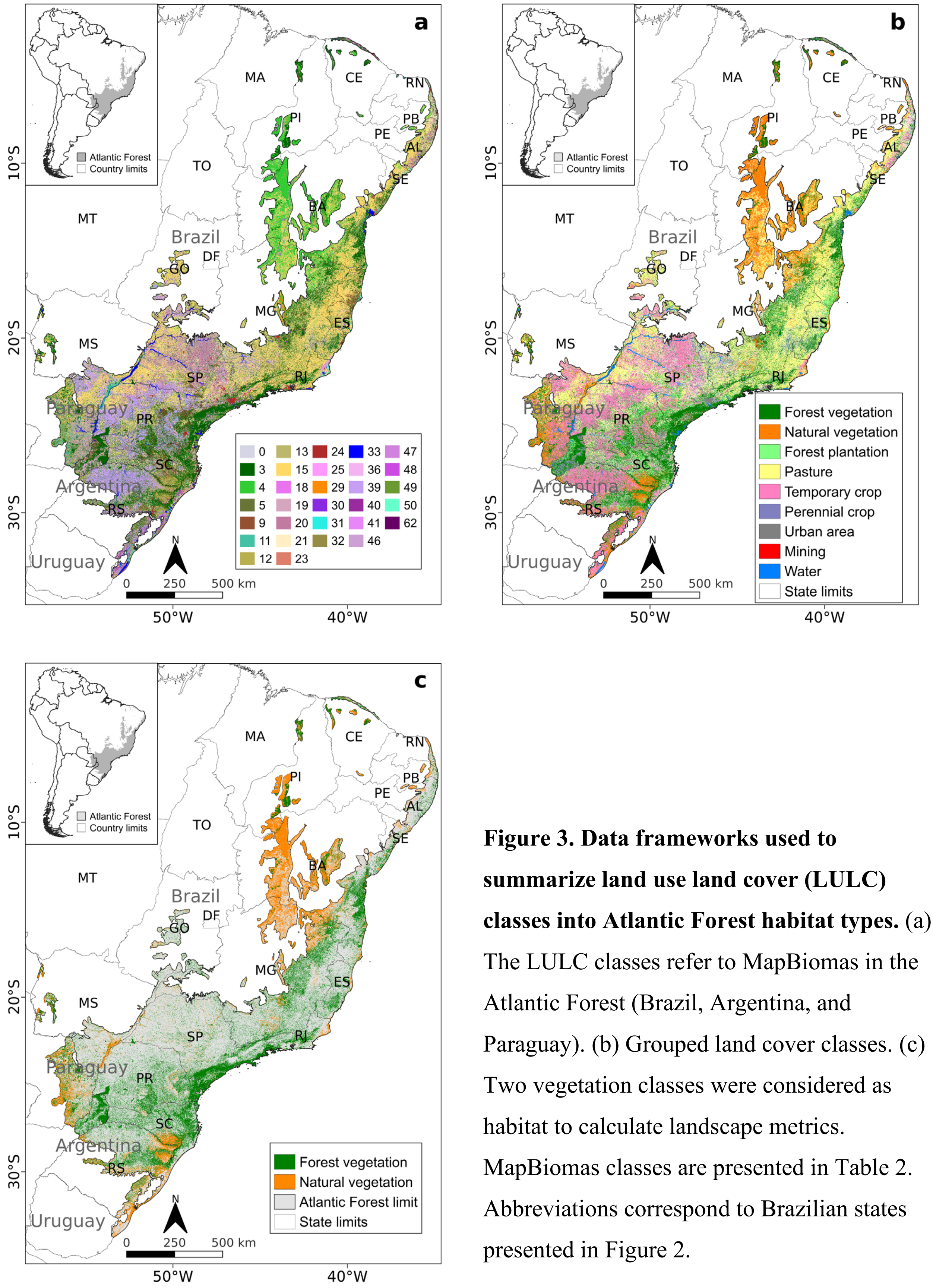

Figure 3. Data frameworks used to summarize land use land cover (LULC) classes into Atlantic Forest habitat types. (a) The LULC classes refer to MapBiomas in the Atlantic Forest (Brazil, Argentina, and Paraguay). (b) Grouped land cover classes. (c) Two vegetation classes were considered as habitat to calculate landscape metrics. MapBiomas classes are presented in Table 2. Abbreviations correspond to Brazilian states presented in Figure 2.

The LULC map from MapBiomas consists of a map with 31 classes (Table 2; Figure 3a). To calculate the Euclidean distance from land cover class metrics (i.e., values increase from LULC classes), we grouped these classes into seven broad categories: pasture, temporary crop, perennial crop, forest plantation, urban areas, mining, and water (Table 2; Figure 3b). For the landscape metrics, we defined two vegetation classes for analysis: “Forest Vegetation” selecting the land cover classes of “Forest” (forest formation, mangrove and wooded sandbank vegetation) and Natural Vegetation”, selecting the land cover classes of “Forest” and “Non-Forest Formation” (forest formation, mangrove, wooded sandbank vegetation, savanna formation, wetland, grassland, other non-forest formations, salt flat, and herbaceous sandbank vegetation) (Table 2; Figure 3c). The only exception for landscape metrics was heterogeneity, for which we used all original 31 classes from the MapBiomas LULC map in the calculation.

We adopted the terminology (Forest Vegetation and Natural Vegetation) and forest/non-forest classes defined in Vancine et al. (2024). The Atlantic Forest is composed primarily of forest formations (ombrophilous, semideciduous, and mixed forests), as well as mangroves and wooded sandbank vegetation (Scarano 2002, Joly et al. 2014). Additional vegetation types, such as Cerrado/Savanna enclaves and cleared fields, are also present within the biome, especially when considering an extensive delimitation (Vancine et al. 2024). Analyzing them separately, however, would be ecologically inconsistent, given the structural and compositional characteristics of the Atlantic Forest (Costa et al. 2023, Cavarzere and Silveira 2024). While some focal taxonomic groups are strictly forest-dependent, they may also occur in vegetation mosaics close to the biome’s boundaries with Cerrado, Pampa, and Caatinga (Costa et al. 2023, Cavarzere and Silveira 2024). Moreover, non-forest vegetation types are interspersed within forest areas, substantially altering landscape structure when analyzed jointly and influencing processes such as edge effects and habitat isolation (see details in Vancine et al. 2024). For this reason, we computed and provided datasets of metrics based on forest vegetation only, and on natural vegetation which included non-forest land cover types.

Table 2. Land use and land cover classes were grouped as land cover classes and vegetation classes. The Atlantic Forest spatial maps were based on MapBiomas collection 7. Land use and land cover class = description of the MapBiomas LULC classes; MapBiomas class code = numeric code of the MapBiomas LULC classes; Grouped land cover classes = more general classes resulting from the grouping of the LULC classes; Vegetation class =

vegetation classes resulting from the grouping of the LULC classes for the two vegetation types, Forest vegetation and Natural vegetation. Not used represents the classes not used for either grouping.

**Land use and land cover class**	**MapBiomas class code**	**Grouped land cover classes**	**Vegetation class**
Not specified	0	Not used	Not used
Forest formation	3	Forest vegetation	Forest Vegetation and Natural Vegetation
Savanna formation	4	Natural vegetation	Natural Vegetation
Mangrove	5	Forest vegetation	Forest Vegetation and Natural vegetation
Forest plantation	9	Forest plantation	Not used
Wetland	11	Natural vegetation	Natural Vegetation
Grassland	12	Natural vegetation	Natural Vegetation
Other non-forest formations	13	Natural vegetation	Natural Vegetation
Pasture	15	Pasture	Not used
Temporary crop	19	Temporary crop	Not used
Sugar cane	20	Temporary crop	Not used
Mosaic of uses	21	Not used	Not used
Non vegetated area	22	Not used	Not used
Beach, dune and sand spot	23	Not used	Not used
Urban area	24	Urban area	Not used
Other non-vegetated areas	25	Not used	Not used

Land use and land cover class	MapBiomas class code	Grouped land cover classes	Vegetation class
Rocky outcrop	29	Not used	Not used
Mining	30	Mining	Not used
Aquaculture	31	Not used	Not used
Salt flat	32	Natural vegetation	Natural Vegetation
River, lake and ocean	33	Water	Not used
Perennial crop	36	Perennial crop	Not used
Soybean	39	Temporary crop	Not used
Rice	40	Temporary crop	Not used
Other temporary crops	41	Temporary crop	Not used
Coffee	46	Perennial crop	Not used
Citrus	47	Perennial crop	Not used
Other perennial crops	48	Perennial crop	Not used
Wooded sandbank vegetation	49	Forest vegetation	Forest Vegetation and Natural Vegetation
Herbaceous sandbank vegetation	50	Natural vegetation	Natural Vegetation
Cotton	62	Temporary crop	Not used

We used linear infrastructure (roads and railways) to trim Forest Vegetation and Natural Vegetation areas overlapping with these structures. Thus, we avoided overestimating large fragments, since roads can decrease the connectivity of large patches (Martinez Pardo et al. 2023) for different taxa (Cassimiro et al. 2023). Road and railway data were downloaded

from official geospatial databases for the three countries: Brazil (Instituto Brasileiro de Geografia e Estatística – IBGE; IBGE, 2021; https://www.ibge.gov.br), Argentina (Instituto Geográfico Nacional – IGN; IGN, 2022; https://www.ign.gob.ar) and Paraguay (Instituto Nacional de Estadística – INE; INE, 2022; https://www.ine.gov.py). The data summed 14,072 km of railways and 125,483 km of roads, totaling 139,554 km (Figure 4). We did not find official railway data for Paraguay, so this effect may be underestimated for this country. For Brazil, we selected paved, operational and constructed roads, and railways selected by their relative surface position and train section for 2021. For Argentina, we considered national and provincial paved roads for the year 2021. For Paraguay, we only considered the main roads for the year 2012, without making a distinction regarding the paving of roads, since this information was not available.

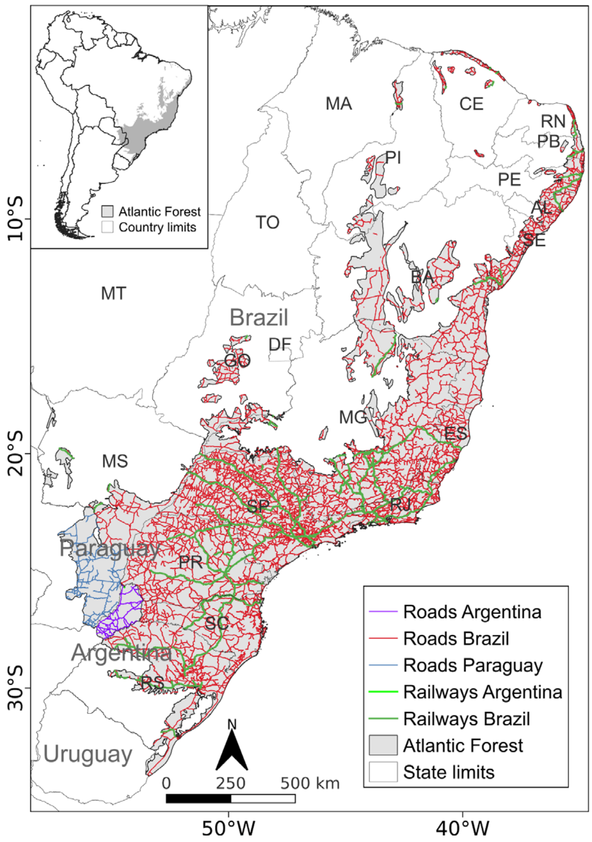

Figure 4. Linear infrastructure (roads and railways) network used to trim the forest vegetation and native vegetation fragments within the Atlantic Forest. Abbreviations correspond to Brazilian states presented in Figure 2.

The road and railway layers were rasterized using a parameter that creates densified lines, i.e., all cells touched by the line were included as data for rasterization, resulting in densified lines. This guaranteed that the roads and railways would trim the fragments. After the lines were rasterized, the roads covered 528,983 ha (0.33% from the Atlantic Forest delimitation). We trimmed the fragments of vegetation using the rasterized data generated (Vancine et al. 2024).

Urban areas for Brazil were selected from MapBiomas. For Argentina, this dataset was downloaded from Instituto Geográfico Nacional (Instituto Geográfico Nacional – IGN, https://www.ign.gob.ar) and for Paraguay from Instituto Nacional de Estadística (Instituto Nacional de Estadística – INE, https://www.ine.gov.py) (Figure 5), and covered 2,401,850 ha (1.48% from the Atlantic Forest limit).

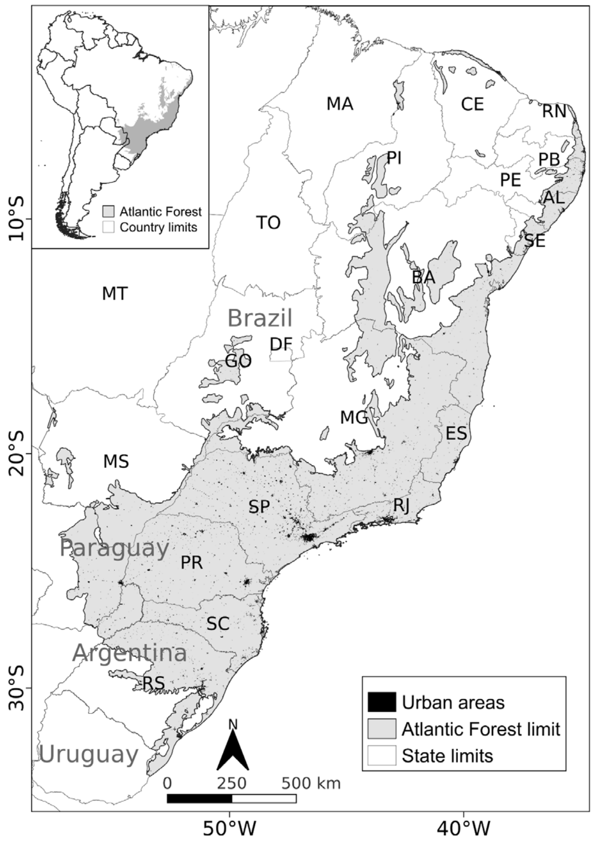

Figure 5. Urban areas within the Atlantic Forest. Abbreviations correspond to Brazilian states presented in Figure 2.

Protected areas, indigenous territories, and quilombola territories

The limits of the Protected Areas were downloaded from Protected Planet portal (UNEP-WCMC and IUCN, 2022, www.protectedplanet.net) for the IUCN categories of protected areas (“Ia”, “Ib”, “II”, “III” and “IV”), which comprises 986 reserves (4,620,245 ha; 2.84% from the Atlantic Forest limit) (Figure 6a). These IUCN categories encompass the following protection categories of Argentina (Municipal Nature Park, National Park, Nature Monument, Private Refuge, Private Wildlife Refuge, Provincial Park, Strict Nature Reserve, Wilderness Nature Reserve and Wildlife Reserve), Brazil (Area of Relevant Ecological Interest, Biological Reserve, Ecological Station, Natural Heritage Private Reserve, Natural Monument, Park, Ramsar Site, Wetland of International Importance, Wildlife Refuge), and Paraguay (National Park, Natural Private Reserve, Natural Reserve, Scientific Monument and Scientific Reserve).

Indigenous territories are lands traditionally occupied by indigenous communities and ethnic groups, defined as those inhabited by them on a permanent basis; used for their productive activities; essential to the preservation of the environmental resources necessary for their well-being; and necessary for their physical and cultural reproduction, being their uses, customs, and traditions (Benzeev et al. 2023). Indigenous territories were downloaded from Fundação Nacional dos Povos Indígenas (Fundação Nacional dos Povos Indígenas, 2020, https://www.gov.br/funai/pt-br) for Brazil, selecting only “Homologated”. For Paraguay, data were downloaded from Tierras Indígenas (Tierras Indígenas, 2022, https://www.tierrasindigenas.org.py). Although we know that there are indigenous territories for the Misiones region in Argentina, after consulting the official data from the Argentine government (https://www.argentina.gob.ar/interior/inai), we realized that these data was not available in vector format to our knowledge, so we did not consider them. Indigenous territories data included 1023 territories (1,324,973 ha; 0.81% from the Atlantic Forest limit) (Figure 6b).

Quilombola territories are delimited areas where quilombola communities live. Quilombola ethnic groups are predominantly constituted by rural or urban black population, who define themselves based on specific relationships with the land, kinship, territory, ancestry, traditions, and their own cultural practices (Leite 2015). According to the Brazilian Constitution, “For the purposes of this Decree, the term ‘remnants of quilombo communities’ refers to ethno-racial groups, according to self-identification criteria, with their own historical trajectory, specific territorial relations, and a presumption of Black ancestry related to

resistance against historical oppression” (Article 2 of Decree 4.887/2003, https://www.planalto.gov.br/ccivil_03/decreto/2003/d4887.htm). Furthermore, following the definition of Instituto Nacional de Colonização e Reforma Agrária (INCRA): “Quilombola communities are ethnic groups – predominantly made up of rural or urban Black populations – that define themselves based on specific relationships with the land, kinship, territory, ancestry, traditions, and their own cultural practices” (https://www.gov.br/incra/pt-br/assuntos/governanca-fundiaria/quilombolas). Quilombola territories were included for Brazil and only included the quilombola territories officially regularized up to date (2020). For Argentina and Paraguay, after searching the official websites of these governments and NGOs, we found no such data available, despite these countries having populations of African descent. The limits of the quilombola territories were downloaded from Instituto Nacional de Colonização e Reforma Agrária (INCRA) (https://certificacao.incra.gov.br/csv_shp/export_shp.py), which comprises 157 territories (486,533 ha; 0.30% from the Atlantic Forest limit) (Figure 6c).

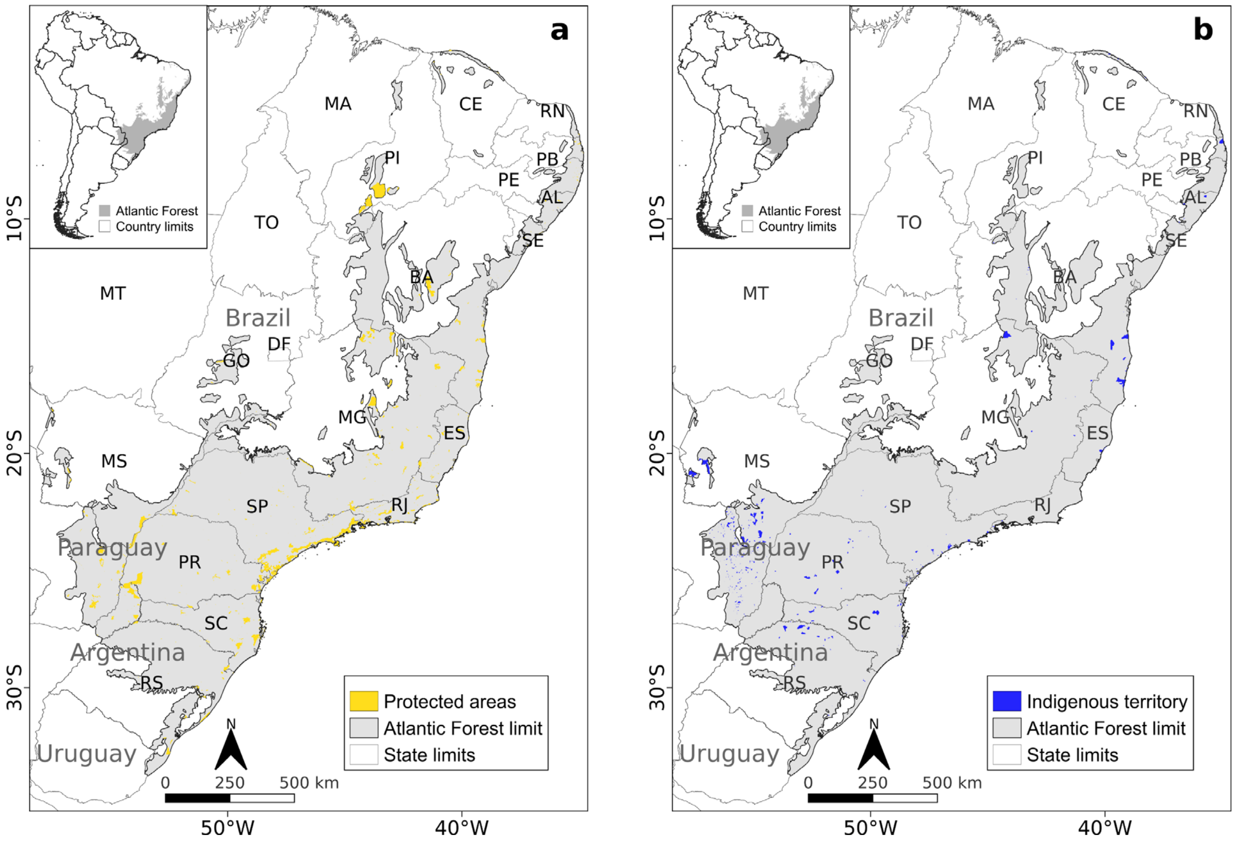

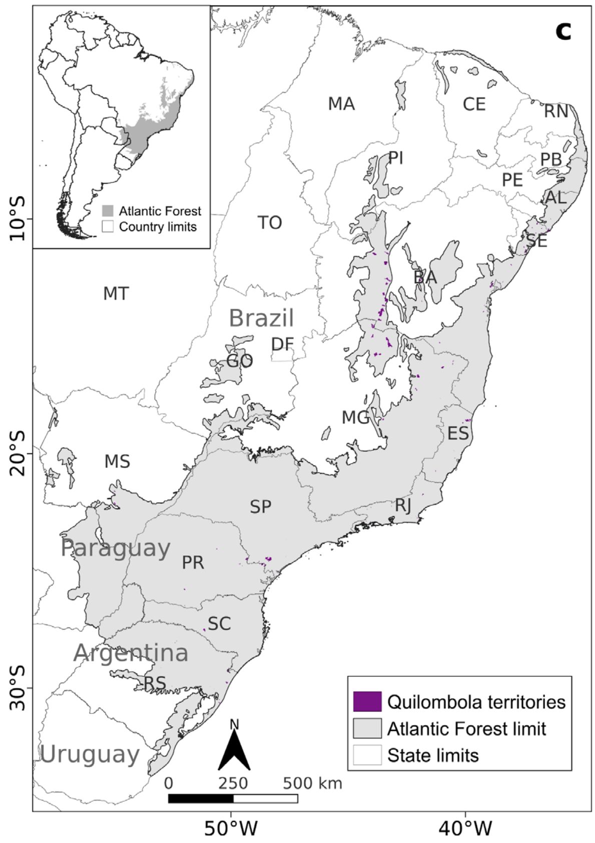

Figure 6. Protected areas (a), indigenous territories (b), and quilombola territories within the Atlantic Forest. Abbreviations correspond to Brazilian states presented in Figure 2.

Topography data

Topographic metrics were calculated from FABDEM v1.2 (forest and buildings removed Copernicus DEM), an elevation raster map that used machine learning to remove buildings and tree height biases from the Copernicus GLO 30 Digital Elevation Model (DEM) (Hawker et al. 2022) (Figure 7).

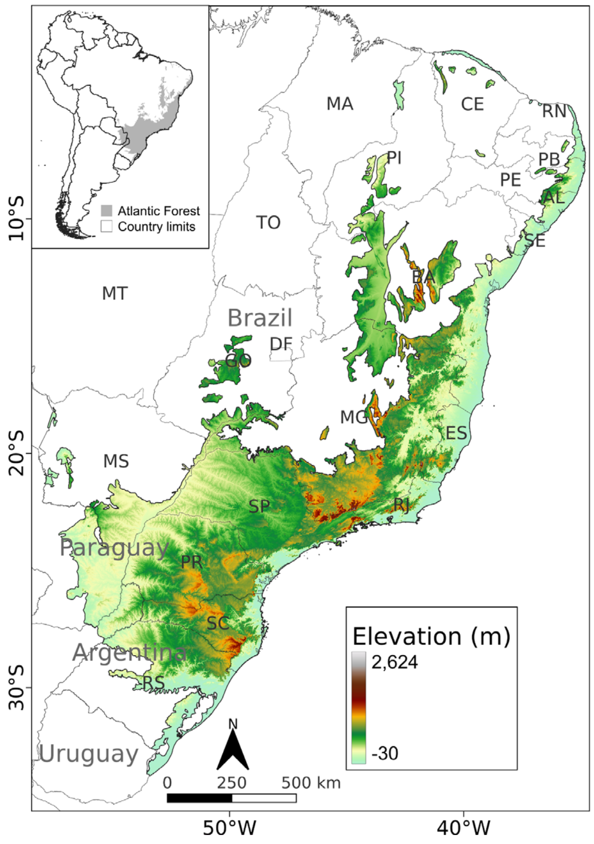

Figure 7. Elevation (meters above sea level) from FABDEM v1.2 across the Atlantic Forest. Abbreviations correspond to Brazilian states presented in Figure 2.

Software

All the landscape, topographic, hydrological, and anthropogenic metrics were processed using GRASS GIS 8.3 (Neteler et al. 2012) and R language 4.3 (R Core Team, 2023) with the aid of the *rgrass* package (Bivand, 2023). GRASS GIS (Geographical Resources Analysis Support System) is a free and open-source Geographic Information System (GIS), created around 1985, and in continuous development. It provides over 400 well-documented and peer-reviewed modules for spatial analysis, modeling, and visualization, and is widely used in academia, business, and public administration. Developed by a global community, GRASS GIS runs natively on major operating systems and is particularly recognized for its applications in environmental modeling. Its architecture is optimized for handling and processing large volumes of geospatial data, making it particularly suitable for complex environmental modeling and high-performance geocomputation (Neteler et al. 2012). All landscape metrics were calculated using custom functions based on *LSMetrics* and translated to R (https://doi.org/10.5281/zenodo.3736443; Niebuhr et al. 2020).

All codes used to calculate the metrics are available on Zenodo (Vancine and Niebuhr 2025; https://doi.org/10.5281/zenodo.14814102). These scripts represent the step-by-step process for calculating the metrics, allowing the process to be completely reproducible. For example, the script "01_01_download_limits.R" downloads the Atlantic Forest boundary, and "01_02_download_landscape.R" downloads the land use and land cover layers from MapBiomas using an integration with Google Earth Engine. Likewise, the other scripts download the other input sources of data, describe their process of import into GRASS GIS, and the computation of the different types of metrics presented in this dataset. By making these scripts available, we believe that this approach to calculating these metrics can be replicated for other biomes or regions of the world, or for other timestamps using the available data for the Atlantic Forest. However, some steps were omitted or need to be performed in addition to the scripts for the full analysis to be performed. For example, the final boundary of the Atlantic Forest was manually edited and cannot be reproduced using scripts (but a detailed description is available in Vancine et al. 2024). Another example that users wishing to reproduce the analyses should be aware of is that we used GNU/Linux to calculate the metrics. GRASS GIS works integrated with R through *rgrass* R package, and to do so, you need to specify the GRASS GIS directory, which on GNU/Linux can be accessed as follows: `system("grass --config path", inter = TRUE)`. On Microsoft Windows®, it is needed to specify a different path, for example: `"C:/Program Files/GRASS GIS 8.3"`. This can be a bit confusing for new users of these software integrations.

Landscape metrics

We calculated 39 landscape metrics of nine types (Table 3) based on the habitat map (binary habitat/non-habitat map, Figure 3c) of Forest Vegetation and Natural Vegetation, and the multi-class map (31 classes, Figure 3a). The values of the landscape metrics were spatialized (mapped onto) to the cells. The metrics derived from Forest Vegetation and Natural Vegetation were based on data trimmed by linear structure (roads and railways) (Vancine et al. 2024). Although it is common to consider fragment and patch as synonyms, here we follow the classic landscape model (patch-corridor-matrix) to differentiate them (Forman and Godron 1986). Thus, a "fragment" represents a grouping of all contiguous pixels (considering the 8 neighboring cells), while a "patch" represents a grouping of pixels (considering the 8 neighboring cells), disregarding portions of pixels that form corridors and/or branches.

Here, to exemplify the method used for calculating landscape metrics, we display two toy landscapes (Fletcher and Fortin 2018): a binary raster (Figure 8A) and a multi-class raster (Figure 8B). The toy landscapes have a resolution of 30 m, the same resolution of the ATLANTIC SPATIAL dataset, chosen to make it easier to understand metrics. Thus, each pixel has 900 m^2 and the distance between pixel centers is 30 m.

Toy landscapes (Figure 8): two raster layers (16×16 cells with a spatial resolution of 30 m). Cells of the toy landscape (binary) were filled with 0 and 1 values, where 0 represents not-habitat and 1 represents habitat (a. Toy landscape (binary)). For the toy landscape (multi-class), cells were filled with values from 0 to 5, where each value represents a different LULC class (b. Toy landscape (multi-class)).

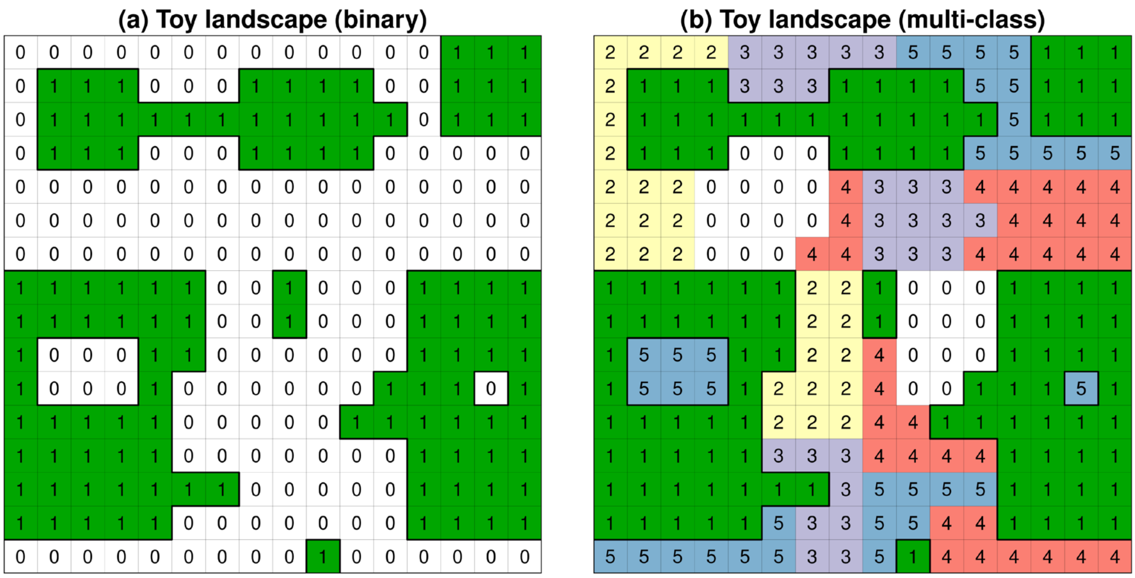

Figure 8. Toy landscapes. A binary toy landscape (a); a multi-class toy landscape (b).

Table 3. Landscape metrics used and their description. All metrics were calculated for a spatial resolution of 30 m. Edge depth is the minimum distance at which cells are classified as edges, those that are further away are classified as cores. Gap-crossing considers the ability of an organism to cross non-habitat gaps, characterizing the distance to functional connectivity. Scale is measured by the radius of the buffer for which the moving window is used to impute the "scale of effect" for different organisms' responses on landscape metrics. The term "original" in "Patch area original", "Core area original", and "Edge area original" and other metrics represents the original assignment of landscape metric values to the original fragments. Some metrics, such as core or edge, are calculated considering only these pixels, distorting the original fragment pixel shape. Thus, "original" represents the assignment of these metrics to the pixels of the original fragments.

Metric	Metric type	Short description	Values	Edge depth (in meters)	Gap-crossing (in meters)	Scale (buffer radius in meters)	Reference
1. Fragment ID	Fragment	Fragment identification (cells clumped in its vicinity, considering the 8 neighboring cells)	Units	NA	NA	NA	McGarigal et al. (2023)
2. Fragment area	Fragment	Fragment area (sum of the area of all cells belonging to each fragment ID)	Hectares	NA	NA	NA	McGarigal et al. (2023)

Metric	Metric type	Short description	Values	Edge depth (in meters)	Gap-crossing (in meters)	Scale (buffer radius in meters)	Reference
3. Percentage of fragment	Fragment	Percentage of fragment in the vicinity (average neighborhood values for different buffer sizes)	0 to 100%	NA	NA	50, 100, 150, 200, 250, 500, 750, 1000, 1500, 2000, 2500, 5000, 7500, 10000	McGarigal et al. (2023)
4. Patch ID	Patch	Patch identification (cells clumped in its vicinity, considering the 8 neighboring cells), discarding branches and corridors	Units	30	NA	NA	McGarigal et al. (2023)
5. Patch area	Patch	Patch area (sum of the area of all cells belonging to each patch ID), discarding branches and corridors	Hectares	30	NA	NA	McGarigal et al. (2023)

Metric	Metric type	Short description	Values	Edge depth (in meters)	Gap-crossing (in meters)	Scale (buffer radius in meters)	Reference
6. Patch area original	Patch	Patch area assigned to the original fragment. Here each fragment cell is assigned the value of the sum of the areas of all patches contained in the fragment	Hectares	30	NA	NA	McGarigal et al. (2023)
7. Number of patches	Patch	Number of patches (number of patch IDs within a fragment) assigned to the original fragment. Here each cell of a fragment is assigned the value number of patches contained in the fragment	Units	30	NA	NA	McGarigal et al. (2023)

Metric	Metric type	Short description	Values	Edge depth (in meters)	Gap-crossing (in meters)	Scale (buffer radius in meters)	Reference
8. Landscape morphology	Landscape morphology	Identifies landscape morphologies: matrix (0), core (1), edge (2), corridor (3), branch (4), stepping stone (5), and perforation (6)	0 to 6	30	NA	NA	Vogt et al. (2009)
9. Matrix	Landscape morphology	Identify matrix (non-habitat cells = 1)	0 and 1	30	NA	NA	Vogt et al. (2009)
10. Core	Landscape morphology	Identify fragment cores (core cells = 1)	0 and 1	30	NA	NA	et al. Vogt et al. (2009)
11. Edge	Landscape morphology	Identify fragment edges (external edge cells = 1)	0 and 1	30	NA	NA	Vogt et al. (2009)
12. Corridor	Landscape morphology	Identify corridors (linear elements that connect core	0 and 1	30	NA	NA	Vogt et al. (2009)

Metric	Metric type	Short description	Values	Edge depth (in meters)	Gap-crossing (in meters)	Scale (buffer radius in meters)	Reference
		cells = 1)					
13. Branch	Landscape morphology	Identify branches (linear elements that do not connect core cells = 1)	0 and 1	30	NA	NA	Vogt et al. (2009)
14. Stepping stone	Landscape morphology	Identify stepping stones (isolated small elements without core cells = 1)	0 and 1	30	NA	NA	Vogt et al. (2009)
15. Perforation	Landscape morphology	Identify perforations (edge that composes the internal edge of a fragment = 1)	0 and 1	30	NA	NA	Vogt et al. (2009)
16. Core	Core and edge	Identify core cells (core = 1)	0 and 1	30, 60, 90, 120, 240	NA	NA	McGarigal et al. (2023)
17. Core ID	Core and edge	Core identification (core cells	Units	30, 60, 90, 120, 240	NA	NA	McGarigal et al. (2023)

Metric	Metric type	Short description	Values	Edge depth (in meters)	Gap-crossing (in meters)	Scale (buffer radius in meters)	Reference
		clumped in its vicinity, considering the 8 neighboring cells)					
18. Core area	Core and edge	Core area (sum of the area of all core cells belonging to that core ID)	Hectares	30, 60, 90, 120, 240	NA	NA	McGarigal et al. (2023)
19. Core area original	Core and edge	Core area assigned to the original fragment. Here each cell of a fragment is assigned the value total area of all cores contained in the fragment	Hectares	30, 60, 90, 120, 240	NA	NA	McGarigal et al. (2023)
20. Number of cores	Core and edge	Number of cores within a fragment. Here each cell of a	Units	30, 60, 90, 120, 240	NA	NA	McGarigal et al. (2023)

Metric	Metric type	Short description	Values	Edge depth (in meters)	Gap-crossing (in meters)	Scale (buffer radius in meters)	Reference
		fragment is assigned the value number of cores contained in the fragment					
21. Edge	Core and edge	Identify edge cells (edge = 1). This includes both external and internal edges (perforations)	0 and 1	30, 60, 90, 120, 240	NA	NA	McGarigal et al. (2023)
22. Edge ID	Core and edge	Edge identification (cells clumped in its vicinity, considering the 8 neighboring cells)	Units	30, 60, 90, 120, 240	NA	NA	McGarigal et al. (2023)
23. Edge area	Core and edge	Edge area (sum of the area of all edge cells belonging to that edge ID)	Hectares	30, 60, 90, 120, 240	NA	NA	McGarigal et al. (2023)
24. Edge area	Core and edge	Edge area	Hectares	30, 60, 90, 120,	NA	NA	McGarigal et al.

Metric	Metric type	Short description	Values	Edge depth (in meters)	Gap-crossing (in meters)	Scale (buffer radius in meters)	Reference
original		assigned to the original fragment. Here each cell of a fragment is assigned to the value total area edge in the fragment		240			(2023)
25. Percentage of core	Core and edge	Percentage of core cells within the vicinity (average neighborhood values for different buffer sizes)	0 to 100%	30, 60, 90, 120, 240	NA	50, 100, 150, 200, 250, 500, 750, 1000, 1500, 2000, 2500	McGarigal et al. (2023)
26. Percentage of edges	Core and edge	Percentage of edge cells in the vicinity (average neighborhood values for different buffer sizes)	0 to 100%	30, 60, 90, 120, 240	NA	50, 100, 150, 200, 250, 500, 750, 1000, 1500, 2000, 2500	McGarigal et al. (2023)

Metric	Metric type	Short description	Values	Edge depth (in meters)	Gap-crossing (in meters)	Scale (buffer radius in meters)	Reference
27. Perimeter	Perimeter	Perimeter (number of cells sides of a fragment facing the matrix, including any internal holes)	Meters	30	NA	NA	McGarigal et al. (2023)
28. Perimeter-area ratio	Perimeter	Perimeter-area ratio (ratio between fragment perimeter and fragment area)	0 to infinity	30	NA	NA	McGarigal et al. (2023)
29. Distance inside	Distance	Euclidean distance to the nearest fragment edge cell, inside the fragment	Meters	NA	NA	NA	Ribeiro et al. (2009)
30. Distance outside	Distance	Euclidean distance to the nearest fragment edge cell, outside the fragment	Meters	NA	NA	NA	Ribeiro et al. (2009)
31. Distance	Distance	Euclidean	Meters	NA	NA	NA	Ribeiro et al.

Metric	Metric type	Short description	Values	Edge depth (in meters)	Gap-crossing (in meters)	Scale (buffer radius in meters)	Reference
		distance to the nearest fragment edge cell, both inside (negative) and outside (positive) the fragment					(2009)
32. Structural connectivity	Structural connectivity	Structural connectivity (represents the area of habitat structurally connected to a patch, considering corridors, branches, and possibly other patches, but disregarding the area of the own patch)	Hectares	30	NA	NA	Ribeiro et al. (2009)
33. Structurally connected area	Structural connectivity	Structurally connected area (calculated from the original	Hectares	30	NA	NA	Ribeiro et al. (2009)

Metric	Metric type	Short description	Values	Edge depth (in meters)	Gap-crossing (in meters)	Scale (buffer radius in meters)	Reference
		fragment, where the structural connectivity of the patches was associated with the fragment)					
34. Functionally connected dilation	Functional connectivity	Functionally connected dilation (fragments dilate by half the value of the organism's gap-crossing capacity)	Hectares	NA	60, 120, 180, 240, 300, 600	NA	Ribeiro et al. (2009)
35. Functionally connected ID	Functional connectivity	Functionally connected identification (fragments that are at the shortest distance from the gap-crossing are grouped, receiving the same ID)	Hectares	NA	60, 120, 180, 240, 300, 600	NA	Ribeiro et al. (2009)

Metric	Metric type	Short description	Values	Edge depth (in meters)	Gap-crossing (in meters)	Scale (buffer radius in meters)	Reference
36. Functionally connected area	Functional connectivity	Functionally connected area (area of these fragments with the same ID was summed)	Hectares	NA	60, 120, 180, 240, 300, 600	NA	Ribeiro et al. (2009)
37. Functional connectivity	Functional connectivity	Functional connectivity (difference between the functionally connected area and the fragment size)	Hectares	NA	60, 120, 180, 240, 300, 600	NA	Ribeiro et al. (2009)
38. Landscape Shannon diversity	Landscape diversity	Landscape Shannon diversity (consider the number of classes in each class cell within the moving window of analysis for the Shannon index)	0 to infinity	NA	NA	50, 100, 150, 200, 250, 500, 750, 1000, 1500, 2000	Rocchini et al. (2013)

Metric	Metric type	Short description	Values	Edge depth (in meters)	Gap-crossing (in meters)	Scale (buffer radius in meters)	Reference
39. Landscape Simpson diversity	Landscape diversity	Landscape Simpson diversity (consider the number of classes in each class cell within the moving window of analysis for the Shannon index)	0 to 1	NA	NA	50, 100, 150, 200, 250, 500, 750, 1000, 1500, 2000	Rocchini et al. (2013)

Fragment area metrics (Figure 9): considering a binary habitat map, all cells of habitat were clumped with other cells of habitat in its vicinity (considering the 8 neighboring cells). Each clump of habitat was called a fragment and was given an ID (**Metric 1: Fragment ID**). For each fragment ID, its area (**Metric 2: Fragment area**) was calculated as the sum of the area of all cells belonging to that fragment ID. The unit used to calculate the area is hectares. Non-habitat cells are returned as NULL values.

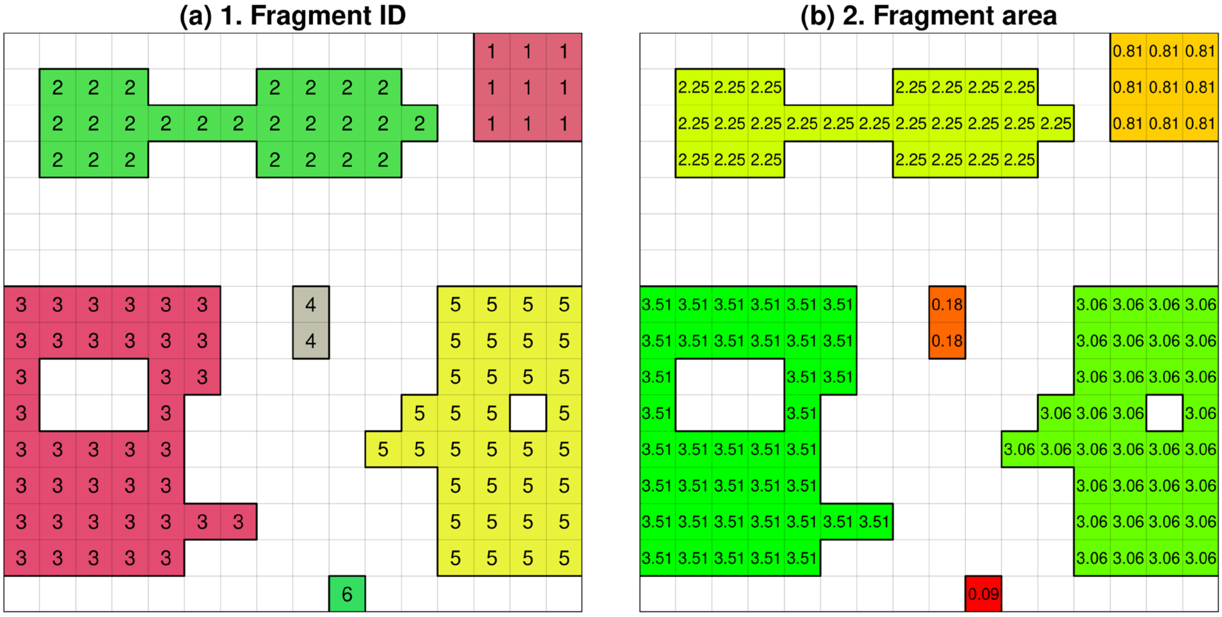

Figure 9. Fragment area metrics. Metric 1: Fragment ID is the fragment identification (a). Metric 2: Fragment area is the fragment area calculated in hectares (b).

Percentage of fragments metric (Figure 10): considering a binary habitat map, each cell of the map presented a value of the percentage of fragments within a circular moving window with a given size, centered in the focal cell (amount of habitat cells/total number of cells on the moving window). It varies between 0% and 100% (**Metric 3: Percentage of fragments**). Buffer radius represented half the size of a circular moving window, e.g., for a buffer size of 50 m, the moving window size was 100 m. Buffer radii used: 50 m, 100 m, 150 m, 200 m, 250 m, 500 m, 750 m, 1,000 m, 1,500 m, 2,000 m, 2,500 m, 5,000 m, 7,500 m, 10,000 m. The file names have the buffer radius value.

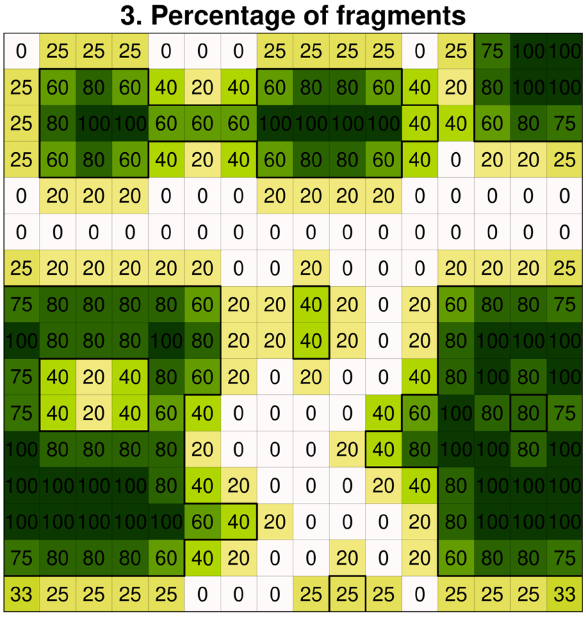

Figure 10. Percentage of fragments metric. Metric 3: percentage of area covered by fragments considering a buffer radius of 100 m (i.e., a circular moving window with a diameter of 200 m), illustrated in the toy landscape.

Patch area metrics (Figure 11): considering a binary habitat map, patch metrics are like fragment area metrics, but they discard habitat branches and corridors. The result is a map of clusters (considering the 8 neighboring cells) of cells, which does not consider corridors or branches. Each habitat cluster was called a patch and given an ID (**Metric 4: Patch ID**). For each patch ID, its area (**Metric 5: Patch area**) was calculated as the sum of the area of all cells belonging to that patch ID. The patch area was attributed to the original fragment ID, which sums the area of all patches belonging to the same fragment (**Metric 6: Patch area original**). The number of different patch IDs for a fragment was also calculated (**Metric 7: Number of patches**). Edge depths considered: 30 m. The unit used to calculate the area was hectares. Non-habitat cells were assigned with NULL values.

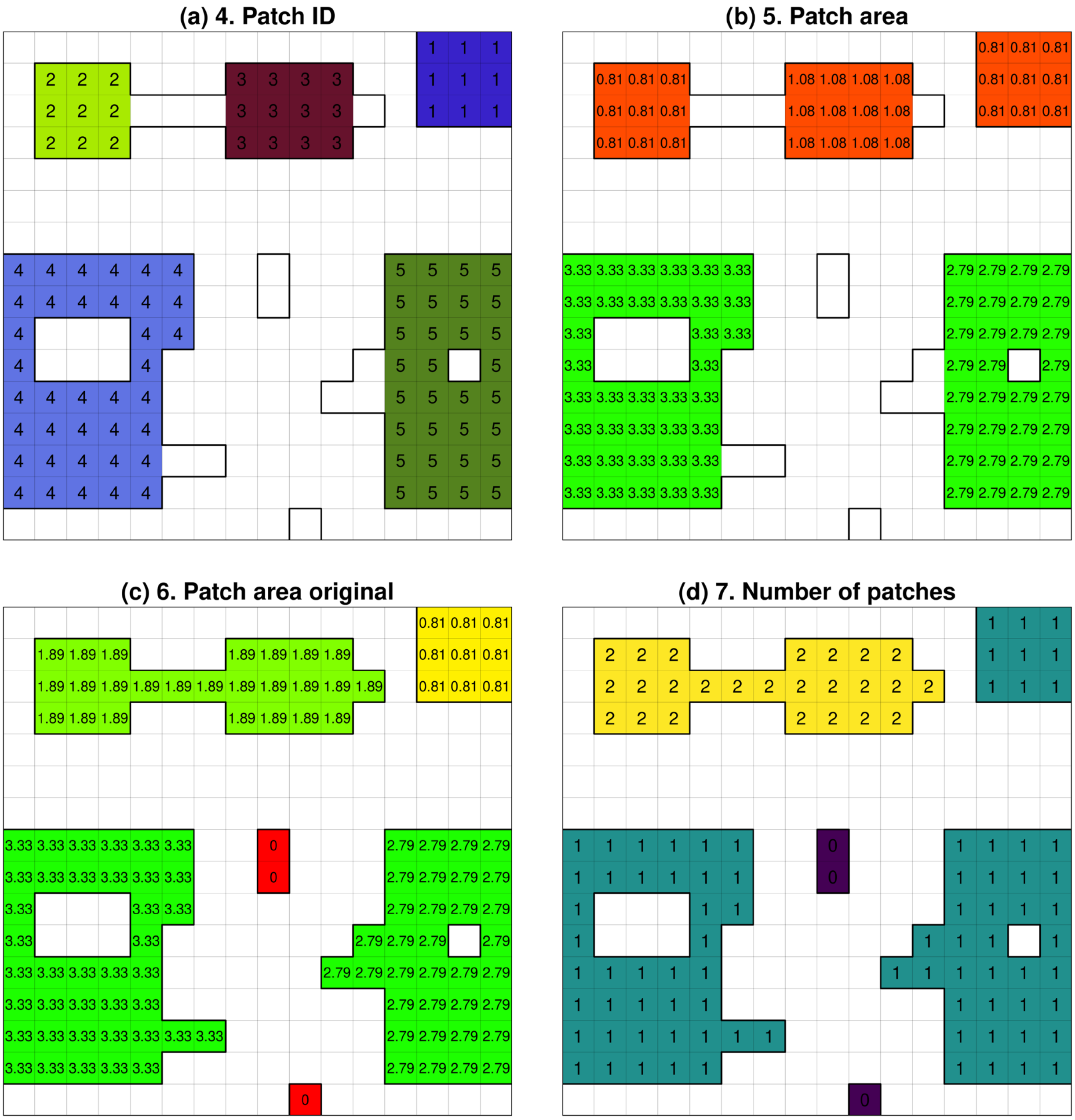

Figure 11. Patch area metrics. Metric 4: Patch ID is the patch identification (a). Metric 5: Patch area is the patch area in hectares (b). Metric 6: Patch area original is the patch area for the original fragment in hectares (c). Metric 7: Number of patches is the number of patches for the original fragment (d). The edge depth was set as 30 m in this example.

4. Landscape morphology metrics (Figure 12): considering a binary habitat map, these metrics classify the landscape as a set of morphological/structural categories (**Metric 8: Landscape morphology**), i.e., whether a cell is matrix, core, edge, corridor, branch, stepping stone or perforation. This classification is made by considering an edge depth of 30 m to distinguish the edge and the core of a habitat fragment. Matrices are the non-habitat cells from the binary map (**Metric 9: Landscape morphology matrix**). Cores are habitat cells after removing the edge cells (**Metric 10: Landscape**

morphology core). Edges are habitat cells that are closer to the edge than the chosen edge depth, but are not corridors, branches, stepping stones or perforations (**Metric 11: Landscape morphology edge**). Corridors are edge cells that connect two or more core cells (**Metric 12: Landscape morphology corridor**). Branches are edge cells that do not connect cores (**Metric 13: Landscape morphology branch**). Stepping stones are edge cells that do not have core cells inside them (**Metric 14: Landscape morphology stepping stone**). Perforations are edge cells that compose the internal edge of a fragment (**Metric 15: Landscape morphology perforation**).

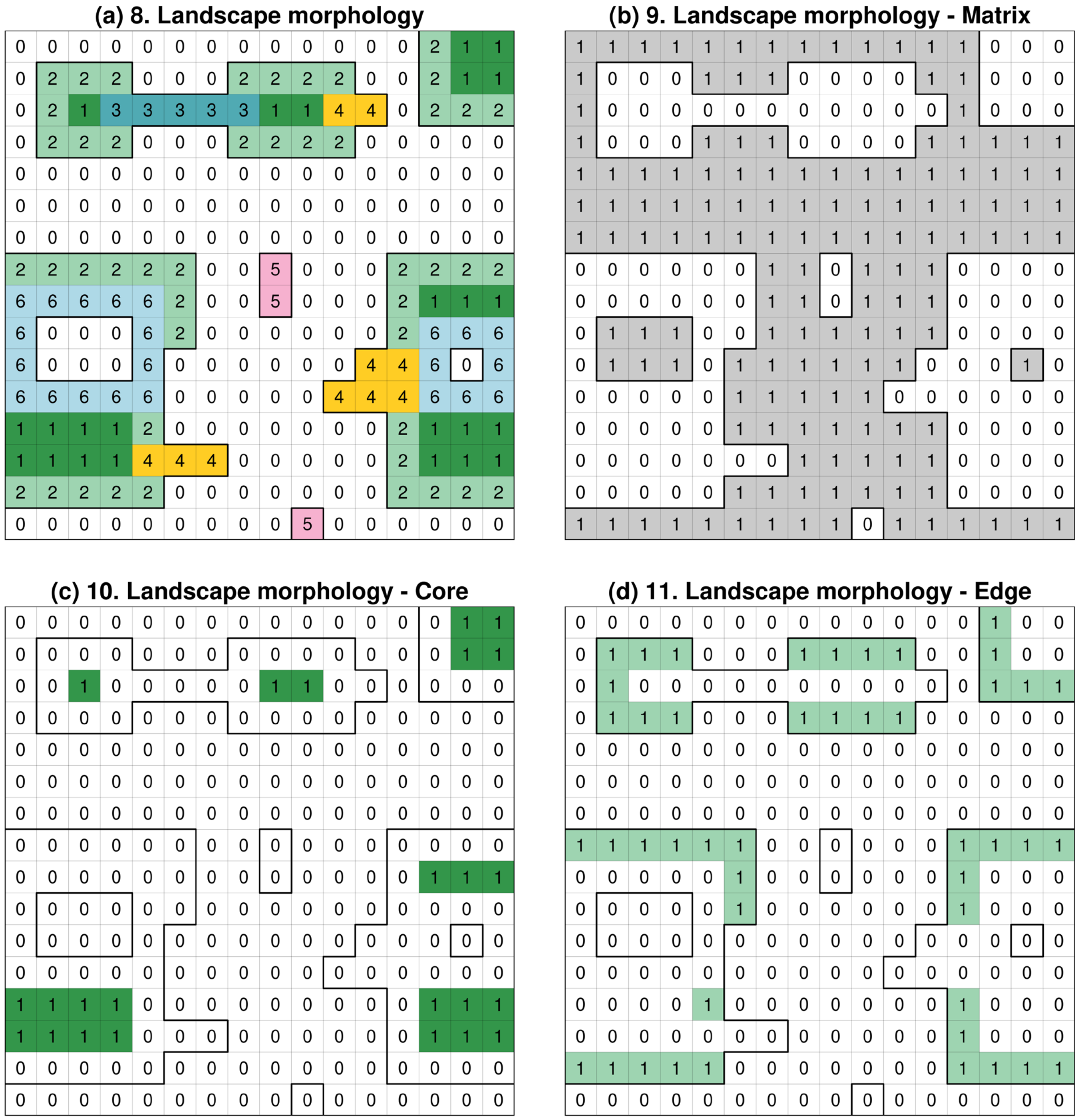

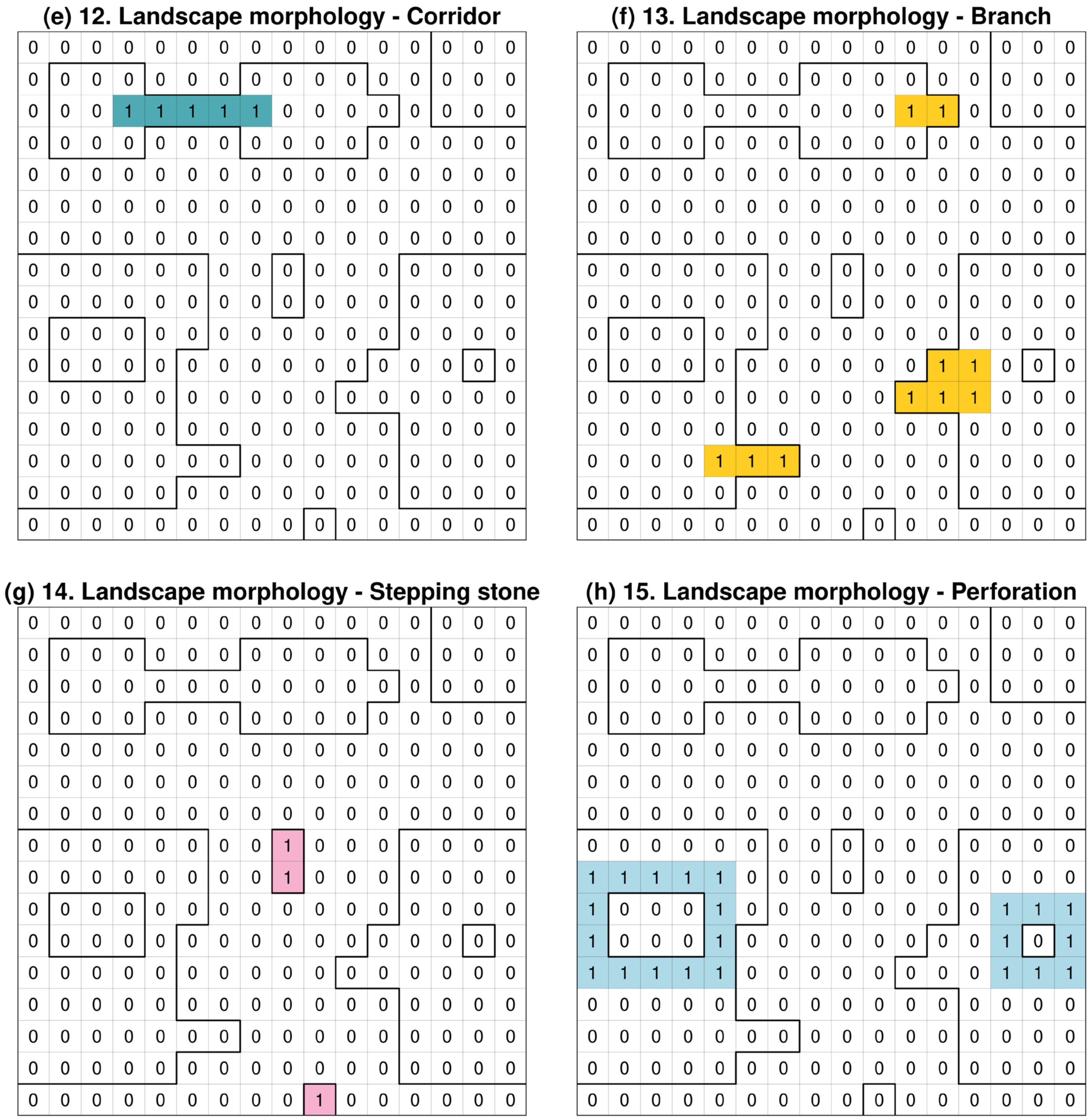

Figure 12. Landscape morphology metrics. Metric 8: Landscape morphologies: matrix (0), core (1), edge (2), corridor (3), branch (4), stepping stone (5), and perforation (6) (a). Metric 9: matrix = non-habitat cells (b). Metric 10: Core = habitat cells, removing the edge cells (c). Metric 11: Edge =habitat cells that are closer to the habitat edge than the chosen edge depth (d). Metric 12: Corridor = edge cells that connect two or more core cells (e). Metric 13: Branch = edge cells that do not connect cores (f). Metric 14: Stepping stone = edge cells that do not have core cells (g). Metric 15: Perforation = edge cells that compose the internal edge of a fragment (h). For details, see Vogt et al. (2009). The edge depth used in this illustrative figure was 30 m.

Core and edge area metrics (Figure 13): considering a binary habitat map, these metrics classify cells in core or edge, considering the depth of edge. Cells that are closer (or at the same distance)

from the edge than the edge depth are classified as edges, those that are further away inside the habitat patches are classified as core. We clumped the core and edge cells (considering the 8 neighboring cells; **Metric 16: Core** and **Metric 21: Edge**) and gave it an ID (**Metric 17: Core ID** and **Metric 22: Edge ID**). For each core and edge ID, its area was calculated as the sum of the area of all cells belonging to that core or edge ID (**Metric 18: Core area** and **Metric 23: Edge area**). We also calculated the area of a core or edge of the original fragment by summing the area of the core or edge cells belonging to a fragment (**Metric 19: Core area original** and **Metric 24: Edge area original**), and the number of cores (**Metric 20: Number of cores**), which was the number of different cores IDs for a fragment. The idea behind calculating original metrics is that edge and interior information is summarized and/or assigned to the original fragments, so that the original landscape structure can be used in spatial predictions, for example. Notice that Metric 21: Edge differs from the morphological classification of edges (**Metric 11: Landscape morphology edge**) because the latter subdivides edge cells into edges, corridors, branches, stepping stones, and perforations. Metric 21 (Edge) includes all these in a single category. Edge depths considered: 30 m, 60 m, 120 m, 240 m. Non-habitat cells were assigned with NULL values, except for core and edge binary maps.

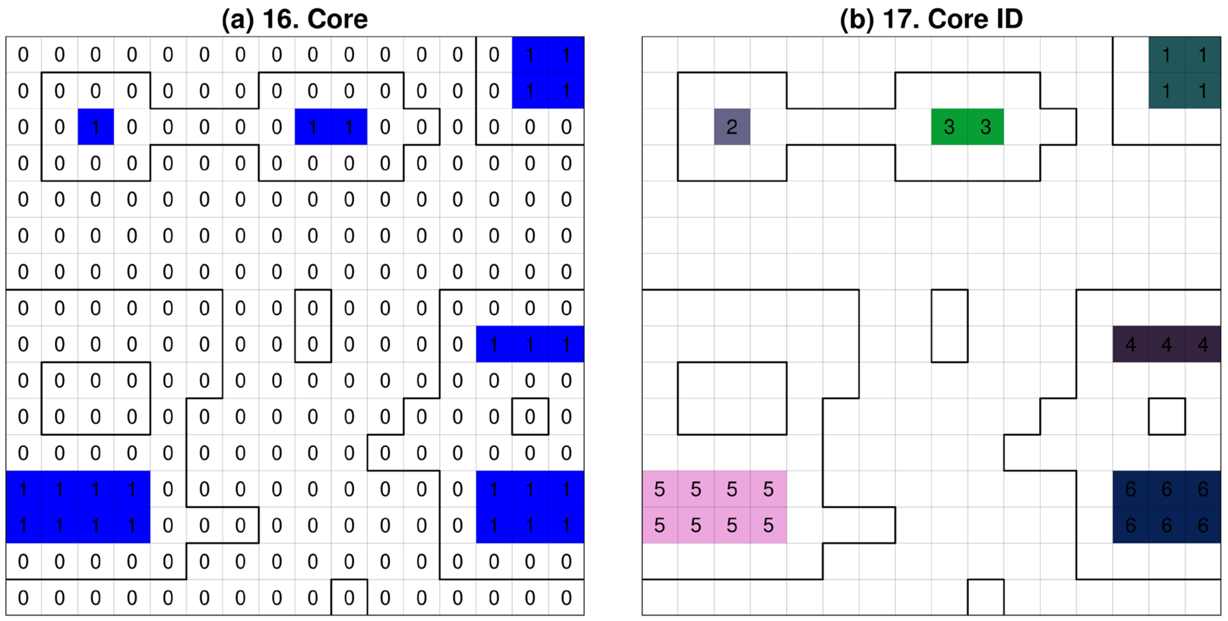

(c) 18. Core area

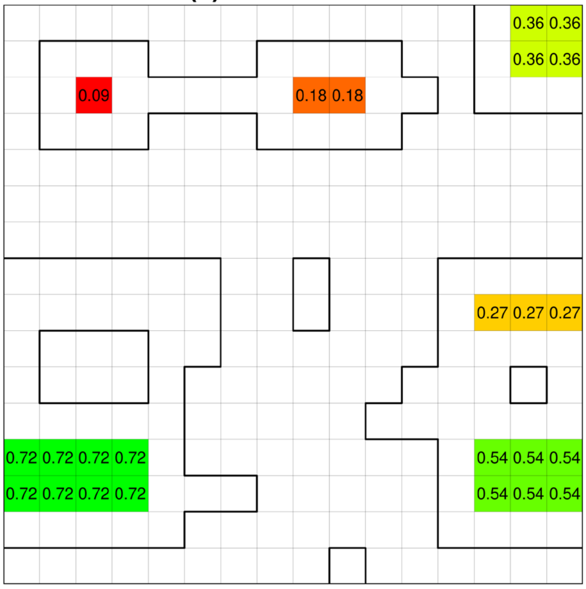

(d) 19. Core area original

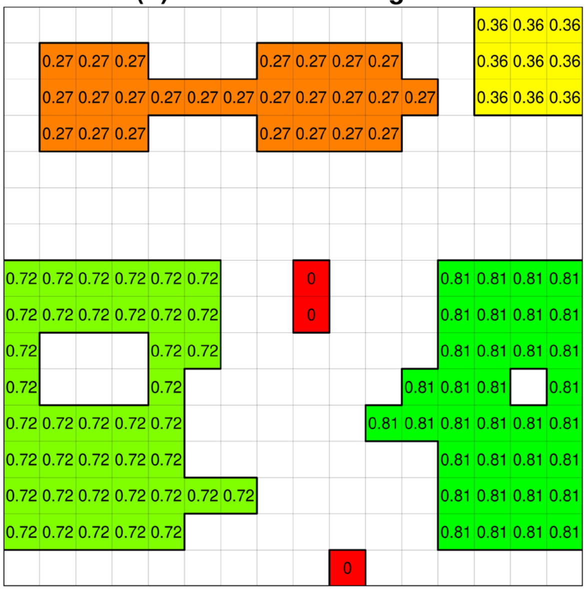

(e) 20. Number of cores

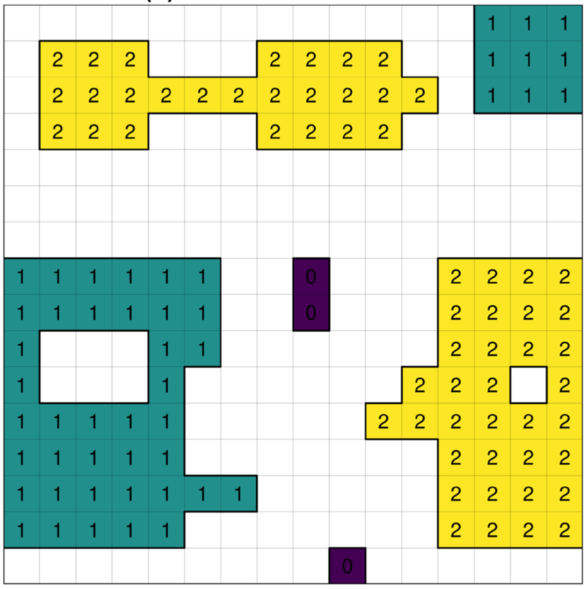

(f) 21. Edge

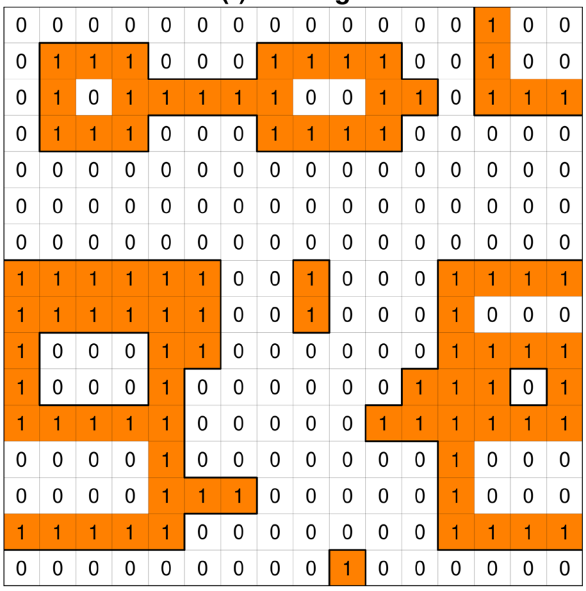

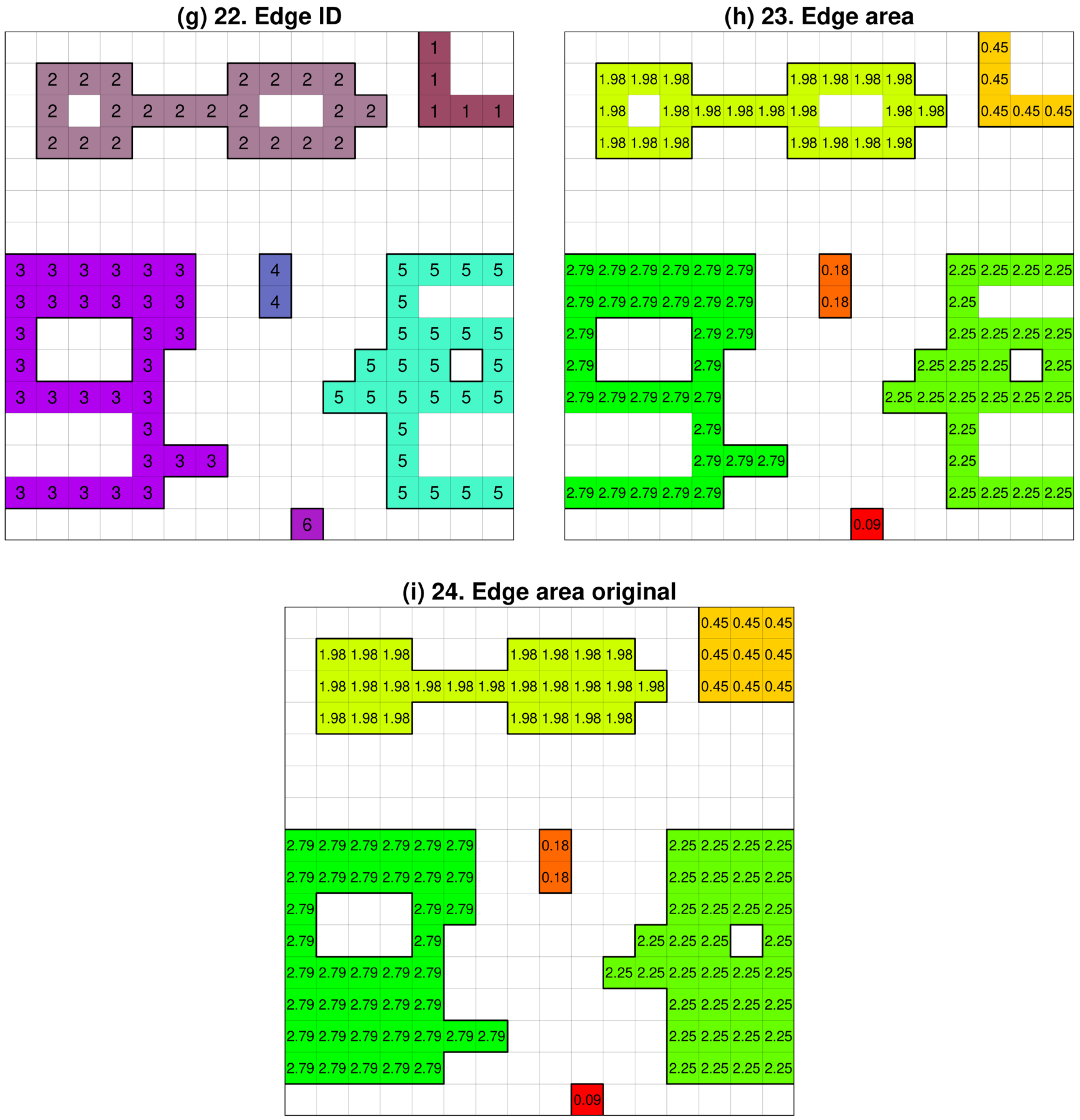

Figure 13. Edge and core area metrics. Metric 16: Core is the binary core and non-core classification (a). Metric 17: Core ID is the core identification (b). Metric 18: Core area is the core area in hectares (c). Metric 19: Core area original is the core area for the original fragment in hectares (d). Metric 20: Number of cores is the number of cores for the original fragment (e). Metric 21: Edge is the binary edge and not-edge classification (f). Metric 22: Edge ID is the edge identification (g). Metric 23: Edge area is the edge area in hectares (h). Metric 24: Edge area original is the edge area for the original fragment in hectares (i). Each cell of the toy landscape has 30 m of side length, and the edge depth was chosen as 30 m in this illustrative example.

Percentage of core and edge metrics (Figure 14): considering a binary habitat map, each cell of the map presents a value of the proportion of core or edge area within a circle moving window with a

given size, centered in the focal cell (amount of core or edge cells/total number of cells in the moving window). It varies between 0 and 100% (**Metric 25: Percentage of core** and **Metric 26: Percentage of edges**). Edge depths considered: 30 m, 60 m, 120 m, 240 m. Buffer radius represented half the size of a circular moving window, e.g., for a buffer size of 50 m, the moving window size was 100 m. Buffer radii used: 50 m, 100 m, 150 m, 200 m, 250 m, 500 m, 750 m, 1000 m, 1500 m, 2000 m, 2500 m.

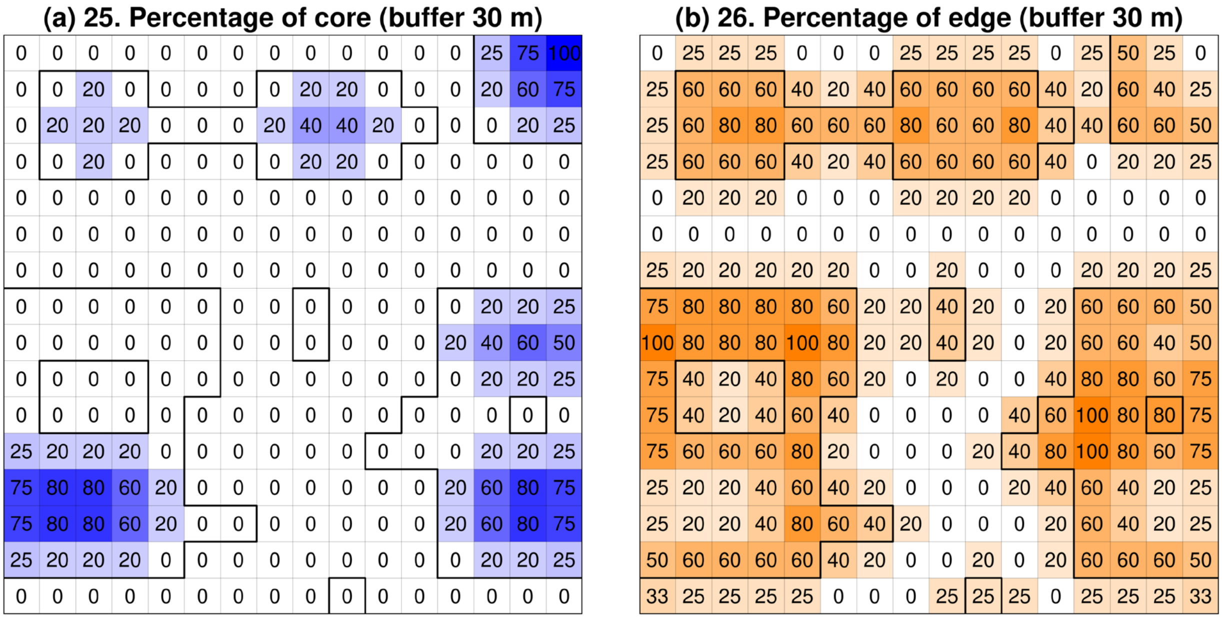

Figure 14. Percentage of core and edge metrics. Metric 25: Percentage of core (a) and Metric 26: Percentage of edge for a circular moving window with 30 m size (b), as an example in the toy landscape.

Perimeter metrics (Figure 15): considering a binary habitat map, the perimeter is the length of the cells located on the sides of a fragment facing the matrix, including any internal holes, in meters (**Metric 27: Perimeter**). Perimeter-area ratio is the ratio between fragment perimeter and fragment area, without a measurement unit (**Metric 28: Perimeter-area ratio**). This is a simple measure of shape complexity, the higher its value, the greater the complexity of the fragment shape. A limitation in using this metric as a shape complexity index is that it varies with the area of the fragment. For example, holding shape constant, an increase in fragment area will cause a decrease in the perimeter-area ratio. Edge depths considered: 30 m. Non-habitat cells were assigned with NULL values.

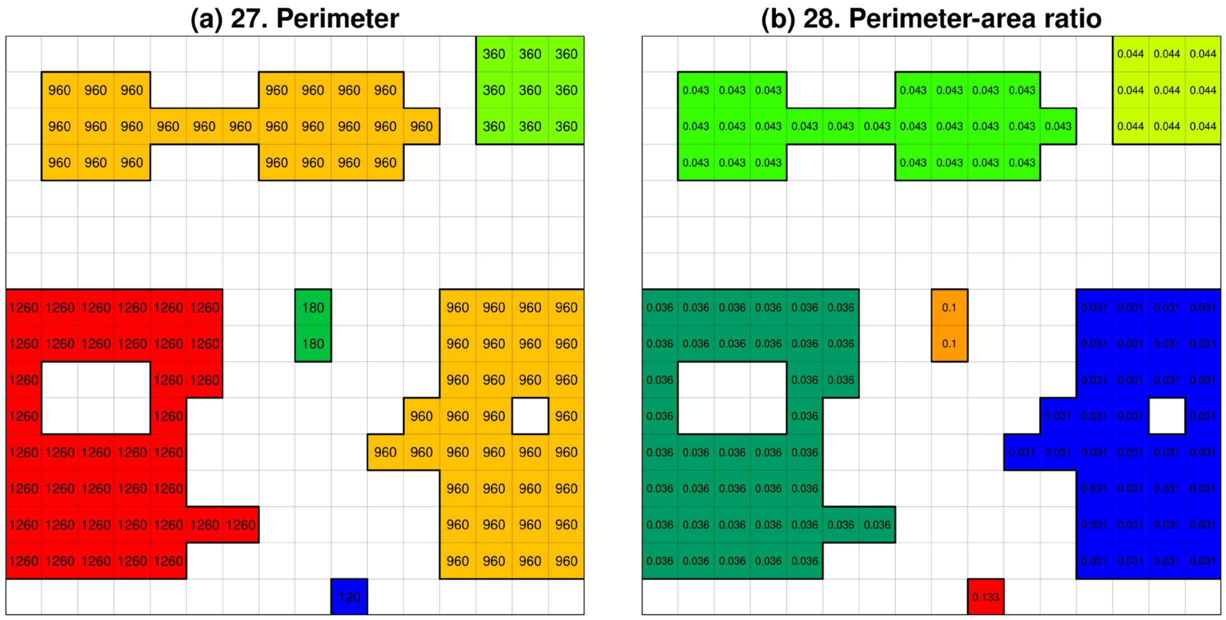

Figure 15. Perimeter metrics. Metric 27: Perimeter (m) is the number of pixel sides of a fragment facing the matrix (a). Metric 28: Perimeter-area ratio is a shape complexity metric (b).

Distance metrics (Figure 16): considering a binary habitat map, distance metrics are based on Euclidean distance mapping. We create the Euclidean distance maps from the edges (contact of the edge pixel with the matrix) of the fragments, in meters. The outside distance mapped the isolation between the fragments, i.e., for each cell outside the edge habitat (non-habitat) was given a positive value, which increases as you move away from the edge pixel and outward from the habitat fragment (**Metric 29: Distance outside**). The inside distance mapped the continuum edge effect and habitat core, i.e., for each cell inside the edge habitat (habitat) were given a negative value, which increases as you move away from the edge pixel towards the interior of the habitat fragment (**Metric 30: Distance inside**). These maps, inside and outside distances were combined (summed) in a metric by summing outside and inside distance metrics, jointly representing the effects of isolation, edge and core effects (**Metric 31: Distance**).

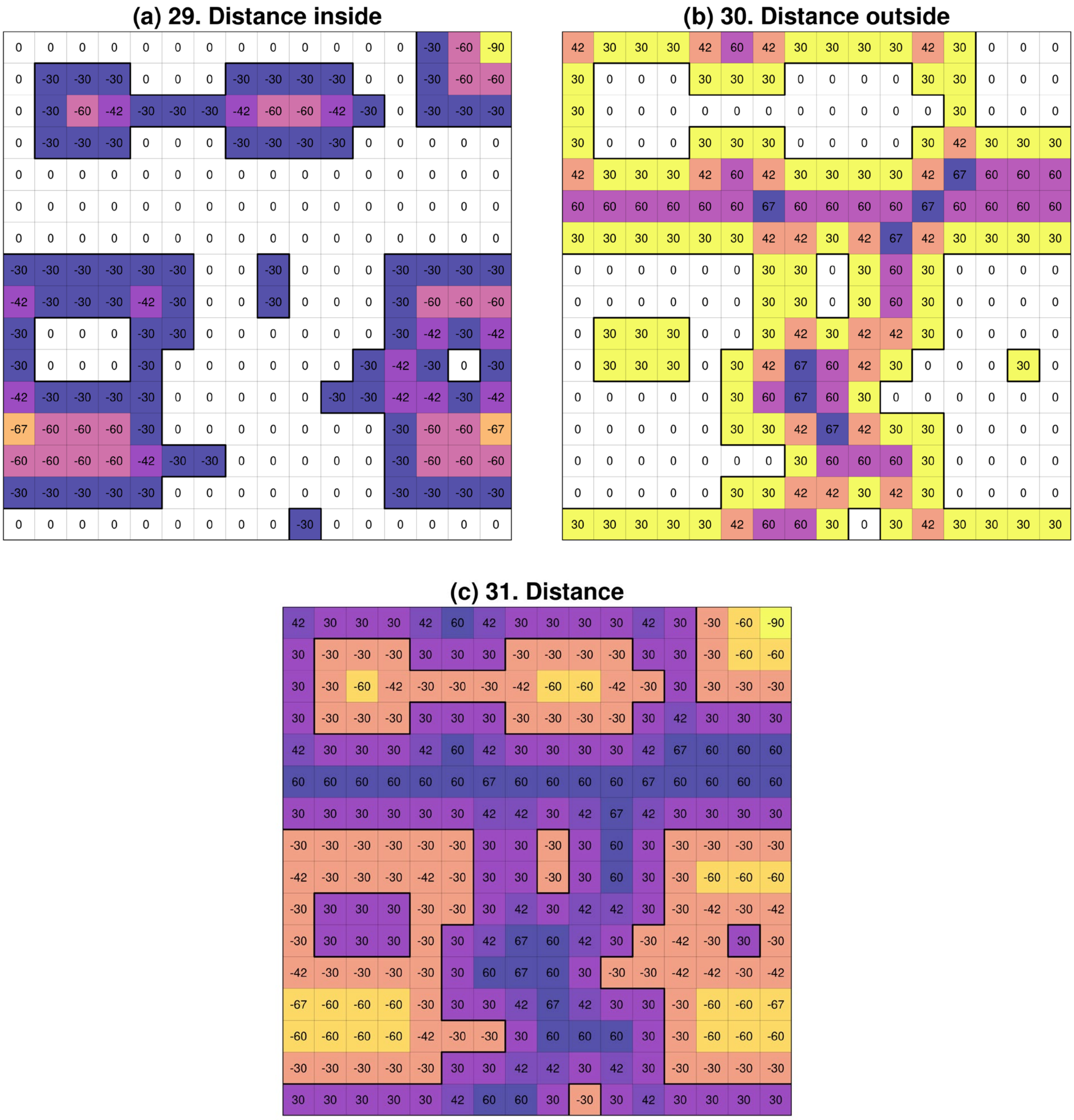

Figure 16. Distance metrics. Metric 29: Distance inside is the distance inside from the habitat with negative values (a). Metric 30: Distance outside is the outside distance from the habitat with positive values (b). Metric 31: Distance is the distance to habitat (c) (Forest Vegetation and Natural Vegetation only) resulting from summing Distance inside (m) and Distance outside (m).

Structural connectivity metrics (Figure 17): considering a binary habitat map, these metrics represent the area of habitat structurally connected to a patch, considering corridors, branches, and possibly other patches (if the corridor connects these patches). In practice, it is calculated as the difference between the fragment area and the patch area. When a patch has no corridors or branches, its structural connectivity equals zero (i.e., it is not structurally connected to any other habitat). Each patch cell is assigned to a structural connectivity value (**Metric 32: Structural connectivity**). The

structural connected area was calculated from the original fragment, where the structural connectivity of the patches was associated with the fragment (**Metric 33: Structural connected area**). The definition of structural connectivity depends on what is considered patch, corridor, and branch, so this metric depends on the edge depth value considered. For the Atlantic Forest the depth of the chosen edge was 30 m. The unit used to calculate the area was hectares. Non-habitat cells were assigned with NULL values.

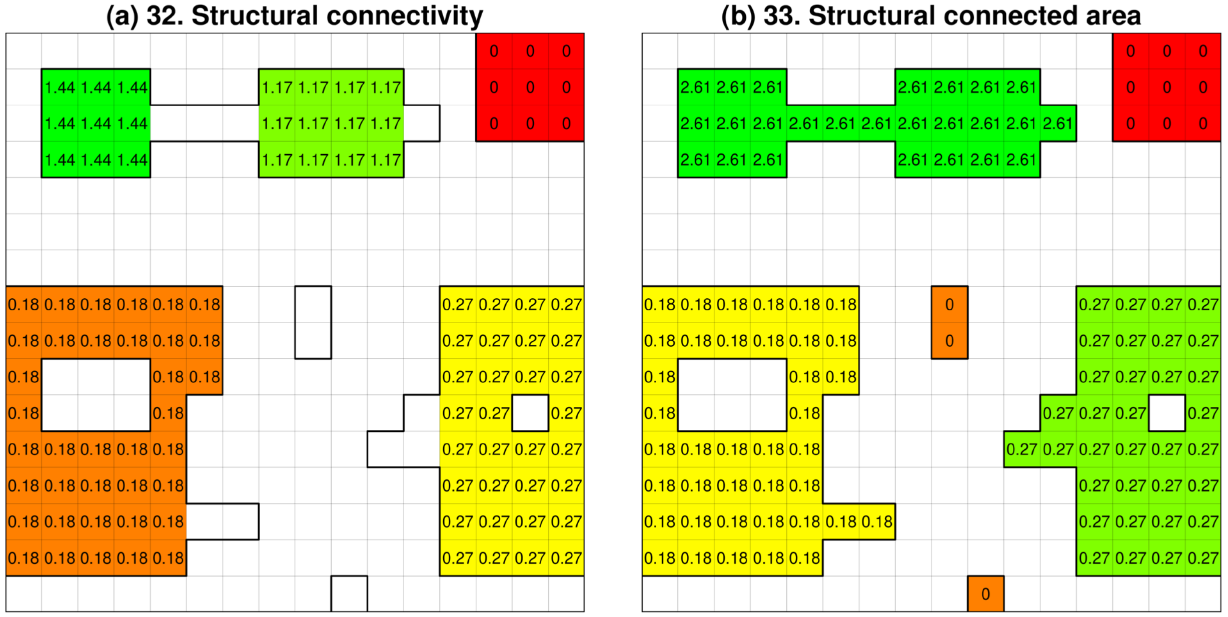

Figure 17. Structural connectivity metrics. Metric 32: Structural connectivity is the area of habitat structurally connected to a patch only for patches in hectares (a). Metric 33: Structural connected area is the area of structurally connected habitat to fragments in hectares (b).

Functional connectivity metrics (Figure 18): considering a binary habitat map, these metrics represent the habitat area functionally linked to a fragment, considering the ability of an organism to cross non-habitat gaps (gap-crossing value). First, the fragments were expanded/dilated by half the value of the organism's gap-crossing capacity (e.g., if an organism crosses 60 m, the fragments are dilated by 30 m along their entire perimeter) (**Metric 34: Functionally connected dilation**). Then, the fragments that are at the shortest distance from the gap-crossing were grouped, receiving the same ID (**Metric 35: Functionally connected ID**). Then, the area of these fragments with the same ID was summed (**Metric 36: Functionally connected area**). Finally, to obtain the functional connectivity, the difference between the functionally connected area and the fragment area was calculated, representing how much habitat is accessible from a habitat fragment for an organism with a given gap-crossing ability, excluding the area of the very same fragment (**Metric 37: Functional connectivity**). Crossing capacities considered for the Atlantic Forest: 60 m, 120 m, 180 m, 240 m,

300 m, 600 m. The unit used to calculate the area was hectares. Non-habitat cells were assigned with NULL values.

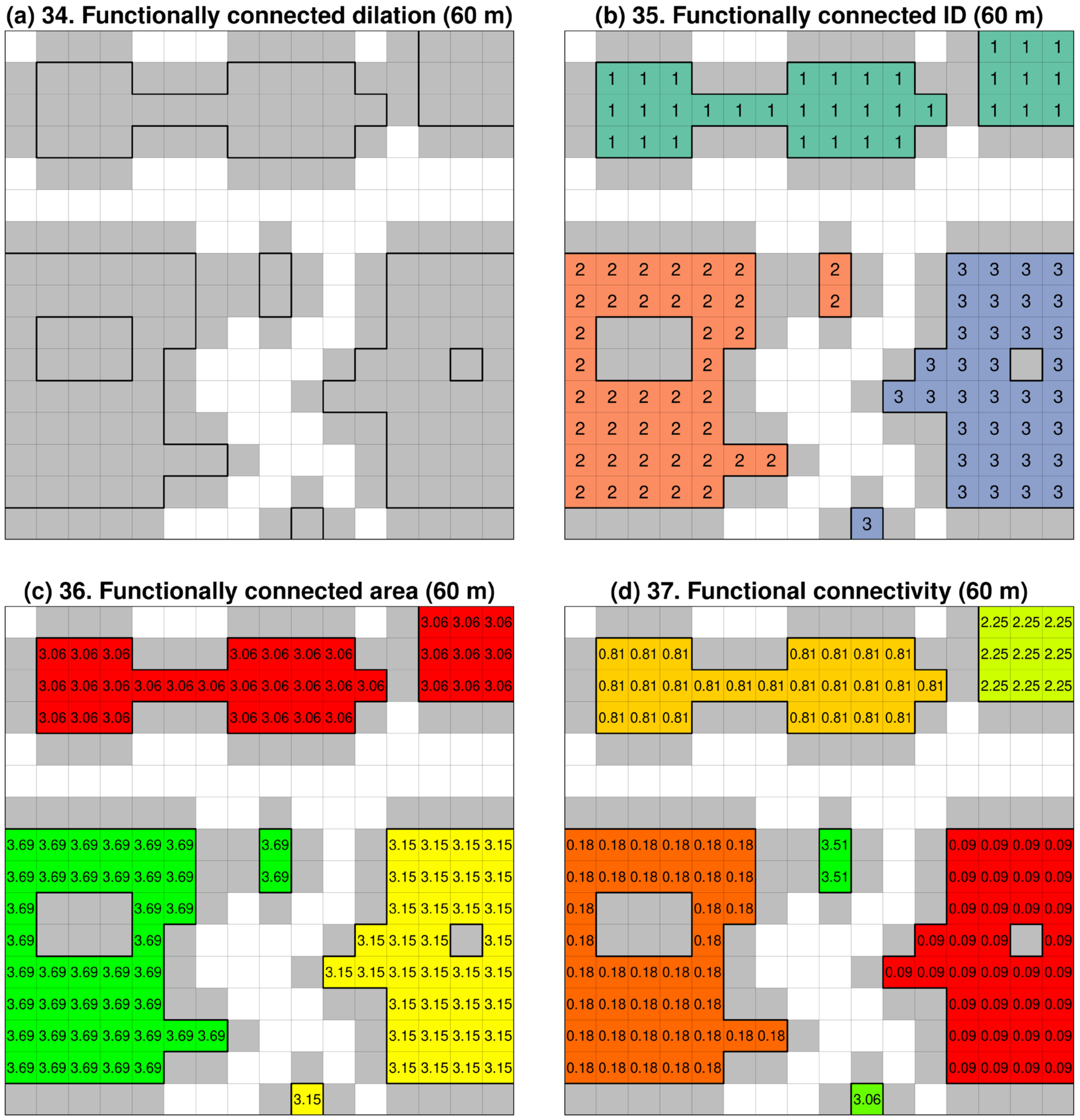

Figure 18. Functional connectivity metrics. Metric 34: Functionally connected dilation (60 m) is the dilation of fragments for gap-crossing of 30 m (gray pixels) (a). Metric 35: Functionally connected ID (60 m) is the functionally connected area identification (b). Metric 36: Functionally connected area is the fragment area connected in hectares (c). Metric 37: Functional connectivity (60 m) is the functional connectivity in hectares (d).

Landscape diversity metrics (Figure 19): considering a multi-class map, each cell of the map presented a value of the diversity of LULC classes within a square moving window with a given size,

centered in the focal cell. Diversity indices (Shannon and Simpson) consider the number of classes in each class cell within the moving window of analysis. Shannon's diversity values are positive and vary between 0 and infinity (**Metric 38: Landscape diversity (Shannon)**) and Simpson's diversity values vary between 0 and 1 (**Metric 39: Landscape diversity (Simpson)**). Buffer radius represented half the size of a square moving window, e.g., for a buffer size of 50 m, the moving window size was 100 m x 100 m. Buffer radii used: 50 m, 100 m, 150 m, 200 m, 250 m, 500 m, 750 m, 1000 m, 1500 m and 2000 m.

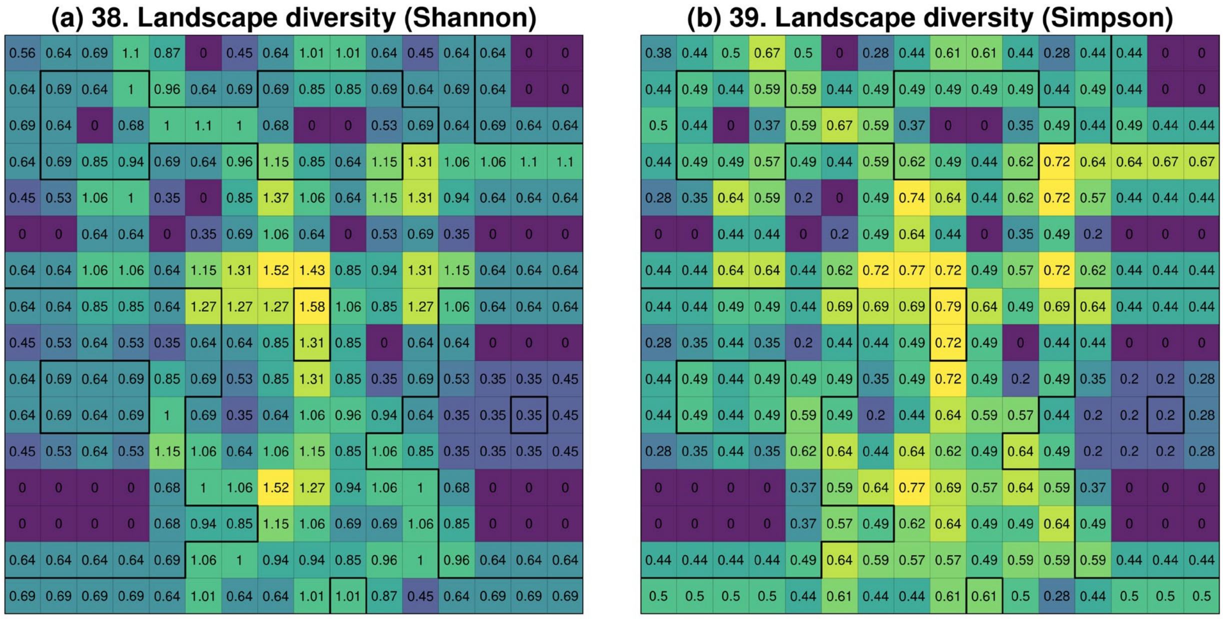

Figure 19. Landscape diversity metrics. Metric 38: Landscape diversity (Shannon) (a) and Metric 39: Landscape diversity (Simpson) (b) indices for a square moving window of 100 m x100 m in size (1 ha), in the illustrative example for the toy landscape.

Topographic metrics

We calculated six metrics of topography using a Digital Elevation Model (DEM) map from FABDEM v1.2 (Hawker et al. 2022) (Table 4). We used two GRASS GIS (Neteler et al. 2012) software modules: *r.slope.aspect* (Hofierka et al. 2009) and *r.geomorphon* (Stepinski and Jasiewicz 2011, Jasiewicz and Stepinski 2013, Libohova et al. 2016).

Table 4. Description of the topographic metrics provided in ATLANTIC SPATIAL.

Metric	Short description	Values	Reference
1. Elevation	Digital representation of elevations (or height) in meters through the Digital Elevation Model (DEM)	Meters	Hawker et al. (2022)
2. Slope	Inclination from the horizontal stated in degrees	Degrees (0º to 90º)	Hawker et al. (2022)
3. Aspect	Direction that slopes are facing counterclockwise from East in degrees: 90 degrees is North, 180 is West, 270 is South, 360 is East	Degrees (0º to 360º)	Hawker et al. (2022)
4. Profile curvature	Curvature in the direction of the steepest slope in 1/m. A curvature of 0.05 corresponds to a radius of curvature of 20 m and positive and negative values represent convex and concave forms, respectively	Units	Hawker et al. (2022)
5. Tangential curvature	Curvature in the direction of the contour tangent in 1/m. A curvature of 0.05 corresponds to a radius of curvature of 20 m and positive and negative values represent convex and concave forms, respectively	Units	Hawker et al. (2022)
6. Geomorphon	Classification and mapping of landform elements from a DEM based on the principle of pattern recognition (geomorphon)	Classes: flat (1), peak (2), ridge (3), shoulder (4), spur (5), slope (6), hollow (7), footslope (8), valley (9), pit (10)	Jasiewicz and Stepinski (2013)

Hydrological metrics

We calculated four hydrological metrics using a Digital Elevation Model (DEM) map from FABDEM v1.2 (Hawker et al. 2022) (Table 5). Due to the large extension, we first calculated these metrics for hydrologically smaller basins using HydroBASINS level 5 (Lehner and Grill 2013); after that, we merged the results into single maps for the four metrics. We used two GRASS GIS (Neteler et al. 2012) modules to calculate the potential streams and springs: *r.watershed* and *r.stream.extract*, both with threshold = 100. With the potential streams and strings done, we deleted the lines and points, respectively, that overlap with masses of water from HydroLAKES (Messager et al. 2016) and official masses of water from three countries: Brazil (Instituto Brasileiro de Geografia e Estatística – IBGE; IBGE, 2021), Argentina (Instituto Geográfico Nacional – IGN; IGN, 2023) and Paraguay (Instituto Nacional de Estadística – INE; INE, 2023). Stream Euclidean distance was generated using *r.grow.distance* GRASS GIS (Neteler et al. 2012) module with metric = euclidean, and spring kernel density was generated using *v.kernel* module with radius varying (50 m, 100 m, 150 m, 200 m, 250 m, 500 m, 750 m, 1000 m, 1500 m, 2000 m, 2500 m) and kernel = gaussian.

Table 5. Hydrological metrics description.

Metric	Short description	Values	Scale (buffer radius in meters)	Reference
1. Stream	Potential streams generated from DEM	0 and 1	NA	Holmgren (1994)
2. Stream distance	Euclidean distance from potential streams generated from DEM	Meters	NA	Holmgren (1994)
3. Spring	Potential springs generated from DEM	0 and 1	NA	Holmgren (1994)
4. Spring density	Density (kernel) of potential springs generated from DEM	Units	50, 100, 150, 200, 250, 500, 750, 1000, 1500, 2000, 2500	Okabe et al. (2009)

Anthropogenic metrics

We calculated 13 anthropogenic metrics (Table 6) represented by Euclidean distances outside (positive values) from roads, railways, protected areas, indigenous territories, quilombola territories, and the categories of grouped LULC classes: pasture, temporary crop, perennial crop, forest plantation, urban areas, mining, and water (Table 2; Figure 3b). These metrics can be used to represent the different forms of human activity and possibly their impact at the landscape scale. We used the *r.grow.distance* GRASS GIS (Neteler et al. 2012) module with metric = euclidean.

Table 6. Description of Anthropogenic metrics available in ATLANTIC SPATIAL.

Metric	Short description	Values	Reference
1. Distance from roads	Euclidean distance from roads	Meters	Ribeiro et al. (2009)
2. Distance from railways	Euclidean distance from railways	Meters	Ribeiro et al. (2009)
3. Distance from roads and railways	Euclidean distance from roads and railways	Meters	Ribeiro et al. (2009)
4. Distance from protected areas	Euclidean distance from protected areas	Meters	Ribeiro et al. (2009)
5. Distance from indigenous territories	Euclidean distance from indigenous territories	Meters	Ribeiro et al. (2009)
6. Distance from quilombola territories	Euclidean distance from quilombola territories	Meters	Ribeiro et al. (2009)
7. Distance from the forest plantation	Euclidean distance from forest plantation	Meters	Ribeiro et al. (2009)
8. Distance from the pasture	Euclidean distance from pasture	Meters	Ribeiro et al. (2009)
9. Distance from the temporary crop	Euclidean distance from temporary crop	Meters	Ribeiro et al. (2009)
10. Distance from the perennial crop	Euclidean distance from perennial crop	Meters	Ribeiro et al. (2009)
11. Distance from the urban areas	Euclidean distance from urban areas	Meters	Ribeiro et al. (2009)
12. Distance from the mining	Euclidean distance from mining	Meters	Ribeiro et al. (2009)
13. Distance from the water	Euclidean distance from water (lakes and rivers)	Meters	Ribeiro et al. (2009)

4. Project personnel

None.

Class III. Dataset status and accessibility

A. Status

1. Latest update

September 2025.

2. Latest archive date

September 2025.

3. Metadata status

Last updated in September 2025, version submitted.

4. Data verification

Last updated in September 2025, version submitted.

B. Accessibility

1. Storage location and medium

The ATLANTIC SPATIAL dataset guide table (ATLANTIC_SPATIAL.csv) with all metric descriptions and links to files on Zenodo repositories can be accessed as supporting information for this Data Paper publication in Ecology. In Zenodo we also provide all the R codes used to calculate the metrics (Vancine and Nieburh 2025; https://doi.org/10.5281/zenodo.14814102), and the codes of the R package *atlanticr* (Vancine et al. 2025; https://doi.org/10.5281/zenodo.14751252). Vector and rasters (spatial components) can be accessed in multiple Zenodo repositories (Table 7), organized by thematic groups of layers. Due to file size limitations, these datasets cannot be included as supporting information with the publication in Ecology.

1) Table 7. Variable ids and Zenodo repositories titles, links, and DOIs for the ATLANTIC SPATIAL dataset. We have separated the dataset into multiple Zenodo repositories due to file size and number limitations.

Variable ids	Zenodo repository title	Zenodo repository link	Zenodo DOI
000-004<br>041-064	ATLANTIC SPATIAL - Habitat	https://zenodo.org/records/17180586	https://doi.org/10.5281/zenodo.17180586
005-040; 375-388	ATLANTIC SPATIAL - Fragment	https://zenodo.org/records/14574196	https://doi.org/10.5281/zenodo.14574196
065-112	ATLANTIC SPATIAL - Core 30|60|90m Forest	https://zenodo.org/records/14529477	https://doi.org/10.5281/zenodo.14529477
113-144	ATLANTIC SPATIAL - Core 120|240m Forest	https://zenodo.org/records/14574249	https://doi.org/10.5281/zenodo.14574249
145-189	ATLANTIC SPATIAL - Edge 30|60|90m Forest	https://zenodo.org/records/14529566	https://doi.org/10.5281/zenodo.14529566
190-219	ATLANTIC SPATIAL - Edge 120|240m Forest	https://zenodo.org/records/14577603	https://doi.org/10.5281/zenodo.14577603
220-267	ATLANTIC SPATIAL - Core 30|60|90m Natural	https://zenodo.org/records/14577592	https://doi.org/10.5281/zenodo.14577592
268-299	ATLANTIC SPATIAL - Core 120|240m Natural	https://zenodo.org/records/14577598	https://doi.org/10.5281/zenodo.14577598
300-344	ATLANTIC SPATIAL - Edge 30|60|90m Natural	https://zenodo.org/records/14529647	https://doi.org/10.5281/zenodo.14529647
345-374	ATLANTIC SPATIAL - Edge 120|240m Natural	https://zenodo.org/records/14577617	https://doi.org/10.5281/zenodo.14577617
389-436	ATLANTIC SPATIAL - Connectivity	https://zenodo.org/records/14529380	https://doi.org/10.5281/zenodo.14529380
437-446	ATLANTIC SPATIAL - Diversity Shannon	https://zenodo.org/records/14529710	https://doi.org/10.5281/zenodo.14529710
447-456	ATLANTIC SPATIAL - Diversity Simpson	https://zenodo.org/records/14529750	https://doi.org/10.5281/zenodo.14529750
457-462	ATLANTIC	https://zenodo.org/rec	https://doi.org/10.5281

Variable ids	Zenodo repository title	Zenodo repository link	Zenodo DOI
	SPATIAL - Topographic	ords/14529237	/zenodo.14529237
463-476	ATLANTIC SPATIAL - Hydrological	https://zenodo.org/records/14500641	https://doi.org/10.5281/zenodo.14500641
477-502	ATLANTIC SPATIAL - Anthropogenic	https://zenodo.org/records/14529355	https://doi.org/10.5281/zenodo.14529355

Besides the direct download through the Zenodo repositories, we also created the R package *atlanticr* (Vancine et al. 2025; https://doi.org/10.5281/zenodo.14751252). This R package provides a table with all ATLANTIC SPATIAL metrics and their information "atlantic_spatial" (the same as ATLANTIC_SPATIAL.csv in supplementary material), beyond a function to download the vector and rasters, "atlantic_spatial_download()", from the corresponding Zenodo repositories. Each raster layer comprises two files: a GeoTiff (.tif) file and a TFW (.tfw) file. The GeoTiff files are the ones providing geographic information for the variables and in most Geographical Information Systems they are enough for reading and using the data. The TFW (.tfw) are auxiliary files created due to the raster compression process, and that can be necessary for usage of the data in some Software.

Below we demonstrate a simple example to use the function *atlantic_spatial_download()* to download the raster with id = 1 (land use and land cover raster), whose details can be checked in the the *atlanticr::atlantic_spatial* table (the same as ATLANTIC_SPATIAL.csv).

```
# install package
install.packages("remotes")
remotes::install_github("mauriciovancine/atlanticr")

# load package
library(atlanticr)

# list files
head(atlanticr::atlantic_spatial)

# file download
atlanticr::atlantic_spatial_download(id = 1, path = ".")
```

Furthermore, the *atlanticr* R package also facilitates access to other data papers from the *ATLANTIC: Data Papers from a biodiversity hotspot*, providing an easy way to integrate biological and environmental data for the Atlantic Forest (Vancine et al. 2025).

2. Contact persons

Maurício Humberto Vancine (mauricio.vancine@gmail.com), Bernardo Brandão Niebuhr (bernardo_brandaum@yahoo.com.br), or Milton Cezar Ribeiro (miltinho.astronauta@gmail.com).

3. Copyright restrictions

CC BY-NC 4.0: Creative Commons Attribution-Non-Commercial 4.0 International.

4. Proprietary restrictions

a. Release date

None.

b. Citation

Please, cite this data paper when the data are used in publications or teaching events.

c. Disclaimer(s)

None.

5. Costs

None.

Class IV. Data structural descriptors

The dataset contains 1005 files and 267.3 GB. ATLANTIC_SPATIAL.csv contains a table describing the vector and raster files. The Atlantic Forest delimitation vector is available as Geopackage (.gpkg). The 502 rasters are available as GeoTiff (.tif) which contain the main data files and TFW files (.tfw) as an ancillary file. We created the rasters using DEFLATE compression from GDAL (https://gdal.org/en/stable/drivers/raster/gtiff.html), requiring the provision of an auxiliary TFW file (.tfw) for it to be opened in software such as ArcGIS® (https://www.arcgis.com/index.html).

A. Dataset file

1. Identity: ATLANTIC_SPATIAL.csv

2. Size: 19 columns and 503 rows records, including header row, 290 KB.

3. Format and storage mode: comma-separated values (.csv).

4. Header information: See column descriptions in section B.

5. Alphanumeric attributes: Mixed.

6. Special characters/fields: None.

7. Authentication procedures: None.

B. Variable information

1) Table 8. **Information in the ATLANTIC SPATIAL dataset**. Description of the fields related to the study site of the ATLANTIC_SPATIAL.csv.

Variable identify	Variable description	Level/Range/Description	Example
**id**	Identification code for each metric	000-502	006
**metric**	Metric names	Detailed name of metric in text format	atlantic_spatial_forest_vegetation_fragment_area
**metric_group**	Description of metric groups	anthropogenic, hydrological, landscape, topographic	landscape
**metric_type**	Detailed description of metric types	(not applicable)	fragment_area
**metric_description**	Detailed description of metrics	Detailed description of metrics in text format	forest vegetation fragment area
**value**	Metric values	Detailed metrics values in text and number formats	0.09 to infinity
**value_description**	Detailed description of metric values	Detailed description of metric values in text format	area
**unit**	Metric units	1/m, angles in degrees, binary, categorical, discrete, hectares, meters, meters/hectares, proportion, unit, unitary	hectares

Variable identify	Variable description	Level/Range/Description	Example
**lulc_class**	Land use and land cover classes	forest_plantation,<br>forest_vegetation,<br>mining,<br>multiple,<br>natural_vegetation,<br>pasture,<br>perennial_crop,<br>temporary_crop,<br>urban_areas,<br>water	forest_vegetation
**edge_depth_m**	Edge depth for different metrics in meters. Edge depth is the minimum distance at which cells are classified as edges, those that are further away are classified as cores	30-240	NA
**gap_crossing_m**	Gap-crossing for different metrics in meters. Gap-crossing considers the ability of an organism to cross non-habitat gaps, characterizing the distance to functional connectivity	60-600	NA
**scale_buffer_radius_m**	Scale for different metrics in meters. Scale is the radius of the buffer to which the moving window is rotated to impute the effect of different scales on landscape metrics	50-10,000	NA
**resolution**	Raster pixel width and height	30	30
**file_name**	File names for this metric	Multiple metric name files	006_atlantic_spatial_forest_vegetation_fragment_area.tif
**file_size**	File sizes for this	Multiple metric size	0.398

Variable identify	Variable description	Level/Range/Description	Example
	metric	files in GB	
**zenodo_repository**	Zenodo repository name	Multiple repository names	ATLANTIC SPATIAL - Fragment
**zenodo_link_main**	Link to the main files on Zenodo	Multiple links	https://zenodo.org/records/14574196/files/006_atlantic_spatial_forest_vegetation_fragment_area.tif?download=1&preview=1
**zenodo_link_auxiliary**	Link to the auxiliary files on Zenodo	Multiple links	https://zenodo.org/records/14574196/files/006_atlantic_spatial_forest_vegetation_fragment_area.tfw?download=1&preview=1
**zenodo_doi**	Link to the DOI on Zenodo	Multiple links	https://doi.org/10.5281/zenodo.14574196

C. Data anomalies

If no information is available, this was indicated by “NA”.

Class V. Supplemental descriptors

A. Data acquisition

1. Data forms or acquisition methods

Download and curation of openly available data sources and custom code post-processing.

2. Location of completed data forms

None.

3. Data entry verification procedures

None.

B. Quality assurance/quality control procedures

None.

C. Related materials

None.

D. Computer programs and data-processing algorithms

Software

All the landscape, topographic, hydrological, and anthropogenic metrics were processed using GRASS GIS 8.3 (Neteler et al. 2012) and R language 4.3 (R Core Team, 2023) with the aid of the *rgrass* package (Bivand, 2023). GRASS GIS (Geographical Resources Analysis Support System) is a free and open-source Geographic Information System (GIS), created around 1985, and in continuous development. It provides over 400 well-documented and peer-reviewed modules for spatial analysis, modeling, and visualization, and is widely used in academia, business, and public administration. Developed by a global community, GRASS GIS runs natively on major operating systems and is particularly recognized for its applications in environmental modeling. Its architecture is optimized for handling and processing large volumes of geospatial data, making it particularly suitable for complex environmental modeling and high-performance geocomputation (Neteler et al. 2012). All landscape metrics were calculated using custom functions based on LSMetrics and translated to R (https://doi.org/10.5281/zenodo.3736443; Niebuhr et al. 2020).

E. Archiving

All codes used to calculate the metrics are available on Zenodo (https://doi.org/10.5281/zenodo.14814102). These scripts represent the step-by-step process for calculating the metrics, allowing the process to be completely reproducible. For example, the script “01_01_download_limits.R” downloads the Atlantic Forest boundary, and “01_02_download_landscape.R” downloads the land use and land cover layers from MapBiomas using an integration with Google Earth Engine. Likewise, the other scripts download the other input sources of data, describe their process of import into GRASS GIS, and the computation of the different types of metrics presented in this dataset. By making these scripts available, we believe that this approach to calculate these metrics can be replicated for other biomes or regions of the world, or for other timestamps using the available data for the Atlantic Forest. However, some steps were omitted or need to be performed in addition to the scripts for the full analysis to be performed. For example, the final boundary of the Atlantic Forest was manually edited and cannot be reproduced using scripts (but a detailed description is available in Vancine et al. 2024). Another example that users wishing to reproduce the analyses should be aware of is that we used GNU/Linux to calculate the metrics. GRASS GIS works integrated with R through *rgrass* R package, and to do so, you need to specify the GRASS GIS directory, which on GNU/Linux can be accessed as follows: `system("grass --config path", inter = TRUE)`. On Microsoft Windows®, it is needed to specify a different path, for example: "C:/Program Files/GRASS GIS 8.3". This can be a bit confusing for new users of these software integrations.

1. Archival procedures

All files have been deposited in Zenodo repositories.

2. Redundant archival sites

None.

F. Publications and results

Vancine et al. (2024) used part of this dataset to describe the spatiotemporal landscape structure of the Atlantic Forest.

G. History of dataset usage

1. Data request history

None.

2. Dataset updates history

None.

3. Review history

None.

4. Question and comments from secondary users

None.

CRediT authorship contribution statement

MHV: Conceptualization, Data curation, Formal analysis, Investigation, Methodology, Software, Visualization, Writing— original draft, Writing—review and editing. **BBN**: Conceptualization, Data curation, Formal analysis, Investigation, Methodology, Software, Visualization, Writing—original draft, Writing—review and editing. **RLM**: Conceptualization, Data curation, Investigation, Software, Writing—review and editing. **JEFO**: Conceptualization, Data curation, Writing—review and editing. **VT**: Data curation, Conceptualization, Writing—review and editing. **RB**: Data curation, Writing—review and editing. **RSCA**: Data curation, Writing—review and editing. **EMZ**: Data curation, Writing—review and editing. **VCS**: Data curation, Writing—review and editing. **JGRG**: Conceptualization, Data curation, Writing—review and editing. **JWR**: Conceptualization, Data curation, Investigation, Software, Writing—review and editing. **CDA**: Writing—review and editing. **CHG**: Data curation, Methodology, Software, Writing—review and editing. **MG**: Writing—review and editing. **MCR**: Conceptualization, Data curation, Funding acquisition, Investigation, Methodology, Project administration, Resources, Software, Supervision, Writing—original draft, Writing—review and editing.

Acknowledgments

We thank all members of the Spatial Ecology and Conservation Lab (LEEC) from UNESP (https://github.com/LEEClab) for their help with fruitful discussions. We thank all open data enthusiasts and the contributors of the ATLANTIC collection for their efforts to encourage reproducible science practices. We thank all members of the SDMGroup from UNESP (https://github.com/LEEClab/SDMGroup) for all the fruitful discussions and suggestions during our meetings. We would also like to thank everyone who kindly tested the R package *atlanticr*. We thank everyone who helped us with reflections in respect to the quilombola territories of Brazil.

Literature Citations

Adorno, B. F. C. B., A. J. Piratelli, E. Hasui, M. C. Ribeiro, and P. G. Vaz. 2025. Relative fire-proneness of land cover types in the Brazilian Atlantic forest. Journal of Environmental Management 374:124066.

Almeida-Gomes, M., and C. F. D. Rocha. 2014. Landscape connectivity may explain anuran species distribution in an Atlantic forest fragmented area. Landscape Ecology 29:29–40.

Amaral, S., J. P. Metzger, M. Rosa, B. V. Adorno, G. C. Gonçalves, and L. F. Guedes Pinto. 2025. Alarming patterns of mature forest loss in the Brazilian Atlantic Forest. Nature Sustainability:1–9.

Anderle, M., C. Paniccia, M. Brambilla, A. Hilpold, S. Volani, E. Tasser, J. Seeber, and U. Tappeiner. 2022. The contribution of landscape features, climate and topography in shaping taxonomical and functional diversity of avian communities in a heterogeneous Alpine region. Oecologia 199:499–512.

Anunciação, P. R., R. Ernst, F. Martello, M. H. Vancine, L. M. T. D. Carvalho, and M. C. Ribeiro. 2023. Climate-driven loss of taxonomic and functional richness in Brazilian Atlantic Forest anurans. Perspectives in Ecology and Conservation 21:274–285.

Awade, M., and J. P. Metzger. 2008. Using gap-crossing capacity to evaluate functional connectivity of two Atlantic rainforest birds and their response to fragmentation. Austral Ecology 33:863–871.

Baguette, M., S. Blanchet, D. Legrand, V. M. Stevens, and C. Turlure. 2013. Individual dispersal, landscape connectivity and ecological networks. Biological Reviews 88:310–326.Beale, M. M., A. A. Knopff, J. M. Foca, K. H. Knopff, L. Amos, and W. Franklin. 2025. Variable zone of influence responses by large mammals: Implications for conservation planning. Ecosphere 16:e70393.

Beca, G., M. H. Vancine, C. S. Carvalho, F. Pedrosa, R. S. C. Alves, D. Buscariol, C. A. Peres, M.

C. Ribeiro, and M. Galetti. 2017. High mammal species turnover in forest patches immersed in biofuel plantations. Biological Conservation 210:352–359.

Bélisle, M. 2005. Measuring Landscape Connectivity: The Challenge of Behavioral Landscape Ecology. Ecology 86:1988–1995.

Bello, C., M. Galetti, D. Montan, M. A. Pizo, T. C. Mariguela, L. Culot, F. Bufalo, F. Labecca, F. Pedrosa, R. Constantini, C. Emer, W. R. Silva, F. R. da Silva, O. Ovaskainen, and P. Jordano. 2017. Atlantic frugivory: a plant-frugivore interaction data set for the Atlantic Forest. Ecology 98:1729–1729.

Bello, C., M. Galetti, M. A. Pizo, L. F. S. Magnago, M. F. Rocha, R. A. F. Lima, C. A. Peres, O. Ovaskainen, and P. Jordano. 2015. Defaunation affects carbon storage in tropical forests. Science Advances 1:e1501105.

Benzeev, R., S. Zhang, M. A. Rauber, E. A. Vance, and P. Newton. 2023. Formalizing tenure of Indigenous lands improved forest outcomes in the Atlantic Forest of Brazil. PNAS Nexus 2:pgac287.

Bertassoni, A., R. T. Costa, J. A. Gouvea, R. de C. Bianchi, J. W. Ribeiro, M. H. Vancine, and M. C. Ribeiro. 2019. Land-use changes and the expansion of biofuel crops threaten the giant anteater in southeastern Brazil. Journal of Mammalogy 100:435–444.

Bicudo da Silva, R. F., E. Moran, A. Viña, J. D. A. Millington, Y. Dou, S. A. Vieira, M. C. Lopez, and J. Liu. 2023. Toward a forest transition across the Brazilian Atlantic Forest biome. Frontiers in Forests and Global Change 6:1071495.

Bivand R. 2023. *rgrass*: Interface Between 'GRASS' Geographical Information System and 'R'. R package version 0.3-9. Available at: https://CRAN.R-project.org/package=rgrass.

Bonfim, F. C. G., P. Dodonov, and E. Cazetta. 2021. Landscape composition is the major driver of the taxonomic and functional diversity of tropical frugivorous birds. Landscape Ecology 36:2535–2547.

Boscolo, D., B. Nobrega Rodrigues, P. A. Ferreira, L. E. Lopes, V. R. Tonetti, I. C. Reis dos Santos, J. A. Hiruma-Lima, L. Nery, K. Baptista de Lima, J. Perozi, A. V. L. Freitas, B. F.

Viana, C. Antunes-Carvalho, D. de S. Amorim, F. Freitas de Oliveira, M. Groppo, M. L. Absy, R. J. de Almeida-Scabbia, A. Alves-Araújo, F. W. de Amorim, P. A. P. Antiqueira, Y. Antonini, C. Aoki, D. dos Santos Aragão, T. C. T. Balbino, M. da Silva Ferreira Bandeira, B. C. Barbosa, M. R. de Vasconcellos Barbosa, G. J. Baronio, L. O. Barros, M. Beal-Neves, V. M. Bertollo, A. D. de Melo Bezerra, C. R. Buzatto, L. T. Carneiro, E. Caron, C. S. Carpim, E. S. Carvalho, T. L. Carvalho, L. J. Carvalho-Leite, M. F. Cascaes, F. S. de Castro, A. Cavalleri, E. Cazetta, M. T. Cerezini, L. F. M. Coelho, R. Colares, G. D. Cordeiro, J. Cordeiro, A. M. da Silva Corrêa, F. V. da Costa, C. Covre, R. D. M. Cruz, O. Cruz-Neto, L. Correia-da-Rocha-Filho, J. H. C. Delabie, M. da Costa Dórea, V. T. do-Nascimento, J. M. Alves dos-Santos, M. Duarte, M. C. Duarte, O. M. P. Duarte, J. H. A. Dutilh, B. P. Emerick, G. dos S. Fabiano, F. H. A. Farache, A. P. G. de Faria, G. W. Fernandes, P. Maria Abreu Ferreira, M. J. Ferreira-Caliman, L. M. N. Ferreira, T. F. Filgueira de Sá, E. V. Franceschinelli, G. A. Franco-Assis, F. Fregolente Faracco Mazziero, B. M. Freitas, J. Freitas, N. A. Galastri, L. Galetto, C. T. Garcia, M. T. Amela García, N. L. Garcia, C. A. Garófalo, I. Gélvez-Zúñiga, C. da S. Goldas, T. J. Guerra, T. M. Guerra, B. Harter-Marques, J. Hipólito, R. Kamke, R. P. Klein, E. B. de A. Koch, P. Landgref-Filho, S. Laroca, C. M. Leandro, R. Lima, T. R. A. de Lima, L. W. Lima-Verde, E. J. de Lírio, A. V. Lopes, A. P. Luizi-Ponzo, I. C. S. Machado, T. Machado, F. S. Magalhães, T. Mahlmann, C. dos S. F. Mariano, T. E. D. Marques, F. Martello, C. F. Martins, M. N. Martins, R. Martins, A. L. S. Mascarenhas, G. de Assis Mendes, M. de S. Mendonça, L. Menini Neto, M. A. Milward-de-Azevedo, A. O. Miranda, P. M. Montoya-Pfeiffer, A. M. Moraes, B. B. Moraes, E. F. Moreira, M. S. Morini, D. Moure-Oliveira, L. F. De Nadai, V. H. Nagatani, M. H. Nervo, F. de Siqueira Neves, J. S. de Novais, É. S. Araújo-Oliveira, J. H. F. de Oliveira, A. J. de S. Pacheco-Filho, L. Palmieri, M. Pareja, M. de A. Passarella, N. da M. Passos, H. F. Paulino-Neto, A. Luna Peixoto, L. C. Pereira, R. A. S. Pereira, B. Pereira-Silva, J. Pincheira-Ulbrich, M. Pinheiro, A. J. Piratelli, L. R. Podgaiski, D. S. Polizello, L. P. do Prado, F. Prezoto, F. R.

de Quadros, E. P. Queiroz, Z. Glebya Maciel Quirino, A. M. Rabello, G. B. P. Rabeschini, M. M. M. Ramalho, F. N. Ramos, L. Rattis, L. H. G. de Rezende, C. Ribeiro, L. J. Robe, E. M. de S. R. Rocha, R. R. Rodrigues, G. Q. Romero, N. Roque, W. de O. Sabino, P. T. Sano, P. da S. S. Reis, F. S. dos Santos, I. Alves dos Santos, F. de A. R. dos Santos, I. Silva dos Santos, R. Sartorello, H. J. Schmitz, M. R. Sigrist, J. C. Silva Junior, A. C. G. e Silva, C. V. C. da Silva, B. S. Alves Vieira Silva, B. L. de F. Silva, C. I. Silva, F. O. da Silva, J. L. S. e Silva, N. S. Silva, O. G. M. da Silva, C. de M. e Silva Neto, E. R. Silva Neto, D. Silveira, M. S. Silveira, R. B. Singer, L. A. S. S. Soares, E. M. Locatelli de Souza, J. M. T. de Souza, J. Steiner, M. C. Teixeira-Gamarra, B. A. Trentin, I. G. Varassin, G. Vila-Verde, V. N. Yoshikawa, E. M. Zanin, M. Galetti, and M. C. Ribeiro. 2023. Atlantic flower–invertebrate interactions: A data set of occurrence and frequency of floral visits. Ecology 104:e3900.

Bovendorp, R. S., F. T. Brum, R. A. McCleery, B. Baiser, R. Loyola, M. V. Cianciaruso, and M. Galetti. 2019. Defaunation and fragmentation erode small mammal diversity dimensions in tropical forests. Ecography 42:23–35.

Bovendorp, R. S., N. Villar, E. F. de Abreu-Junior, C. Bello, A. L. Regolin, A. R. Percequillo, and M. Galetti. 2017. Atlantic small-mammal: a dataset of communities of rodents and marsupials of the Atlantic forests of South America. Ecology 98:2226–2226.

Branco, V. V., C. Capinha, J. Rocha, L. Correia, and P. Cardoso. 2024. SPECTRE: Standardised Global Spatial Data on Terrestrial SPecies and ECosystems ThREats. Global Ecology and Biogeography 34:e13949.

Burnham, R. J., and K. R. Johnson. 2004. South American Palaeobotany and the Origins of Neotropical Rainforests. Philosophical Transactions: Biological Sciences 359:1595–1610.

Carlucci, M. B., V. Marcilio-Silva, and J. M. Torezan. 2021. The Southern Atlantic Forest: Use, Degradation, and Perspectives for Conservation. Pages 91–111 *in* M. C. M. Marques and C. E. V. Grelle, editors. The Atlantic Forest: History, Biodiversity, Threats and Opportunities of the Mega-diverse Forest. Springer International Publishing, Cham.

Carnaval, A. C., E. Waltari, M. T. Rodrigues, D. Rosauer, J. VanDerWal, R. Damasceno, I. Prates, M. Strangas, Z. Spanos, D. Rivera, M. R. Pie, C. R. Firkowski, M. R. Bornschein, L. F. Ribeiro, and C. Moritz. 2014. Prediction of phylogeographic endemism in an environmentally complex biome. Proceedings of the Royal Society B: Biological Sciences 281:20141461.

Cassimiro, I. M. F., M. C. Ribeiro, and J. C. Assis. 2023. How did the animal come to cross the road? Drawing insights on animal movement from existing roadkill data and expert knowledge. Landscape Ecology.

Cavarzere, V., and L. F. Silveira. 2024. Overlooked Bird Extinctions in Semideciduous Atlantic Forests. Ecology and Evolution 14:e70388.

Chase, J. M., S. A. Blowes, T. M. Knight, K. Gerstner, and F. May. 2020. Ecosystem decay exacerbates biodiversity loss with habitat loss. Nature 584:238–243.

Costa, T. R., L. A. da Silva, C. C. de Moura, C. H. de Souto Azevedo, M. L. Bueno, D. P. Mucida, T. Santos, and A. P. D. Gonzaga. 2023. Vulnerability of the Cerrado–Atlantic Forest ecotone in the Espinhaço Range Biosphere Reserve to climate change. Theoretical and Applied Climatology 151:1151–1170.

Culot, L., L. A. Pereira, I. Agostini, M. A. B. Almeida, R. S. C. Alves, I. Aximoff, A. Bager, M. C. Baldovino, T. R. Bella, J. C. Bicca-Marques, C. Braga, C. R. Brocardo, A. K. N. Campelo, G. R. Canale, J. da C. Cardoso, E. Carrano, D. C. Casanova, C. R. Cassano, E. Castro, J. J. Cherem, A. G. Chiarello, B. A. P. Cosenza, R. Costa-Araújo, N. C. da Silva, M. S. Di Bitetti, A. S. Ferreira, P. C. R. Ferreira, M. de S. Fialho, L. F. Fuzessy, G. S. T. Garbino, F. de O. Garcia, C. A. F. R. Gatto, C. C. Gestich, P. R. Gonçalves, N. R. C. Gontijo, M. E. Graipel, C. E. Guidorizzi, R. O. Espíndola Hack, G. P. Hass, R. R. Hilário, A. Hirsch, I. Holzmann, D. H. Homem, H. E. Júnior, G. S. Júnior, M. C. M. Kierulff, C. Knogge, F. Lima, E. F. Lima, C. S. Martins, A. A. Lima, A. Martins, W. P. Martins, F. R. Melo, R. Melzew, J. M. D. Miranda, F. Miranda, A. M. Moraes, T. C. Moreira, M. S. Castro Morini,

M. B. Nagy-Reis, L. Oklander, L. Carvalho Oliveira, A. P. Paglia, A. Pagoto, M. Passamani, F. Camargo Passos, C. A. Peres, M. S. Campos Perine, M. P. Pinto, A. R. M. Pontes, M. Port-Carvalho, B. H. S. do Prado, A. L. Regolin, G. C. Rezende, A. Rocha, J. dos S. Rocha, R. R. Paula Rodarte, L. P. Sales, E. dos Santos, P. M. Santos, C. S. S. Bernardo, R. Sartorello, L. L. Serra, E. Setz, A. S. Almeida e Silva, L. H. da Silva, P. B. E. da Silva, M. Silveira, R. L. Smith, S. M. Souza, A. C. Srbek-Araujo, L. C. Trevelin, C. Valladares-Padua, L. Zago, E. Marques, S. F. Ferrari, R. Beltrão-Mendes, D. J. Henz, F. E. da Veiga da Costa, I. K. Ribeiro, L. L. T. Quintilham, M. Dums, P. M. Lombardi, R. T. R. Bonikowski, S. G. Age, J. P. Souza-Alves, R. Chagas, R. G. T. da Cunha, M. M. Valença-Montenegro, G. Ludwig, L. Jerusalinsky, G. Buss, R. B. Azevedo, R. F. Filho, F. Bufalo, L. Milhe, M. M. dos Santos, R. Sepulvida, D. da S. Ferraz, M. B. Faria, M. C. Ribeiro, and M. Galetti. 2019. ATLANTIC PRIMATES: a dataset of communities and occurrences of primates in the Atlantic Forests of South America. Ecology 100.

Cunha, A. de A., C. B. M. Cruz, and G. A. Bouchardet da Fonseca. 2019. Legal Atlantic Forest (Mata Atlântica Legal): integrating biogeography to public policies towards the conservation of the biodiversity hotspot. Sustentabilidade em Debate 10:320–353.

Da Silva, J. M., and C. H. M. Casteleti. 2003. Status of the Biodiversity of the Atlantic Forest of Brazil. Pages 3–11 *in* C. Galindo-Leal and I. Câmara, editors. Washington (DC): C. Galind-Leal, IG Câmara. The Atlantic Forest of south america: biodiversity status, threats, and outlook. Conservation International.

Dinerstein, E., D. Olson, A. Joshi, C. Vynne, N. D. Burgess, E. Wikramanayake, N. Hahn, S. Palminteri, P. Hedao, R. Noss, M. Hansen, H. Locke, E. C. Ellis, B. Jones, C. V. Barber, R. Hayes, C. Kormos, V. Martin, E. Crist, W. Sechrest, L. Price, J. E. M. Baillie, D. Weeden, K. Suckling, C. Davis, N. Sizer, R. Moore, D. Thau, T. Birch, P. Potapov, S. Turubanova, A. Tyukavina, N. de Souza, L. Pintea, J. C. Brito, O. A. Llewellyn, A. G. Miller, A. Patzelt, S. A. Ghazanfar, J. Timberlake, H. Klöser, Y. Shennan-Farpón, R. Kindt, J.-P. B. Lillesø, P.

van Breugel, L. Graudal, M. Voge, K. F. Al-Shammari, and M. Saleem. 2017. An Ecoregion-Based Approach to Protecting Half the Terrestrial Realm. BioScience 67:534–545.

Diniz, M. F., S. A. Cushman, R. B. Machado, and P. De Marco Júnior. 2020. Landscape connectivity modeling from the perspective of animal dispersal. Landscape Ecology 35:41–58.

Dirzo, R., H. S. Young, M. Galetti, G. Ceballos, N. J. B. Isaac, and B. Collen. 2014. Defaunation in the Anthropocene. Science 345:401–406.

Driscoll, D. A., S. C. Banks, P. S. Barton, D. B. Lindenmayer, and A. L. Smith. 2013. Conceptual domain of the matrix in fragmented landscapes. Trends in Ecology & Evolution 28:605–613.

Duflot, R., A. Ernoult, S. Aviron, L. Fahrig, and F. Burel. 2017. Relative effects of landscape composition and configuration on multi-habitat gamma diversity in agricultural landscapes. Agriculture, Ecosystems & Environment 241:62–69.

Fahrig, L. 2003. Effects of Habitat Fragmentation on Biodiversity. Annual Review of Ecology, Evolution, and Systematics 34:487–515.

Fahrig, L. 2013. Rethinking patch size and isolation effects: the habitat amount hypothesis. Journal of Biogeography 40:1649–1663.

Fahrig, L. 2017. Ecological responses to habitat fragmentation per se. Annual Review of Ecology, Evolution, and Systematics 48:1–23.

Feitosa, R. M., M. S. de Castro Morini, A. C. Martins, T. M. de Andrade Ribeiro, F. B. Noll, E. F. dos Santos, E. M. Cancello, and J. P. Constantini. 2021. Social Insects of the Atlantic Forest. Pages 151–183 *in* M. C. M. Marques and C. E. V. Grelle, editors. The Atlantic Forest: History, Biodiversity, Threats and Opportunities of the Mega-diverse Forest. Springer International Publishing, Cham.

Ferro e Silva, A. M., T. Sobral-Souza, M. H. Vancine, R. L. Muylaert, A. P. de Abreu, S. M.

Pelloso, M. D. de Barros Carvalho, L. de Andrade, M. C. Ribeiro, and M. J. de O. Toledo. 2018. Spatial prediction of risk areas for vector transmission of Trypanosoma cruzi in the State of Paraná, southern Brazil. PLOS Neglected Tropical Diseases 12:e0006907.

Fick, S. E., and R. J. Hijmans. 2017. WorldClim 2: new 1-km spatial resolution climate surfaces for global land areas. International Journal of Climatology 37:4302–4315.

Figueiredo, M. de S. L., M. M. Weber, C. A. Brasileiro, R. Cerqueira, C. E. V. Grelle, C. N. Jenkins, C. V. Solidade, M. T. C. Thomé, M. M. Vale, and M. L. Lorini. 2021. Tetrapod Diversity in the Atlantic Forest: Maps and Gaps. Pages 185–204 *in* M. C. M. Marques and C. E. V. Grelle, editors. The Atlantic Forest. Springer International Publishing, Cham.

Figueiredo, M. S. L., C. S. Barros, A. C. Delciellos, E. B. Guerra, P. Cordeiro-Estrela, M. Kajin, M. R. Alvarez, P. H. Asfora, D. Astúa, H. G. Bergallo, R. Cerqueira, L. Geise, R. Gentile, C. E. V. Grelle, G. E. Iack-Ximenes, L. C. Oliveira, M. Weksler, and M. V. Vieira. 2017. Abundance of small mammals in the Atlantic Forest (ASMAF): a data set for analyzing tropical community patterns. Ecology 98:2981–2981.

Fischer, J., and D. B. Lindenmayer. 2007. Landscape modification and habitat fragmentation: a synthesis. Global Ecology and Biogeography 16:265–280.

Flora e Funga do Brasil. Jardim Botânico do Rio de Janeiro. Available at: http://floradobrasil.jbrj.gov.br. Accessed on: 10 Oct 2023.

Fletcher, R., and M.-J. Fortin. 2018. Spatial Ecology and Conservation Modeling: Applications with R. Springer International Publishing, Cham.

Fletcher, R. J., R. A. McCleery, D. U. Greene, and C. A. Tye. 2016. Integrated models that unite local and regional data reveal larger-scale environmental relationships and improve predictions of species distributions. Landscape Ecology 31:1369–1382.

Forman, R. T. T., and M. Godron. 1986. Landscape ecology. Wiley, New York, NY.

Franceschi, I. C., R. A. da P. Dornas, I. S. Lermen, A. V. P. Coelho, A. H. Vilas Boas, A. G. Chiarello, A. P. Paglia, A. C. de Souza, A. R. Borsekowsky, A. Rocha, A. Bager, A. Z. de Souza, A. M. C. Lopes, A. S. de Moura, A. S. Ferreira, A. García-Olaechea, A. C.

Delciellos, A. E. de F. Bacellar, A. K. N. Campelo, A. M. O. Paschoal, A. C. Rolim, A. L. F. da Silva, A. M. Lanna, A. P. da Silva, A. Guimarães, Â. Cardoso, A. S. Cassol, A. L. da Costa-Pinto, A. G. S. do Nascimento, A. S. Fernandes, A. Clyvia, A. B. dos Santos, B. Lima-Silva, B. de M. Beisiegel, B. F. L. Luciano, B. de F. Leopoldo, B. N. Krobel, B. B. Kubiak, B. H. Saranholi, B. S. Correa, C. Sant Anna Teixeira, C. R. Ayroza, C. R. Cassano, C. Benitez-Riveros, C. C. Gestich, C. D. Tedesco, C. Gheler-Costa, C. G. Z. Hegel, C. da S. Evangelista Junior, C. E. M. F. Ferreira, C. E. V. Grelle, C. F. Esteves, C. da C. Espinosa, C. Leuchtenberger, C. Sanchéz-Lalinde, C. I. C. Machado, C. Andreazzi, C. Bueno, C. Cronemberger de Faria, C. Novaes, C. E. Widmer, C. C. Santos, D. da S. Ferraz, D. Galiano, D. A. S. Bôlla, D. Behs, D. P. Rodrigues, D. P. de Melo, D. M. S. Ramos, D. L. de Mattia, D. D. Pavei, D. Loretto, D. da S. Huning, D. de M. Dias, É. R. Paetzhold, E. Rios, E. Z. F. Setz, E. Cazetta, E. G. Cafofo Silva, E. Pasa, E. N. Saito, E. F. S. de Aguiar, É. P. Castro, E. B. Viveiros de Castro, E. Pedó, F. de A. Pereira, F. Bolzan, F. de O. Roque, F. D. Mazim, F. H. Comin, F. Maffei, F. B. Peters, F. M. Fantacini, F. P. da Silva, F. S. Machado, F. Vélez-Garcia, F. S. D. Lage, F. A. Perini, F. C. Passos, F. Carvalho, F. C. C. de Azevedo, F. Ferreira, F. F. de Pinho, F. G. Chaves, F. R. Miranda, F. H. G. Rodrigues, F. K. Ubaid, F. H. Gabriel, F. L. de Souza, F. V. de Oliveira, G. Cupolillo, G. de A. P. Moreira, G. Mette, G. T. Duarte, G. Beca, G. Corso, G. Perbiche-Neves, G. H. B. de O. Souto, G. J. da S. Vilarroel, G. O. Batista, G. B. Ferreira, G. A. da C. Toledo, G. Senger, H. de G. Bergallo, H. C. P. dos Santos, H. A. Gazola, I. Melo, I. V. Brack, I. Veríssimo, I. R. Viana, I. C. Laurentino, J. L. Diehl, J. J. Zocche, J. Martins-Silva, J. P. G. Just, J. J. Cherem, J. L. Nascimento, J. R. Marinho, J. O. Dantas, J. R. de Matos, J. S. R. Pires, J. F. Cerveira, J. Ruiz-Esparza, J. P. da Silva, J. A. Bogoni, K. T. Molina, K. D. de L. Pereira, K. Ceron, K. de Vleeschouwer, L. Lautenschlager, L. Bailey, L. Fornitano, L. E. Rampim, L. Sforza, L. G. Bissa, L. M. Santucci, L. G. da Silva, L. N. Perillo, L. R. Correa, L. Hufnagel, L. F. Alberti, L. J. Recalde Mello, L. R. R. Bernardo, L. G. R. Oliveira-Santos, L. N. Guimarães,

M. Benchimol, M. C. Twardowschy, M. Ferreira-Riveros, M. da Silva, M. M. de A. Jardim, M. A. L. Fontes, M. A. Tortato, M. T. do Nascimento, M. L. Sekiama, M. C. Nascimento-Costa, M. E. B. dos Santos, M. S. de C. Morini, M. B. Nagy-Reis, M. da C. Kaizer, M. J. R. da S. Sant'Anna, M. T. Hartmann, M. O. Favarini, M. O. Olivo, M. A. Montes, M. R. del V. Alvaréz, M. F. Haddad, M. D. Costa, M. E. Graipel, M. Q. Konzen, M. Galetti, M. de O. S. Almeida, M. B. Faria, M. R. Luiz, M. N. da M. Baptista, M. Â. Marini, M. C. Ribeiro, N. Olifiers, N. M. de Albuquerque, N. Cantero, N. Peroni, N. Zanella, O. Mendonça-Furtado, O. Pays, O. E. Ferretti, O. Rocha-Barbosa, P. M. Santos, P. M. de Farias, P. A. da Rocha, P. F. Colas-Rosas, P. Ribeiro-Souza, P. Ferracioli, P. A. Hartmann, P. de T. Z. Antas, P. Ribeiro, P. Tomasi Sarti, P. I. Mônico, P. V. de Castilho, P. B. de M. Pereira, P. G. Crawshaw Jr, P.-C. Renaud, R. S. Romagna, R. T. M. de Sousa, R. S. Spagnol, R. Beltrão-Mendes, R. F. Mariano, R. R. Rocha, R. Sousa-Lima, R. V. Pagotto, R. T. de Faria, R. C. Arrais, R. Moratelli, R. Sartorello, R. de C. Bianchi, R. de C. Guimarães, R. L. Massara, R. T. Costa, R. V. Marques, R. M. R. Nunes, S. M. Hartz, S. M. Silvestre de Sousa, S. R. Lima, S. L. Barbosa, S. N. Godoy, S. F. Ferrari, T. G. de Araújo-Piovezan, T. L. Góes, T. C. Trigo, T. R. O. de Freitas, T. B. Maccarini, T. M. de Castro, T. R. Bella, T. M. de Oliveira Junior, U. M. Cunha, V. T. Kanaan, V. Pfannerstill, V. S. Pimentel, V. Picinatto Filho, V. N. Alves, V. Rojas-Bonzi, V. Mottin, V. J. Rocha, A. Kindel, and I. P. Coelho. 2024. Camera trap surveys of Atlantic Forest mammals: A data set for analyses considering imperfect detection (2004–2020). Ecology 105:e4298.

Galetti, M., C. R. Brocardo, R. A. Begotti, L. Hortenci, F. Rocha-Mendes, C. S. S. Bernardo, R. S. Bueno, R. Nobre, R. S. Bovendorp, R. M. Marques, F. Meirelles, S. K. Gobbo, G. Beca, G. Schmaedecke, and T. Siqueira. 2017. Defaunation and biomass collapse of mammals in the largest Atlantic forest remnant. Animal Conservation 20:270–281.

Galetti, M., F. Gonçalves, N. Villar, V. B. Zipparro, C. Paz, C. Mendes, L. Lautenschlager, Y. Souza, P. Akkawi, F. Pedrosa, L. Bulascoschi, C. Bello, A. P. Sevá, L. Sales, L. Genes, F.

Abra, and R. S. Bovendorp. 2021. Causes and Consequences of Large-Scale Defaunation in the Atlantic Forest. Pages 297–324 *in* M. C. M. Marques and C. E. V. Grelle, editors. The Atlantic Forest: History, Biodiversity, Threats and Opportunities of the Mega-diverse Forest. Springer International Publishing, Cham.

Giupponi, A., P. Demite, C. Flechtmann, F. Hernandes, A. Mendes, G. H. Migliorini, G. Miranda, and T. Gonçalves-Souza. 2017. Aracnídeos da Mata Atlântica. Pages 129–235 *in* E. L. A. M. Filho and C. E. Conte, editors. Revisões em zoologia: Mata Atlântica. Editora UFPR, Curitiba, Paraná, Brasil.

Gonçalves, F., R. S. Bovendorp, G. Beca, C. Bello, R. Costa-Pereira, R. L. Muylaert, R. R. Rodarte, N. Villar, R. Souza, M. E. Graipel, J. J. Cherem, D. Faria, J. Baumgarten, M. R. Alvarez, E. M. Vieira, N. Cáceres, R. Pardini, Y. L. R. Leite, L. P. Costa, M. A. R. Mello, E. Fischer, F. C. Passos, L. H. Varzinczak, J. A. Prevedello, A. P. Cruz-Neto, F. Carvalho, A. R. Percequillo, A. Paviolo, A. Nava, J. M. B. Duarte, N. U. de la Sancha, E. Bernard, R. G. Morato, J. F. Ribeiro, R. G. Becker, G. Paise, P. S. Tomasi, F. Vélez-Garcia, G. L. Melo, J. Sponchiado, F. Cerezer, M. A. S. Barros, A. Q. S. de Souza, C. C. dos Santos, G. A. F. Giné, P. Kerches-Rogeri, M. M. Weber, G. Ambar, L. V. Cabrera-Martinez, A. Eriksson, M. Silveira, C. F. Santos, L. Alves, E. Barbier, G. C. Rezende, G. S. T. Garbino, É. O. Rios, A. Silva, A. T. A. Nascimento, R. S. de Carvalho, A. Feijó, J. Arrabal, I. Agostini, D. Lamattina, S. Costa, E. Vanderhoeven, F. R. de Melo, P. de Oliveira Laroque, L. Jerusalinsky, M. M. Valença-Montenegro, A. B. Martins, G. Ludwig, R. B. de Azevedo, A. Anzóategui, M. X. da Silva, M. Figuerêdo Duarte Moraes, A. Vogliotti, A. Gatti, T. Püttker, C. S. Barros, T. K. Martins, A. Keuroghlian, D. P. Eaton, C. L. Neves, M. S. Nardi, C. Braga, P. R. Gonçalves, A. C. Srbek-Araujo, P. Mendes, J. A. de Oliveira, F. A. M. Soares, P. A. Rocha, P. Crawshaw, M. C. Ribeiro, and M. Galetti. 2018a. ATLANTIC MAMMAL TRAITS: a data set of morphological traits of mammals in the Atlantic Forest of South America. Ecology 99:498–498.

Gonçalves, F., W. Hannibal, M. N. Godoi, F. I. Martins, R. F. Oliveira, V. V. Figueiredo, J. Casella, and É. F. G. G. de Sá. 2018b. Non-volant mammals from the Upper Paraná River Basin: a data set from a critical region for conservation in Brazil. Ecology 99:499–499.

Gonçalves-Souza, T., J. M. Chase, N. M. Haddad, M. H. Vancine, R. K. Didham, F. L. P. Melo, M. A. Aizen, E. Bernard, A. G. Chiarello, D. Faria, H. Gibb, M. G. de Lima, L. F. S. Magnago, E. Mariano-Neto, A. A. Nogueira, A. Nemésio, M. Passamani, B. X. Pinho, L. Rocha-Santos, R. C. Rodrigues, N. V. H. Safar, B. A. Santos, A. Soto-Werschitz, M. Tabarelli, M. Uehara-Prado, H. L. Vasconcelos, S. Vieira, and N. J. Sanders. 2025a. Species turnover does not rescue biodiversity in fragmented landscapes. Nature 640:702–706.

Gonçalves-Souza, T., M. H. Vancine, N. J. Sanders, N. M. Haddad, L. Cortinhas, A. L. T. O. Aase, W. M. de Aguiar, M. A. Aizen, V. Arroyo-Rodríguez, A. Baz, M. Benchimol, E. Bernard, T. J. Bertotto, A. A. Bispo, J. A. Bogoni, G. X. Boldorini, C. Bragagnolo, B. Brosi, A. S. Cantalice, R. F. R. do Carmo, E. Cazeta, A. G. Chiarello, N. U. de la Sancha, R. K. Didham, D. Faria, B. Filgueiras, J. E. C. Figueira, G. A. Galvão, M. V. Garey, H. Gibb, C. Gómez-Martínez, E. González, R. A. F. de Gusmão, M. Henry, S. de Jesus, T. G. Kloss, A. Lázaro, V. Leandro-Silva, M. G. de Lima, I. da Silva Lima, A. C. B. Lins-e-Silva, R. M. Nally, A. R. Magalhães, L. F. S. Magnago, S. Manu, E. Mariano-Neto, D. N. M. Mbora, F. P. L. Melo, M. N. Mutua, S. Neckel-Oliveira, A. Nemésio, A. A. Nogueira, P. M. D. A. Oliveira, D. G. Pádua, L. Paes, A. B. de Paiva, M. Passamani, J. C. Pena, C. A. Peres, B. X. Pinho, J.-M. Pons, V. M. Prasniewski, J. Reiniö, M. dos Santos Rocha, L. Rocha-Santos, M. J. Rodal, R. C. Rodrigues, N. V. H. Safar, R. P. Salomão, B. A. Santos, M. N. Santos, J. P. dos Santos, S. Savilaakso, C. E. G. R. Schaefer, M. A. M. Silva, F. R. da Silva, R. J. Silva, M. Simonelli, A. Soto-Werschitz, J. O. Stireman III, D. Storck-Tonon, N. Szinwelski, M. Tabarelli, C. P. Teixeira, Ø. Totland, M. Uehara-Prado, F. Z. Vaz-de-Mello, H. L. Vasconcelos, S. A. Vieira, and J. M. Chase. 2025b. LandFrag: A Dataset to Investigate the Effects of Forest Loss and Fragmentation on Biodiversity. Global Ecology and

Biogeography 34:e70015.

Haddad, N. M., L. A. Brudvig, J. Clobert, K. F. Davies, A. Gonzalez, R. D. Holt, T. E. Lovejoy, J. O. Sexton, M. P. Austin, C. D. Collins, W. M. Cook, E. I. Damschen, R. M. Ewers, B. L. Foster, C. N. Jenkins, A. J. King, W. F. Laurance, D. J. Levey, C. R. Margules, B. A. Melbourne, A. O. Nicholls, J. L. Orrock, D.-X. Song, and J. R. Townshend. 2015. Habitat fragmentation and its lasting impact on Earth's ecosystems. Science Advances 1:e1500052.

Hagen-Zanker, A. 2016. A computational framework for generalized moving windows and its application to landscape pattern analysis. International Journal of Applied Earth Observation and Geoinformation 44:205–216.

Hansen, M. C., P. V. Potapov, A. H. Pickens, A. Tyukavina, A. Hernandez-Serna, V. Zalles, S. Turubanova, I. Kommareddy, S. V. Stehman, X.-P. Song, and A. Kommareddy. 2022. Global land use extent and dispersion within natural land cover using Landsat data. Environmental Research Letters 17:034050.

Harper, K. A., and S. E. Macdonald. 2011. Quantifying distance of edge influence: a comparison of methods and a new randomization method. Ecosphere 2:art94.

Harper, K. A., S. E. Macdonald, P. J. Burton, J. Chen, K. D. Brosofske, S. C. Saunders, E. S. Euskirchen, D. Roberts, M. S. Jaiteh, and P.-A. Esseen. 2005. Edge Influence on Forest Structure and Composition in Fragmented Landscapes. Conservation Biology 19:768–782.

Harper, K. A., J. R. Yang, N. Dazé Querry, J. Dyer, R. S. C. Alves, and M. C. Ribeiro. 2024. Limited influence from edges and topography on vegetation structure and diversity in Atlantic Forest. Plant Ecology 225:361–371.

Hasui, É., J. P. Metzger, R. G. Pimentel, L. F. Silveira, A. A. d. A. Bovo, A. C. Martensen, A. Uezu, A. L. Regolin, A. Â. Bispo de Oliveira, C. A. F. R. Gatto, C. Duca, C. B. Andretti, C. Banks-Leite, D. Luz, D. Mariz, E. R. Alexandrino, F. M. de Barros, F. Martello, I. M. d. S. Pereira, J. N. da Silva, K. M. P. M. d. B. Ferraz, L. N. Naka, L. dos Anjos, M. A. Efe, M. A. Pizo, M. Pichorim, M. S. S. Gonçalves, P. H. C. Cordeiro, R. A. Dias, R. d. L. Muylaert, R.

C. Rodrigues, T. V. V. da Costa, V. Cavarzere, V. R. Tonetti, W. R. Silva, C. N. Jenkins, M. Galetti, and M. C. Ribeiro. 2018. ATLANTIC BIRDS: a data set of bird species from the Brazilian Atlantic Forest. Ecology 99:497–497.

Hatfield, J. H., C. D. L. Orme, and C. Banks-Leite. 2018. Using functional connectivity to predict potential meta-population sizes in the Brazilian Atlantic Forest. Perspectives in Ecology and Conservation 16:215–220.

Hawker, L., P. Uhe, L. Paulo, J. Sosa, J. Savage, C. Sampson, and J. Neal. 2022. A 30 m global map of elevation with forests and buildings removed. Environmental Research Letters 17:024016.

He, K. S., B. A. Bradley, A. F. Cord, D. Rocchini, M. Tuanmu, S. Schmidtlein, W. Turner, M. Wegmann, and N. Pettorelli. 2015. Will remote sensing shape the next generation of species distribution models? Remote Sensing in Ecology and Conservation 1:4–18.

He, X., J. Liang, G. Zeng, Y. Yuan, and X. Li. 2019. The Effects of Interaction between Climate Change and Land-Use/Cover Change on Biodiversity-Related Ecosystem Services. Global Challenges 3:1800095.

Hofierka, J., H. Mitášová, and M. Neteler. 2009. Chapter 17 Geomorphometry in GRASS GIS. Pages 387–410 Developments in Soil Science. Elsevier.

Holmgren, P. 1994. Multiple flow direction algorithms for runoff modelling in grid based elevation models: An empirical evaluation. Hydrological Processes 8:327–334.

IBGE — Instituto Brasileiro de Geografia e Estatística, 2019. Biomas e Sistema Costeiro-Marinho do Brasil — 1:250000. Available at: https://www.ibge.gov.br/geociencias/cartas-e-mapas/informacoes-ambientais/15842-biomas.html?=&t=acesso-ao-produto.

Iezzi, M. E., M. S. Di Bitetti, J. Martínez Pardo, A. Paviolo, P. Cruz, and C. De Angelo. 2022. Forest fragments prioritization based on their connectivity contribution for multiple Atlantic Forest mammals. Biological Conservation 266:109433.

IGN — Instituto Geográfico Nacional, 2022. Available at: https://www.ign.gob.ar.

INE — Instituto Nacional de Estadística, 2022. Available at: https://www.ine.gov.py.

Iserhard, C., M. Uehara-Prado, O. Marini-Filho, M. Duarte, and A. Freitas. 2017. Fauna da Mata

Atlântica: Lepidoptera-Borboletas. Pages 57–102 *in* E. L. de A. Monteiro-Filho and C. E. Conte, editors. Revisões em zoologia: Mata Atlântica. Editora UFPR, Curitiba, Paraná, Brasil.

Jackson, H. B., and L. Fahrig. 2015. Are ecologists conducting research at the optimal scale?: Is research conducted at optimal scales? Global Ecology and Biogeography 24:52–63.

Jasiewicz, J., and T. F. Stepinski. 2013. Geomorphons — a pattern recognition approach to classification and mapping of landforms. Geomorphology 182:147–156.

Jetz, W., D. S. Wilcove, and A. P. Dobson. 2007. Projected Impacts of Climate and Land-Use Change on the Global Diversity of Birds. PLOS Biology 5:e157.

Johnson, C. N., A. Balmford, B. W. Brook, J. C. Buettel, M. Galetti, L. Guangchun, and J. M. Wilmshurst. 2017. Biodiversity losses and conservation responses in the Anthropocene. Science 356:270–275.

Joly, C. A., J. P. Metzger, and M. Tabarelli. 2014. Experiences from the Brazilian Atlantic Forest: ecological findings and conservation initiatives. New Phytologist 204:459–473.

Kareiva, P. 1994. Special Feature: Space: The Final Frontier for Ecological Theory. Ecology 75:1–1.

Karger, D. N., D. R. Schmatz, G. Dettling, and N. E. Zimmermann. 2020. High-resolution monthly precipitation and temperature time series from 2006 to 2100. Scientific Data 7:248.

Koen, E. L., E. H. Ellington, and J. Bowman. 2019. Mapping landscape connectivity for large spatial extents. Landscape Ecology 34:2421–2433.

Lehner, B., and G. Grill. 2013. Global river hydrography and network routing: baseline data and new approaches to study the world's large river systems: GLOBAL RIVER HYDROGRAPHY AND NETWORK ROUTING. Hydrological Processes 27:2171–2186.

Leite, I. B. 2015. The Brazilian quilombo: 'race', community and land in space and time. The Journal of Peasant Studies 42:1225–1240.

Lembi, R. C., C. Cronemberger, C. Picharillo, S. Koffler, P. H. A. Sena, J. F. Felappi, A. R. de

Moraes, A. Arshad, J. P. dos Santos, and A. V. Mansur. 2020. Urban expansion in the Atlantic Forest: applying the Nature Futures Framework to develop a conceptual model and future scenarios. Biota Neotropica 20:e20190904.

Libohova, Z., H. E. Winzeler, B. Lee, P. J. Schoeneberger, J. Datta, and P. R. Owens. 2016. Geomorphons: Landform and property predictions in a glacial moraine in Indiana landscapes. CATENA 142:66–76.

Lima, F., G. Beca, R. L. Muylaert, C. N. Jenkins, M. L. L. Perilli, A. M. O. Paschoal, R. L. Massara, A. P. Paglia, A. G. Chiarello, M. E. Graipel, J. J. Cherem, A. L. Regolin, L. G. R. Oliveira Santos, C. R. Brocardo, A. Paviolo, M. S. Di Bitetti, L. M. Scoss, F. L. Rocha, R. Fusco-Costa, C. A. Rosa, M. X. Da Silva, L. Hufnagell, P. M. Santos, G. T. Duarte, L. N. Guimarães, L. L. Bailey, F. H. G. Rodrigues, H. M. Cunha, F. M. Fantacini, G. O. Batista, J. A. Bogoni, M. A. Tortato, M. R. Luiz, N. Peroni, P. V. De Castilho, T. B. Maccarini, V. P. Filho, C. D. Angelo, P. Cruz, V. Quiroga, M. E. Iezzi, D. Varela, S. M. C. Cavalcanti, A. C. Martensen, E. V. Maggiorini, F. F. Keesen, A. V. Nunes, G. M. Lessa, P. Cordeiro-Estrela, M. G. Beltrão, A. C. F. De Albuquerque, B. Ingberman, C. R. Cassano, L. C. Junior, M. C. Ribeiro, and M. Galetti. 2017. ATLANTIC-CAMTRAPS: a dataset of medium and large terrestrial mammal communities in the Atlantic Forest of South America. Ecology 98:2979–2979.

de Lima, R. A. F., G. Dauby, A. L. de Gasper, E. P. Fernandez, A. C. Vibrans, A. A. de Oliveira, P. I. Prado, V. C. Souza, M. F. de Siqueira, and H. ter Steege. 2024. Comprehensive conservation assessments reveal high extinction risks across Atlantic Forest trees. Science 383:219–225.

de Lima, R. A. F., A. A. Oliveira, G. R. Pitta, A. L. de Gasper, A. C. Vibrans, J. Chave, H. ter Steege, and P. I. Prado. 2020. The erosion of biodiversity and biomass in the Atlantic Forest biodiversity hotspot. Nature Communications 11:6347.

Lima-Ribeiro, M. S., S. Varela, J. González-Hernández, G. de Oliveira, J. A. F. Diniz-Filho, and L.

C. Terribile. 2015. EcoClimate: a database of climate data from multiple models for past, present, and future for macroecologists and biogeographers. Biodiversity Informatics 10:1–21.

Lira, P. K., R. de C. Q. Portela, and L. R. Tambosi. 2021. Land-Cover Changes and an Uncertain Future: Will the Brazilian Atlantic Forest Lose the Chance to Become a Hopespot? Pages 233–251 *in* M. C. M. Marques and C. E. V. Grelle, editors. The Atlantic Forest: History, Biodiversity, Threats and Opportunities of the Mega-diverse Forest. Springer International Publishing, Cham.

Lopes, B. S., K. A. B. Corrêa, M. E. K. Ogasawara, R. S. Precinoto, C. C. Cassiano, B. M. Sell, R. S. Melo, P. C. dos Reis Oliveira, and S. F. de B. Ferraz. 2022. How does land use cover change affect hydrological response in the Atlantic Forest? Implications for ecological restoration. Frontiers in Water 4:998349.

Ma, J., J. Li, W. Wu, and J. Liu. 2023. Global forest fragmentation change from 2000 to 2020. Nature Communications 14:3752.

MapBiomas Project — Collection 7 of the Annual Series of Land Cover and Use Maps of Brazil, 2022. Accessed on 01/11/2022, through the link: https://brasil.mapbiomas.org.

MapBiomas Trinational Atlantic Forest Project — Collection 2 of the Annual Series of Land Use and Cover Maps of the Trinational Atlantic Forest, 2022. Accessed on 01/11/2022, through the link: https://bosqueatlantico.mapbiomas.org.

Marjakangas, E., N. Abrego, V. Grøtan, R. A. F. Lima, C. Bello, R. S. Bovendorp, L. Culot, É. Hasui, F. Lima, R. L. Muylaert, B. B. Niebuhr, A. A. Oliveira, L. A. Pereira, P. I. Prado, R. D. Stevens, M. H. Vancine, M. C. Ribeiro, M. Galetti, and O. Ovaskainen. 2020. Fragmented tropical forests lose mutualistic plant–animal interactions. Diversity and Distributions 26:154–168.

Marques, M. C. M., W. Trindade, A. Bohn, and C. E. V. Grelle. 2021. The Atlantic Forest: An Introduction to the Megadiverse Forest of South America. Pages 3–23 *in* M. C. M. Marques and C. E. V. Grelle, editors. The Atlantic Forest: History, Biodiversity, Threats and Opportunities of the Mega-diverse Forest. Springer International Publishing, Cham.

Martinez Pardo, J., S. Saura, A. Insaurralde, M. S. Di Bitetti, A. Paviolo, and C. De Angelo. 2023. Much more than forest loss: four decades of habitat connectivity decline for Atlantic Forest jaguars. Landscape Ecology 38:41–57.

McGarigal K., S. A. Cushman, and E. Ene. 2023. FRAGSTATS v4: Spatial Pattern Analysis Program for Categorical Maps. Available at: https://www.fragstats.org.

Melo, F. P. L., S. R. R. Pinto, P. H. S. Brancalion, P. S. Castro, R. R. Rodrigues, J. Aronson, and M. Tabarelli. 2013. Priority setting for scaling-up tropical forest restoration projects: Early lessons from the Atlantic Forest Restoration Pact. Environmental Science & Policy 33:395–404.

Messager, M. L., B. Lehner, G. Grill, I. Nedeva, and O. Schmitt. 2016. Estimating the volume and age of water stored in global lakes using a geo-statistical approach. Nature Communications 7:13603.

Messager, M. L., J. D. Olden, J. D. Tonkin, R. Stubbington, J. S. Rogosch, M. H. Busch, C. J. Little, A. W. Walters, C. L. Atkinson, M. Shanafield, S. Yu, K. S. Boersma, D. A. Lytle, R. H. Walker, R. M. Burrows, and T. Datry. 2023. A metasystem approach to designing environmental flows. BioScience 73:643–662.

Miguet, P., H. B. Jackson, N. D. Jackson, A. E. Martin, and L. Fahrig. 2016. What determines the spatial extent of landscape effects on species? Landscape Ecology 31:1177–1194.

Mimet, A., T. Houet, R. Julliard, and L. Simon. 2013. Assessing functional connectivity: a landscape approach for handling multiple ecological requirements. Methods in Ecology and Evolution 4:453–463.

Monteiro, E. C. S., M. A. Pizo, M. H. Vancine, and M. C. Ribeiro. 2022. Forest cover and connectivity have pervasive effects on the maintenance of evolutionary distinct interactions in seed dispersal networks. Oikos 2022:oik.08240.

Muylaert, R. L., R. D. Stevens, C. E. L. Esbérard, M. A. R. Mello, G. S. T. Garbino, L. H. Varzinczak, D. Faria, M. d. M. Weber, P. Kerches Rogeri, A. L. Regolin, H. F. M. d. Oliveira, L. d. M. Costa, M. A. S. Barros, G. Sabino-Santos, M. A. Crepaldi de Morais, V.

S. Kavagutti, F. C. Passos, E.-L. Marjakangas, F. G. M. Maia, M. C. Ribeiro, and M. Galetti. 2017. ATLANTIC BATS: a data set of bat communities from the Atlantic Forests of South America. Ecology 98:3227–3227.

Muylaert, R. L., M. H. Vancine, R. Bernardo, J. E. F. Oshima, T. Sobral-Souza, V. R. Tonetti, B. B. Niebuhr, and M. C. Ribeiro. 2018. Uma nota sobre os limites territoriais da Mata Atlântica. Oecologia Australis 22:302–311.

Muylaert, R. L., D. A. Wilkinson, E. I. Dwiyanti, and D. T. S. Hayman. 2025. Upscaling effects on infectious disease emergence risk emphasize the need for local planning in primary prevention within biodiversity hotspots. Scientific Reports 15:37504.

Myers, N., R. A. Mittermeier, C. G. Mittermeier, G. A. B. da Fonseca, and J. Kent. 2000. Biodiversity hotspots for conservation priorities. Nature 403:853–858.

Neteler, M., M. H. Bowman, M. Landa, and M. Metz. 2012. GRASS GIS: A multi-purpose open source GIS. Environmental Modelling & Software 31:124–130.

Niebuhr, B. B., B. Van Moorter, A. Stien, T. Tveraa, O. Strand, K. Langeland, P. Sandström, M. Alam, A. Skarin, and M. Panzacchi. 2023. Estimating the cumulative impact and zone of influence of anthropogenic features on biodiversity. Methods in Ecology and Evolution 14:2362–2375.

Niebuhr, B. B., F. Martello, J. Wesley Ribeiro, M. Humberto Vancine, R. de Lara Muylaert, V. E. W. Campos, J. Silveira dos Santos, V. R. Tonetti, and Milton Cezar Ribeiro. 2020. Landscape Metrics (LSMetrics): A tool for calculating landscape connectivity and other ecologically scaled landscape metrics (v0.91). Zenodo. https://doi.org/10.5281/zenodo.3736444

Okabe, A., T. Satoh, and K. Sugihara. 2009. A kernel density estimation method for networks, its computational method and a GIS-based tool. International Journal of Geographical Information Science 23:7–32.

Oshima, J. E. de F., M. L. S. P. Jorge, T. Sobral-Souza, L. Börger, A. Keuroghlian, C. A. Peres, M. H. Vancine, B. Collen, and M. C. Ribeiro. 2021. Setting priority conservation management regions to reverse rapid range decline of a key neotropical forest ungulate. Global Ecology

and Conservation 31:e01796.

Palmeirim, A. F., M. S. L. Figueiredo, C. E. V. Grelle, C. Carbone, and M. V. Vieira. 2019. When does habitat fragmentation matter? A biome-wide analysis of small mammals in the Atlantic Forest. Journal of Biogeography 46:2811–2825.

Peres, E. A., R. Pinto-da-Rocha, L. G. Lohmann, F. A. Michelangeli, C. Y. Miyaki, and A. C. Carnaval. 2020. Patterns of Species and Lineage Diversity in the Atlantic Rainforest of Brazil. Pages 415–447 *in* V. Rull and A. C. Carnaval, editors. Neotropical Diversification: Patterns and Processes. Springer International Publishing, Cham.

Piffer, P. R., A. Calaboni, M. R. Rosa, N. B. Schwartz, L. R. Tambosi, and M. Uriarte. 2022. Ephemeral forest regeneration limits carbon sequestration potential in the Brazilian Atlantic Forest. Global Change Biology 28:630–643.

Pinto, S. R., F. Melo, M. Tabarelli, A. Padovesi, C. A. Mesquita, C. A. De Mattos Scaramuzza, P. Castro, H. Carrascosa, M. Calmon, R. Rodrigues, R. G. César, and P. H. S. Brancalion. 2014. Governing and Delivering a Biome-Wide Restoration Initiative: The Case of Atlantic Forest Restoration Pact in Brazil. Forests 5:2212–2229.

Pires, A. P. F., C. Y. Shimamoto, M. C. G. Padgurschi, F. R. Scarano, and M. C. M. Marques. 2021. Atlantic Forest: Ecosystem Services Linking People and Biodiversity. Pages 347–367 *in* M. C. M. Marques and C. E. V. Grelle, editors. The Atlantic Forest. Springer International Publishing, Cham.

Poggio, L., L. M. de Sousa, N. H. Batjes, G. B. M. Heuvelink, B. Kempen, E. Ribeiro, and D. Rossiter. 2021. SoilGrids 2.0: producing soil information for the globe with quantified spatial uncertainty. SOIL 7:217–240.

Potapov, P., M. C. Hansen, A. Pickens, A. Hernandez-Serna, A. Tyukavina, S. Turubanova, V. Zalles, X. Li, A. Khan, F. Stolle, N. Harris, X.-P. Song, A. Baggett, I. Kommareddy, and A. Kommareddy. 2022a. The Global 2000-2020 Land Cover and Land Use Change Dataset Derived From the Landsat Archive: First Results. Frontiers in Remote Sensing 3.

Potapov, P., S. Turubanova, M. C. Hansen, A. Tyukavina, V. Zalles, A. Khan, X.-P. Song, A. Pickens, Q. Shen, and J. Cortez. 2022b. Global maps of cropland extent and change show accelerated cropland expansion in the twenty-first century. Nature Food 3:19–28.

Pyles, M. V., L. F. S. Magnago, V. A. Maia, B. X. Pinho, G. Pitta, A. L. de Gasper, A. C. Vibrans, R. M. dos Santos, E. van den Berg, and R. A. F. Lima. 2022. Human impacts as the main driver of tropical forest carbon. Science Advances 8:eabl7968.

Ramos, F. N., S. R. Mortara, N. Monalisa-Francisco, J. P. C. Elias, L. M. Neto, L. Freitas, R. Kersten, A. M. Amorim, F. B. Matos, A. F. Nunes-Freitas, S. Alcantara, M. H. N. Alexandre, R. J. Almeida-Scabbia, O. J. G. Almeida, F. E. Alves, R. M. Oliveira Alves, F. S. Alvim, A. C. S. Andrade, S. Andrade, L. Y. S. Aona, A. C. Araujo, K. C. T. Araújo, V. Ariati, J. C. Assis, C. O. Azevedo, B. F. Barbosa, D. E. F. Barbosa, F. dos R. Barbosa, F. Barros, G. A. Basilio, F. A. Bataghin, F. Bered, J. S. Bianchi, C. T. Blum, C. R. Boelter, A. Bonnet, P. H. S. Brancalion, T. B. Breier, C. de T. Brion, C. R. Buzatto, A. Cabral, T. J. Cadorin, E. Caglioni, L. Canêz, P. H. Cardoso, F. S. Carvalho, R. G. Carvalho, E. L. M. Catharino, S. J. Ceballos, M. T. Cerezini, R. G. César, C. Cestari, C. J. N. Chaves, V. Citadini-Zanette, L. F. M. Coelho, J. V. Coffani-Nunes, R. Colares, G. D. Colletta, N. de M. Corrêa, A. F. Costa, G. M. Costa, L. M. S. Costa, N. G. S. Costa, D. R. Couto, C. Cristofolini, A. C. R. Cruz, L. A. Del Neri, M. Pasquo, A. Santos Dias, L. do C. D. Dias, R. Dislich, M. C. Duarte, J. R. Fabricante, F. H. A. Farache, A. P. G. Faria, C. Faxina, M. T. M. Ferreira, E. Fischer, C. R. Fonseca, T. Fontoura, T. M. Francisco, S. G. Furtado, M. Galetti, M. L. Garbin, A. L. Gasper, M. Goetze, J. Gomes-da-Silva, M. F. A. Gonçalves, D. R. Gonzaga, A. C. G. e Silva, A. de C. Guaraldo, E. de S. G. Guarino, A. V. Guislon, L. B. Hudson, J. G. Jardim, P. Jungbluth, S. dos S. Kaeser, I. M. Kessous, N. M. Koch, Y. S. Kuniyoshi, P. H. Labiak, M. E. Lapate, A. C. L. Santos, R. L. B. Leal, F. S. Leite, P. Leitman, A. P. Liboni, D. Liebsch, D. V. Lingner, J. A. Lombardi, E. Lucas, J. dos R. Luzzi, P. Mai, L. F. Mania, W. Mantovani, A. G. Maragni, M. C. M. Marques, G. Marquez, C.

Martins, L. do N. Martins, P. L. S. S. Martins, F. F. F. Mazziero, C. de A. Melo, M. M. F. Melo, A. F. Mendes, L. Mesacasa, L. P. C. Morellato, V. de S. Moreno, A. Muller, M. M. da S. Murakami, E. Cecconello, C. Nardy, M. H. Nervo, B. Neves, M. G. C. Nogueira, F. R. Nonato, A. T. Oliveira-Filho, C. P. L. Oliveira, G. E. Overbeck, G. M. Marcusso, M. L. B. Paciencia, P. Padilha, P. T. Padilha, A. C. A. Pereira, L. C. Pereira, R. A. S. Pereira, J. Pincheira-Ulbrich, J. S. R. Pires, M. A. Pizo, K. C. Pôrto, L. Rattis, J. R. de M. Reis, S. G. dos Reis, T. C. Rocha-Pessôa, C. F. D. Rocha, F. S. Rocha, A. R. P. Rodrigues, R. R. Rodrigues, J. M. Rogalski, R. L. Rosanelli, A. Rossado, D. R. Rossatto, D. C. Rother, C. R. Ruiz-Miranda, F. Z. Saiter, M. B. Sampaio, L. D. Santana, J. S. dos Santos, R. Sartorello, M. Sazima, J. L. Schmitt, G. Schneider, B. G. Schroeder, L. Sevegnani, V. O. S. Júnior, F. R. Silva, M. J. Silva, M. P. P. Silva, R. G. Silva, S. M. Silva, R. B. Singer, G. Siqueira, L. E. Soares, H. C. Sousa, A. Spielmann, V. R. Tonetti, M. T. Z. Toniato, P. S. B. Ulguim, C. Berg, E. Berg, I. G. Varassin, I. B. V. Silva, A. C. Vibrans, J. L. Waechter, E. W. Weissenberg, P. G. Windisch, M. Wolowski, A. Yañez, V. N. Yoshikawa, L. R. Zandoná, C. M. Zanella, E. M. Zanin, D. C. Zappi, V. B. Zipparro, J. P. F. Zorzanelli, and M. C. Ribeiro. 2019. ATLANTIC EPIPHYTES: a data set of vascular and non-vascular epiphyte plants and lichens from the Atlantic Forest. Ecology 100:e02541.

Regolin, A. L., J. J. Cherem, M. E. Graipel, J. A. Bogoni, J. W. Ribeiro, M. H. Vancine, M. A. Tortato, L. G. Oliveira-Santos, F. M. Fantacini, M. R. Luiz, P. V. de Castilho, M. C. Ribeiro, and N. C. Cáceres. 2017. Forest cover influences occurrence of mammalian carnivores within Brazilian Atlantic Forest. Journal of Mammalogy 98:1721–1731.

Reis, R. E., J. S. Albert, F. Di Dario, M. M. Mincarone, P. Petry, and L. A. Rocha. 2016. Fish biodiversity and conservation in South America. Journal of Fish Biology 89:12–47.

Ribeiro, M. C., J. P. Metzger, A. C. Martensen, F. J. Ponzoni, and M. M. Hirota. 2009. The Brazilian Atlantic Forest: How much is left, and how is the remaining forest distributed? Implications for conservation. Biological Conservation 142:1141–1153.

Rios, E., M. Benchimol, K. De Vleeschouwer, and E. Cazetta. 2021a. Spatial predictors and species' traits: evaluating what really matters for medium-sized and large mammals in the Atlantic Forest, Brazil. Mammal Review:mam.12276.

Rios, E., M. Benchimol, P. Dodonov, K. De Vleeschouwer, and E. Cazetta. 2021b. Testing the habitat amount hypothesis and fragmentation effects for medium- and large-sized mammals in a biodiversity hotspot. Landscape Ecology 36:1311–1323.

Riva, F., C. J. Martin, C. Galán Acedo, E. N. Bellon, P. Keil, A. Morán-Ordóñez, L. Fahrig, and A. Guisan. 2024. Incorporating effects of habitat patches into species distribution models. Journal of Ecology:1365-2745.14403.

Riva, F., and S. E. Nielsen. 2020. Six key steps for functional landscape analyses of habitat change. Landscape Ecology 35:1495–1504.

Riva, F., and S. E. Nielsen. 2021. A functional perspective on the analysis of land use and land cover data in ecology. Ambio 50:1089–1100.

Rocchini, D., L. Delucchi, G. Bacaro, P. Cavallini, H. Feilhauer, G. M. Foody, K. S. He, H. Nagendra, C. Porta, C. Ricotta, S. Schmidtlein, L. D. Spano, M. Wegmann, and M. Neteler. 2013. Calculating landscape diversity with information-theory based indices: A GRASS GIS solution. Ecological Informatics 17:82–93.

Rodrigues, R. C., É. Hasui, J. C. Assis, J. C. C. Pena, R. L. Muylaert, V. R. Tonetti, F. Martello, A. L. Regolin, T. V. V. da Costa, M. Pichorim, E. Carrano, L. E. Lopes, M. F. de Vasconcelos, C. S. Fontana, A. L. Roos, F. Gonçalves, C. Banks-Leite, V. Cavarzere, M. A. Efe, M. A. S. Alves, A. Uezu, J. P. Metzger, P. de T. Z. Antas, K. M. P. M. de Ferraz, L. C. Calsavara, A. A. Bispo, H. F. P. Araujo, C. Duca, A. J. Piratelli, L. N. Naka, R. A. Dias, C. A. F. R. Gatto, M. A. V. Vallejos, G. dos R. Menezes, L. Bugoni, H. Rajão, J. J. Zocche, G. Willrich, E. S. da Silva, L. T. Manica, A. de C. Guaraldo, G. Althmann, P. P. Serafini, M. R. Francisco, C. Lugarini, C. G. Machado, F. Marques-Santos, R. Bobato, E. A. de Souza, R. J. Donatelli, C. D. Ferreira, J. C. Morante-Filho, N. D. Paes-Macarrão, A. Macarrão, M. R. Lima, L. I.

Jacoboski, C. Candia-Gallardo, V. B. Alegre, A. E. Jahn, K. V. de C. Barbosa, C. Cestari, J. N. da Silva, N. S. D. Silveira, A. C. V. Crestani, A. P. Petronetto, A. A. A. Bovo, A. D. Viana, A. C. Araujo, A. H. dos Santos, A. C. A. do Amaral, A. Ferreira, A. H. Vieira-Filho, B. C. Ribeiro, C. C. C. Missagia, C. Bosenbecker, C. A. B. Medolago, C. R. R. Espínola, C. Faxina, C. E. C. Nunes, C. Prates, D. T. A. da Luz, D. J. Moreno, D. Mariz, D. Faria, D. Meyer, E. A. Doná, E. R. Alexandrino, E. Fischer, F. Girardi, F. B. Giese, F. L. S. Shibuya, F. A. Faria, F. B. de Farias, F. de L. Favaro, F. J. F. Freitas, F. G. Chaves, F. M. G. Las-Casas, G. L. M. Rosa, G. M. D. L. Torre, G. M. Bochio, G. E. Bonetti, G. Kohler, G. S. Toledo-Lima, G. P. Plucenio, Í. Menezes, I. M. D. Torres, I. C. C. Provinciato, I. R. Viana, J. J. Roper, J. E. Persegona, J. J. Barcik, J. Martins-Silva, J. P. G. Just, J. P. Tavares-Damasceno, J. R. de A. Ferreira, J. R. R. Rosoni, J. E. T. Falcon, L. M. Schaedler, L. B. Mathias, L. R. Deconto, L. da C. Rodrigues, M. A. P. Meyer, M. Repenning, M. A. Melo, M. A. S. de Carvalho, M. Rodrigues, M. F. C. Nunes, M. H. Ogrzewalska, M. L. Gonçalves, M. B. Vecchi, M. Bettio, M. N. da M. Baptista, M. S. Arantes, N. L. Ruiz, P. G. B. Andrade, P. H. L. Ribeiro, P. M. G. Junior, P. Macario, R. Oliveira Fratoni, R. Meurer, R. S. Saint-Clair, R. S. Romagna, R. C. A. Lacerda, R. A. S. Cerboncini, R. B. Lyra, R. Lau, R. C. Rodrigues, R. R. Faria, R. R. Laps, S. L. Althoff, S. Jesus, S. Namba, T. V. Braga, T. Molin, T. P. F. Câmara, T. R. Enedino, U. Wischhoff, V. C. Oliveira, V. Leandro-Silva, V. Araújo-Lima, V. de O. Lunardi, R. F. de Gusmão, J. M. de S. Correia, L. P. Gaspar, R. C. B. Fonseca, P. A. F. P. Neto, A. C. M. M. de Aquino, B. B. de Camargo, B. A. Cezila, L. M. Costa, R. M. Paolino, C. Z. Kanda, E. C. S. Monteiro, J. E. F. Oshima, M. Alves-Eigenheer, M. A. Pizo, L. F. Silveira, M. Galetti, and M. C. Ribeiro. 2019. ATLANTIC BIRD TRAITS: a data set of bird morphological traits from the Atlantic forests of South America. Ecology:e02647.

Rosa, M. R., P. H. S. Brancalion, R. Crouzeilles, L. R. Tambosi, P. R. Piffer, F. E. B. Lenti, M. Hirota, E. Santiami, and J. P. Metzger. 2021. Hidden destruction of older forests threatens

Brazil's Atlantic Forest and challenges restoration programs. Science Advances 7:eabc4547.

Santos, J. P. dos, A. V. L. Freitas, K. S. Brown, J. Y. O. Carreira, P. E. Gueratto, A. H. B. Rosa, G. M. Lourenço, G. M. Accacio, M. Uehara-Prado, C. A. Iserhard, A. Richter, K. Gawlinski, H. P. Romanowski, N. O. Mega, M. O. Teixeira, A. Moser, D. B. Ribeiro, P. F. Araujo, B. K. C. Filgueiras, D. H. A. Melo, I. R. Leal, M. do V. Beirão, S. P. Ribeiro, E. C. B. Cambuí, R. N. Vasconcelos, M. Z. Cardoso, M. Paluch, R. R. Greve, J. C. Voltolini, M. Galetti, A. L. Regolin, T. Sobral-Souza, and M. C. Ribeiro. 2018. Atlantic butterflies: a data set of fruit-feeding butterfly communities from the Atlantic forests. Ecology 99:2875–2875.

Santos, J. P., T. Sobral-Souza, K. S. Brown, M. H. Vancine, M. C. Ribeiro, and A. V. L. Freitas. 2020. Effects of landscape modification on species richness patterns of fruit-feeding butterflies in Brazilian Atlantic Forest. Diversity and Distributions 26:196–208.

Santos, P. M., K. M. P. M. de B. Ferraz, M. C. Ribeiro, B. B. Niebuhr, M. H. Vancine, A. G. Chiarello, and A. P. Paglia. 2022. Natural forest regeneration on anthropized landscapes could overcome climate change effects on the endangered maned sloth (*Bradypus torquatus* , Illiger 1811). Journal of Mammalogy:gyac084.

Scarano, F. R. 2002. Structure, Function and Floristic Relationships of Plant Communities in Stressful Habitats Marginal to the Brazilian Atlantic Rainforest. Annals of Botany 90:517–524.

Scarano, F. R., and P. Ceotto. 2015. Brazilian Atlantic forest: impact, vulnerability, and adaptation to climate change. Biodiversity and Conservation 24:2319–2331.

Schweizer, D., G. Petter, R. Gomes César, S. Ferraz, V. de Souza Moreno, P. H. S. Brancalion, and H. Bugmann. 2022. Natural forest regrowth under different land use intensities and landscape configurations in the Brazilian Atlantic Forest. Forest Ecology and Management 508:120012.

Shennan-Farpón, Y., M. Mills, A. Souza, and K. Homewood. 2022. The role of agroforestry in restoring Brazil's Atlantic Forest: Opportunities and challenges for smallholder farmers.

People and Nature 4:462–480.

Silva, R. F. B. da, M. Batistella, and E. F. Moran. 2016. Drivers of land change: Human-environment interactions and the Atlantic forest transition in the Paraíba Valley, Brazil. Land Use Policy 58:133–144.

Silva, R. R., F. Martello, R. M. Feitosa, O. G. M. Silva, L. P. do Prado, C. R. F. Brandão, E. Z. de Albuquerque, M. S. C. Morini, J. H. C. Delabie, E. C. dos Santos Monteiro, A. Emanuel Oliveira Alves, A. L. Wild, A. V. Christianini, A. Arnhold, A. Casadei Ferreira, A. M. Oliveira, A. D. Santos, A. Galbán, A. A. de Oliveira, A. G. M. Subtil, A. M. Dias, A. E. de Carvalho Campos, A. M. Waldschimidt, A. V. L. Freitas, A. N. Avalos, A. L. S. Meyer, A. F. Sánchez-Restrepo, A. V. Suarez, A. S. Souza, A. C. M. Queiroz, A. J. Mayhé-Nunes, A. da Cruz Reis, B. C. Lopes, B. Guénard, B. M. Trad, B. Caitano, B. Yagound, B. Pereira-Silva, B. L. Fisher, B. L. P. Tavares, B. B. Moraes, B. K. C. Filgueiras, C. Guarda, C. R. Ribas, C. E. Cereto, C. E. L. Esbérard, C. E. G. R. Schaefer, C. I. Paris, C. Bueno, C. J. Lasmar, C. B. da Costa-Milanez, C. J. Lutinski, C. M. Ortiz-Sepulveda, C. T. Wazema, C. S. F. Mariano, C. A. Barrera, C. L. Klunk, D. O. Santana, D. Larrea, D. C. Rother, D. R. Souza-Campana, D. Y. Kayano, D. L. Alves, D. S. Assis, D. Anjos, E. C. B. França, E. F. Santos, E. A. Silva, É. V. Santos, E. B. Koch, E. L. S. Siqueira, É. A. Almeida, E. S. Araujo, E. Villarreal, E. Becker, E. de Oliveira Canedo-Júnior, E. A. Santos-Neto, E. P. Economo, É. S. Araújo-Oliveira, F. Cuezzo, F. S. Magalhães, F. M. Neves, F. B. Rosumek, F. E. Dorneles, F. B. Noll, F. V. Arruda, F. A. Esteves, F. N. Ramos, F. R. M. Garcia, F. S. de Castro, F. Serna, F. R. Marcineiro, F. S. Neves, G. B. do Nascimento, G. de Figueiredo Jacintho, G. P. Camacho, G. T. Ribeiro, G. M. Lourenço, G. R. Soares, G. A. Castilho, G. P. Alves, G. A. Zurita, G. H. Machado Santos, H. C. Onody, H. S. Oliveira, H. L. Vasconcelos, H. F. Paulino-Neto, H. Brant, I. Rismo Coelho, I. J. de Melo Teles e Gomes, I. R. Leal, I. A. Dos Santos, I. C. S. Santos, I. O. Fernandes, I. C. Nascimento, J. M. Queiroz, J. E. Lattke, J. Majer, J. H. Schoereder, J. O. Dantas, J. Andrade-Silva, J. M. Díaz Guastavino, J. Silveira

dos Santos, J. Filloy, J. C. M. Chaul, J. A. Lutinski, K. S. Carvalho, K. S. Ramos, K. L. S. Sampaio, L. A. M. Ribeiro, L. Sousa-Souto, L. N. Paolucci, L. Elizalde, L. R. Podgaiski, L. Chifflet, L. J. Carvalho-Leite, L. A. Calcaterra, L. E. Macedo-Reis, L. F. S. Magnago, M. S. Madureira, M. M. Silva, M. R. Pie, M. Uehara-Prado, M. A. Pizo, M. A. Pesquero, M. A. F. Carneiro, M. A. Busato, M. F. B. de Almeida, M. I. Bellocq, M. Tibcherani, M. S. Casimiro, M. U. V. Ronque, M. M. S. da Costa, M. A. Angotti, M. V. de Oliveira, M. Leponce, M. M. G. Imata, M. F. de Oliveira Martins, M. Antunes Ulysséa, N. B. do Espirito Santo, N. M. Ladino López, N. S. Balbino, N. S. da Silva, N. V. H. Safar, P. L. de Andrade, P. H. S. A. Camargo, P. S. Oliveira, P. Dodonov, P. Luna, P. S. Ward, P. E. Hanisch, P. S. Silva, R. Divieso, R. L. Carvalho, R. B. F. Campos, R. Antoniazzi, R. E. Vicente, R. Giovenardi, R. I. Campos, R. R. C. Solar, R. T. Fujihara, R. de Jesus Santos, R. Fagundes, R. J. Guerrero, R. S. Probst, R. S. de Jesus, R. Silvestre, R. A. López-Muñoz, R. de Souza Ferreira-Châline, R. P. S. Almeida, S. de Mello Pinto, S. Santoandré, S. L. Althoff, S. P. Ribeiro, T. Jory, T. T. Fernandes, T. de Oliveira Andrade, T. P. L. Pereira, T. Gonçalves-Souza, T. S. R. da Silva, V. N. G. Silva, V. M. Lopez, V. R. Tonetti, V. A. F. Nacagava, V. M. Oliveira, W. Dáttilo, W. DaRocha, W. Franco, W. Dröse, W. Antonialli, and M. C. Ribeiro. 2022. ATLANTIC ANTS: a data set of ants in Atlantic Forests of South America. Ecology 103:e03580.

Šímová, P., and K. Gdulová. 2012. Landscape indices behavior: A review of scale effects. Applied Geography 34:385–394.

Sloan, S., C. N. Jenkins, L. N. Joppa, D. L. A. Gaveau, and W. F. Laurance. 2014. Remaining natural vegetation in the global biodiversity hotspots. Biological Conservation 177:12–24.

Song, X.-P., M. C. Hansen, S. V. Stehman, P. V. Potapov, A. Tyukavina, E. F. Vermote, and J. R. Townshend. 2018. Global land change from 1982 to 2016. Nature 560:639–643.

Souza, C. M., J. Z. Shimbo, M. R. Rosa, L. L. Parente, A. A. Alencar, B. F. T. Rudorff, H. Hasenack, M. Matsumoto, L. G. Ferreira, P. W. M. Souza-Filho, S. W. de Oliveira, W. F. Rocha, A. V. Fonseca, C. B. Marques, C. G. Diniz, D. Costa, D. Monteiro, E. R. Rosa, E.

Vélez-Martin, E. J. Weber, F. E. B. Lenti, F. F. Paternost, F. G. C. Pareyn, J. V. Siqueira, J. L. Viera, L. C. F. Neto, M. M. Saraiva, M. H. Sales, M. P. G. Salgado, R. Vasconcelos, S. Galano, V. V. Mesquita, and T. Azevedo. 2020. Reconstructing Three Decades of Land Use and Land Cover Changes in Brazilian Biomes with Landsat Archive and Earth Engine. Remote Sensing 12:2735.

Souza, Y., F. Gonçalves, L. Lautenschlager, P. Akkawi, C. Mendes, M. M. Carvalho, R. S. Bovendorp, H. Fernandes-Ferreira, C. Rosa, M. E. Graipel, N. Peroni, J. J. Cherem, J. A. Bogoni, C. R. Brocardo, J. Miranda, L. Z. da Silva, G. Melo, N. Cáceres, J. Sponchiado, M. C. Ribeiro, and M. Galetti. 2019. ATLANTIC MAMMALS: a data set of assemblages of medium- and large-sized mammals of the Atlantic Forest of South America. Ecology 100:e02785.

Stepinski, T. F., and J. Jasiewicz. 2011. Geomorphons - a new approach to classification of landforms.

Sun, M., W. Li, L. Zhu, Z. Guo, Z. Zhao, N. Meng, M. Han, N. Wang, and X. Zhang. 2025. Degradation in edge forests caused by forest fragmentation. Carbon Research 4:38.

Tambosi, L. R., A. C. Martensen, M. C. Ribeiro, and J. P. Metzger. 2014. A Framework to Optimize Biodiversity Restoration Efforts Based on Habitat Amount and Landscape Connectivity: Optimizing Restoration Based on Landscape Resilience. Restoration Ecology 22:169–177.

Tang, L., and T. T. Werner. 2023. Global mining footprint mapped from high-resolution satellite imagery. Communications Earth & Environment 4:1–12.

Tonetti, V., F. Bocalini, F. Schunck, M. H. Vancine, M. Butti, M. Ribeiro, and M. Pizo. 2024. The Protected Areas network may be insufficient to protect bird diversity in a fragmented tropical hotspot under different climate scenarios. Perspectives in Ecology and Conservation 22:63–71.

Tonetti, V., B. B. Niebuhr, M. Ribeiro, and M. A. Pizo. 2022. Forest regeneration may reduce the

negative impacts of climate change on the biodiversity of a tropical hotspot. Diversity and Distributions 28:2956–2971.

Tonetti, V., J. C. Pena, M. D. Scarpelli, L. S. Sugai, F. M. Barros, P. R. Anunciação, P. M. Santos, A. L. Tavares, and M. C. Ribeiro. 2023. Landscape heterogeneity: concepts, quantification, challenges and future perspectives. Environmental Conservation:1–10.

Turner, M. G., and R. H. Gardner. 2015. Landscape Ecology in Theory and Practice. Springer New York, New York, NY.

Vale, M. M., P. A. Arias, G. Ortega, M. Cardoso, B. F. A. Oliveira, R. Loyola, and F. R. Scarano. 2021. Climate Change and Biodiversity in the Atlantic Forest: Best Climatic Models, Predicted Changes and Impacts, and Adaptation Options. Pages 253–267 *in* M. C. M. Marques and C. E. V. Grelle, editors. The Atlantic Forest: History, Biodiversity, Threats and Opportunities of the Mega-diverse Forest. Springer International Publishing, Cham.

Vancine, M. H., B. B. Niebuhr, R. L. Muylaert, M. Galetti, and M. C. Ribeiro. 2025. *atlanticr*: an ecological and environmental database and R package for biodiversity of Atlantic Forest of South America (v.0.0.9). Zenodo. https://doi.org/10.5281/zenodo.14751252.

Vancine, M. H., K. da S. Duarte, Y. S. de Souza, J. G. R. Giovanelli, P. M. Martins-Sobrinho, A. López, R. P. Bovo, F. Maffei, M. B. Lion, J. W. Ribeiro Júnior, R. Brassaloti, C. O. R. da Costa, H. O. Sawakuchi, L. R. Forti, P. Cacciali, J. Bertoluci, C. F. B. Haddad, and M. C. Ribeiro. 2018. ATLANTIC AMPHIBIANS: a data set of amphibian communities from the Atlantic Forests of South America. Ecology 99:1692–1692.

Vancine, M. H., R. L. Muylaert, B. B. Niebuhr, J. E. D. F. Oshima, V. Tonetti, R. Bernardo, C. De Angelo, M. R. Rosa, C. H. Grohmann, and M. C. Ribeiro. 2024. The Atlantic Forest of South America: Spatiotemporal dynamics of the vegetation and implications for conservation. Biological Conservation 291:110499.

Varassin, I. G., K. Agostini, M. Wolowski, and L. Freitas. 2021. Pollination Systems in the Atlantic Forest: Characterisation, Threats, and Opportunities. Pages 325–344 *in* M. C. M. Marques

and C. E. V. Grelle, editors. The Atlantic Forest: History, Biodiversity, Threats and Opportunities of the Mega-diverse Forest. Springer International Publishing, Cham.

Vega, G. C., L. R. Pertierra, and M. Á. Olalla-Tárraga. 2017. MERRAclim, a high-resolution global dataset of remotely sensed bioclimatic variables for ecological modelling. Scientific Data 4:170078.

Vitule, J. R. S., T. V. T. Occhi, L. Carneiro, V. S. Daga, F. A. Frehse, L. A. V. Bezerra, S. Forneck, H. S. de Pereira, M. O. Freitas, C. G. Z. Hegel, V. Abilhoa, M. T. Grombone-Guaratini, J. Queiroz-Sousa, V. R. Pivello, D. M. Silva-Matos, I. Oliveira, L. F. Toledo, M. A. V. Vallejos, R. D. Zenni, A. G. P. Ford, and R. R. Braga. 2021. Non-native Species Introductions, Invasions, and Biotic Homogenization in the Atlantic Forest. Pages 269–295 *in* M. C. M. Marques and C. E. V. Grelle, editors. The Atlantic Forest: History, Biodiversity, Threats and Opportunities of the Mega-diverse Forest. Springer International Publishing, Cham.

Viveiros de Castro, E. B., A. M. Lanna, A. C. Lobo, F. Feliciani, R. B. Bradford, J. L. do Nascimento, and C. E. V. Grelle. 2021. The Atlantic Forest Trail: Reconnecting People, Biodiversity, and Protected Areas. Pages 403–419 *in* M. C. M. Marques and C. E. V. Grelle, editors. The Atlantic Forest. Springer International Publishing, Cham.

Vogt, P., K. H. Riitters, C. Estreguil, J. Kozak, T. G. Wade, and J. D. Wickham. 2007. Mapping Spatial Patterns with Morphological Image Processing. Landscape Ecology 22:171–177.

Williams, J. J., and T. Newbold. 2020. Local climatic changes affect biodiversity responses to land use: A review. Diversity and Distributions 26:76–92.

Willmer, J. N. G., T. Püttker, and J. A. Prevedello. 2022. Global impacts of edge effects on species richness. Biological Conservation 272:109654.

Young, H. S., D. J. McCauley, M. Galetti, and R. Dirzo. 2016. Patterns, Causes, and Consequences of Anthropocene Defaunation. Annual Review of Ecology, Evolution, and Systematics 47:333–358.

Zou, Y., T. W. Crowther, G. R. Smith, H. Ma, L. Mo, L. Bialic-Murphy, P. Potapov, K. A. Gawecka, C. Xu, P. J. Negret, T. Lauber, Z. Wu, D. Rebindaine, and C. M. Zohner. 2025. Fragmentation increased in over half of global forests from 2000 to 2020. Science 389:1151–1156.

Zupo, T., J. Lazzarotto Freitas, D. Almeida Dos Reis, and M. Ferreira De Siqueira. 2022. Trends and knowledge gaps on ecological restoration research in the Brazilian Atlantic Forest. Restoration Ecology 30:e13645.

Zwiener, V. P., A. A. Padial, M. C. M. Marques, F. V. Faleiro, R. Loyola, and A. T. Peterson. 2017. Planning for conservation and restoration under climate and land use change in the Brazilian Atlantic Forest. Diversity and Distributions 23:955–966.
